# In-situ evidence of volcanic ash aggregation during fallout from combined ground- and UAS-based observations

**DOI:** 10.1038/s41598-026-45460-x

**Published:** 2026-03-26

**Authors:** Simon Thivet, Riccardo Simionato, Allan Fries, Carolina Díaz-Vecino, Jonathan Lemus, Valentin Fréret-Lorgeril, Alexandros P. Poulidis, Masato Iguchi, Costanza Bonadonna

**Affiliations:** 1 Department of Earth Sciences, University of Geneva, Geneva, Switzerland; 2 Department of Computer Science, University of Geneva, Geneva, Switzerland; 3 Laboratoire Magmas et Volcans, Université Clermont Auvergne, CNRS, IRD, OPGC, 63000 Clermont-Ferrand, France; 4 Institute of Environmental Physics, University of Bremen, 28359 Bremen, Germany; 5 Disaster Prevention Research Institute, Kyoto University, Kagoshima, Japan

**Keywords:** Environmental sciences, Natural hazards, Solid Earth sciences

## Abstract

This study investigates the sedimentation of volcanic particles from low-altitude (< 2 km a.s.l.), near-daily ash plumes and clouds at Sakurajima volcano (Japan). Plume dynamics were monitored using imagery (visible wavelength) and geophysical (ash discharge rates) data. Ash fallout was characterized by using ground-based (disdrometer, particle electrical charge sensor, and sampling) and drone-mounted (optical particle counter, atmospheric sensor, and sampling) instruments. A comparison of particle size distributions and aggregate proportions between samples collected by drone 500 m above the take-off sites and those collected on the ground shows that aggregation develops rapidly during sedimentation. This process involves collisions between coarse ash (up to 1 mm) and fine ash particles (< 63 µm). Particle binding is promoted by electrostatic attraction (forming particle clusters) or high atmospheric humidity (forming accretionary pellets). These results provide innovative in-situ evidence of ash aggregation, offering new insights into its dynamics in natural settings, crucial for improving volcanic ash dispersion forecasting.

**Supplementary Information:**

The online version contains supplementary material available at 10.1038/s41598-026-45460-x.

## Introduction

Explosive volcanic eruptions generate multiple hazards (e.g., ballistics, tephra fallout, pyroclastic density currents) that shape landscapes, affect ecosystems, impact the climate, and threaten human populations and infrastructure^1–3^. In particular, tephra (i.e., particles associated with explosive volcanic eruptions) are dispersed in the atmosphere and deposited on the ground based on their size, shape, and density^4–6^. Nonetheless, collective sedimentation processes, such as particle aggregation or Settling-Driven Gravitational Instabilities (SDGIs), can significantly affect tephra settling patterns, making fine volcanic ash (tephra particles < 63 µm in average diameter) fall closer to the volcano than expected^7–9^.

As soon as volcanic ash particles (< 2 mm in average diameter) are formed in volcanic conduits, they start interacting through collision and binding, forming aggregates of varying size, shape, and structure^10–12^. Various theoretical models^7,13,14^, numerical simulations^15–17^, and laboratory experiments^18–20^ have been carried out to investigate aggregation processes. Particle clusters (PCs) are typically formed because of Van der Waals forces and electrostatic charges between particles, while accretionary pellets (APs) are associated with water condensation, in which surface tension of water and capillary forces play cohesive and stabilizing roles. Both PC and AP categories are subdivided into 3 and 4 subcategories, respectively^10,12,21^. PC1s are irregular-shaped and poorly structured ash clusters, while PC2s are described as core particles coated by scattered fine ash, and PC3s are described as ash clusters with an internal core acting as a nucleus. In addition, AP1s, cAP1s, AP2s, and AP3s are described as sub-spherical and poorly structured pellets, cored but poorly structured pellets, pellets with concentric structure (also known as accretionary lapilli), and liquid water drops containing ash particles, respectively.

Despite this wealth of knowledge on particle aggregation and sedimentation from large, infrequent volcanic plumes and clouds, limited information exists for particle aggregation associated with less intense but more frequent events. Through detailed ground and Unoccupied Aircraft System (UAS)-based measurements and sampling, we aim to characterize tephra sedimentation from low-intensity, near-daily plumes using Sakurajima volcano as a case study and provide key insights into associated particle aggregation. More specifically, this study aims to:

- Determine whether volcanic ash aggregation occurs during low-intensity events,
- Identify when and where aggregation takes place,
- Infer the dominant ash aggregation mechanisms responsible for different aggregate types.

**Fig. 1:**
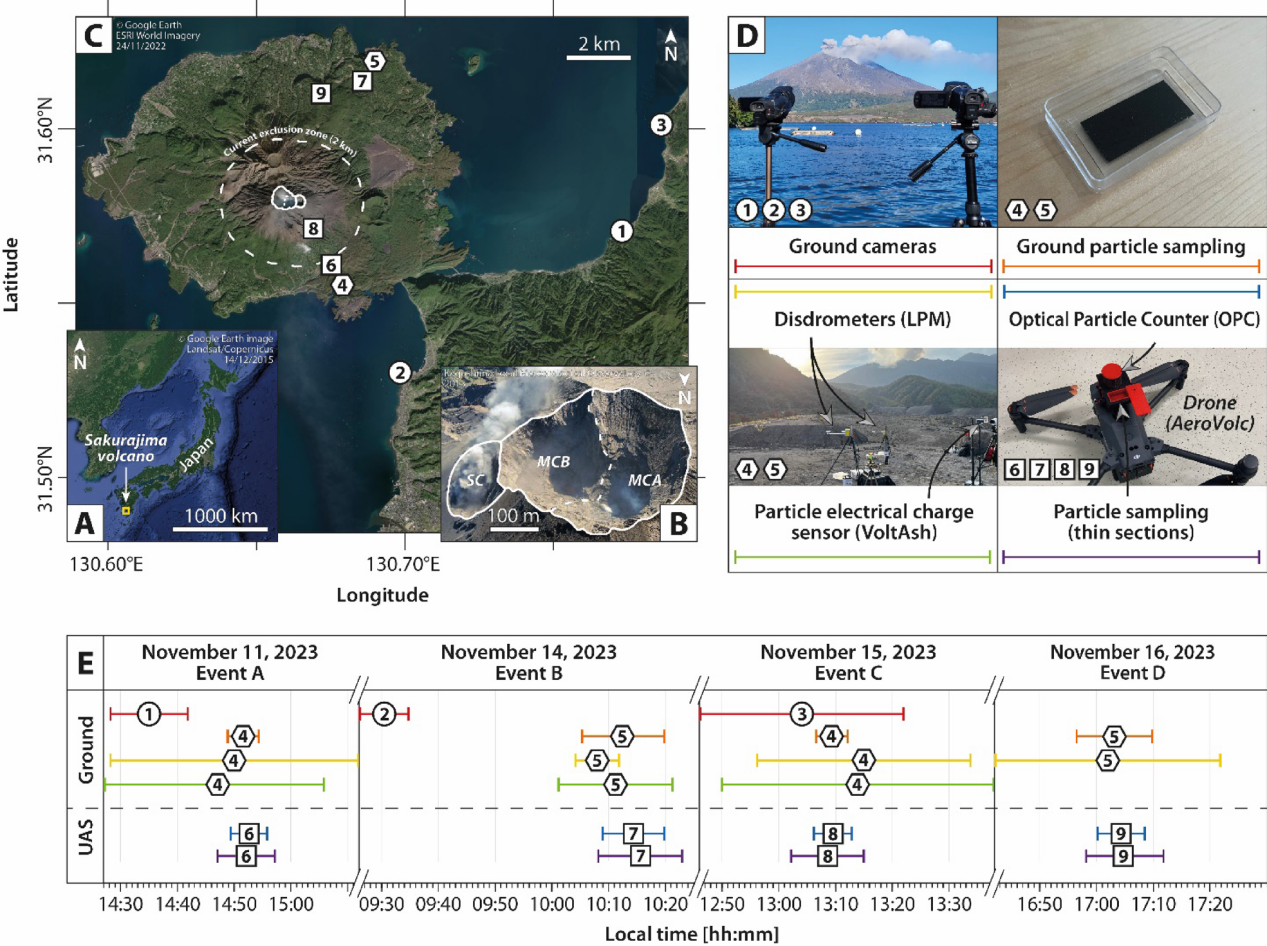
Map of the studied area and timeline of the different field instruments used in this study. (**A**) Localization of Sakurajima at a global scale. Image taken from the open-source Google Earth software v.7.3.6.10441 (https://www.google.com/earth/), 14/12/2025, and modified with the open-source GIMP software v.3.0.8 (https://www.gimp.org/). (**B**) Aerial photo of the active craters of Sakurajima (MCA: Minamidake crater vent A; MCB: Minamidake crater vent B; SC: Showa crater). Image courtesy of the Kagoshima Local Meteorological Observatory (Japan Meteorological Agency), 2015, and modified with the open-source GIMP software v.3.0.8 (https://www.gimp.org/). The image is also under the Creative Commons Attribution 4.0 License in^32^. (**C**) Map of Sakurajima edifice. White lines delimit the summit craters shown in (**B**). Numbered areas show the positions of the devices used in this study. Circles show ground-based and relatively distant sites where cameras were settled. Hexagons show ground-based and relatively proximal sites where in-situ measurements were performed. Squares show Unoccupied Aerial System (UAS)-based and proximal sites where in-situ measurements were performed. Image taken from the open-source Google Earth software v.7.3.6.10441 (https://www.google.com/earth/), 24/11/2022, and modified with the open-source GIMP software v.3.0.8 (https://www.gimp.org/). (**D**) Photos of the different field instruments used in this study. Numbers correspond to the positions shown in (**C**). (**E**) Timeline of field instrument usage. Numbers correspond to the positions shown in (C), and colors correspond to the ones shown in (**D**).

Sakurajima volcano, Japan (Fig. 1A–C), provides an ideal context for our research objectives, as it is known for generating a variety of volcanic aggregates when producing volcanic plumes and clouds^22–24^. In addition, the recent Sakurajima activity is known for its continuous degassing, intersected by frequent ash-venting and Vulcanian explosions . Ash-venting is defined as the low-energy release of ash and gas, which does not involve significant magma fragmentation nor strong ejection of material into the atmosphere^25^. In contrast, Vulcanian explosions are characterized by the high-energy release of tephra and gas, involving significant magma fragmentation and relatively strong ejection of material^26^. Both phases produce volcanic plumes and clouds of various durations, heights, and particle concentrations.

**Fig. 2:**
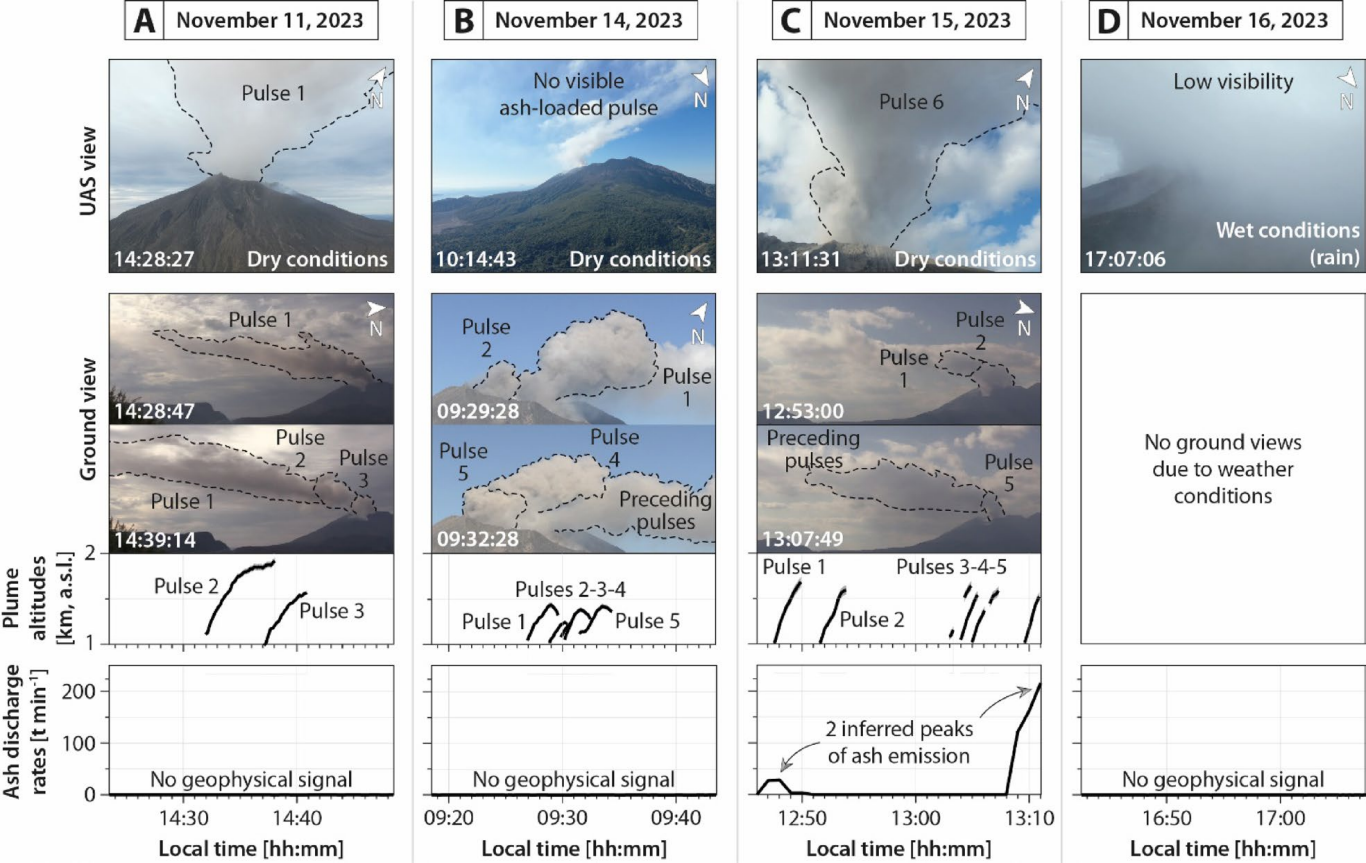
Illustration of the studied events from Unoccupied Aerial System (UAS)-based (first row) and ground-based (second row) images, with plume altitudes (third row), as well as ash discharge rates at vent (fourth row). (**A**) Event A (November 11, 2023). (**B**) Event B (November 14, 2023). (**C**) Event C (November 15, 2023). (**D**) Event D (November 16, 2023).

**Fig. 3:**
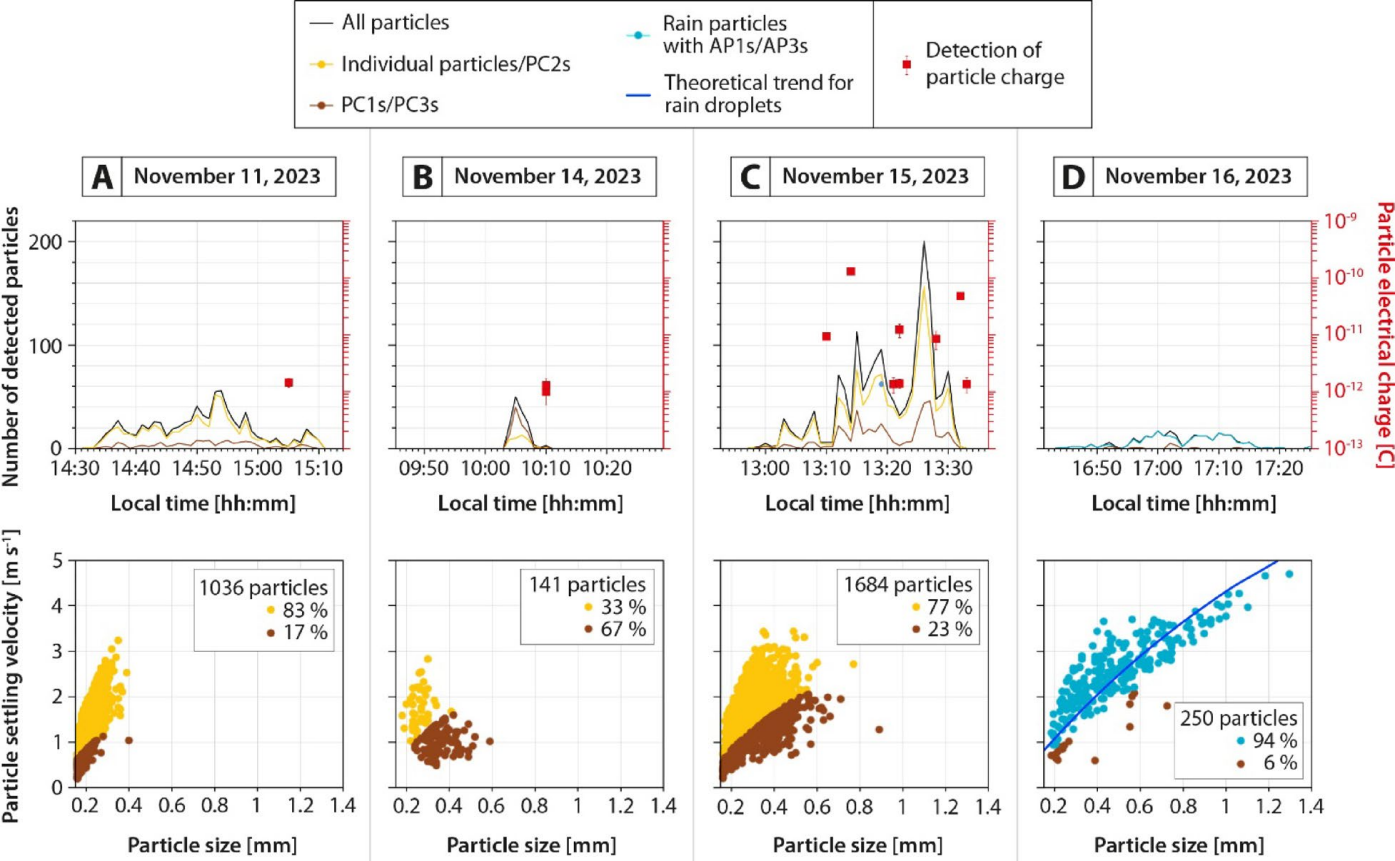
Disdrometer data acquired beneath the volcanic clouds and at ground level, showing the number of detected particles every minute (top row), and the corresponding particle settling velocity as a function of particle size (bottom row). Measured electrical charges of particles are also indicated by red squares (top row). (**A**) Event A (November 11, 2023). (**B**) Event B (November 14, 2023). (**C**) Event C (November 15, 2023). (**D**) Event D (November 16, 2023).

**Fig. 4:**
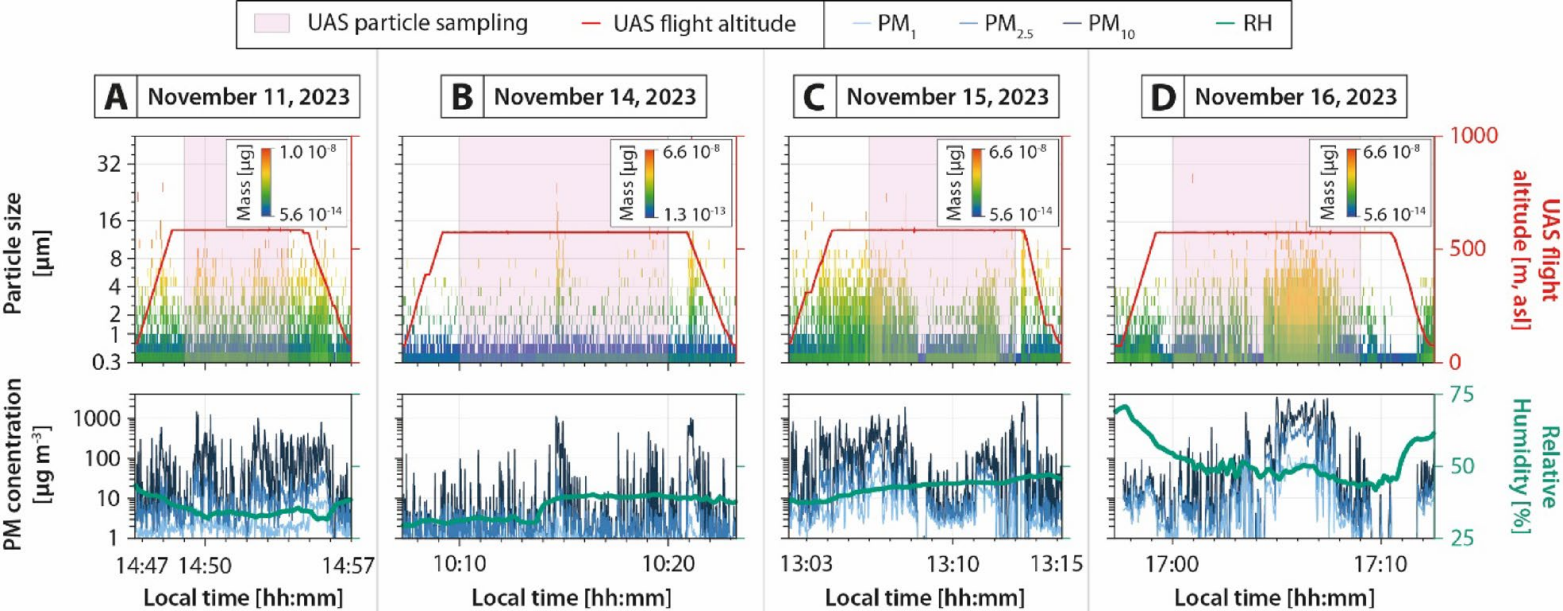
Optical particle counter (OPC) data acquired below the volcanic clouds, at Unoccupied Aircraft System (UAS) flight altitude (represented by the red lines), showing the grain size and mass evolutions as a function of time (top row), as well as the corresponding PM_1_, PM_2.5_, and PM_10_ concentrations as a function of time (bottom row). In-situ atmospheric Relative Humidity (RH) is indicated by the green lines (bottom row). (**A**) Event A (November 11, 2023). (**B**) Event B (November 14, 2023). (**C**) Event C (November 15, 2023). (**D**) Event D (November 16, 2023).

**Fig. 5:**
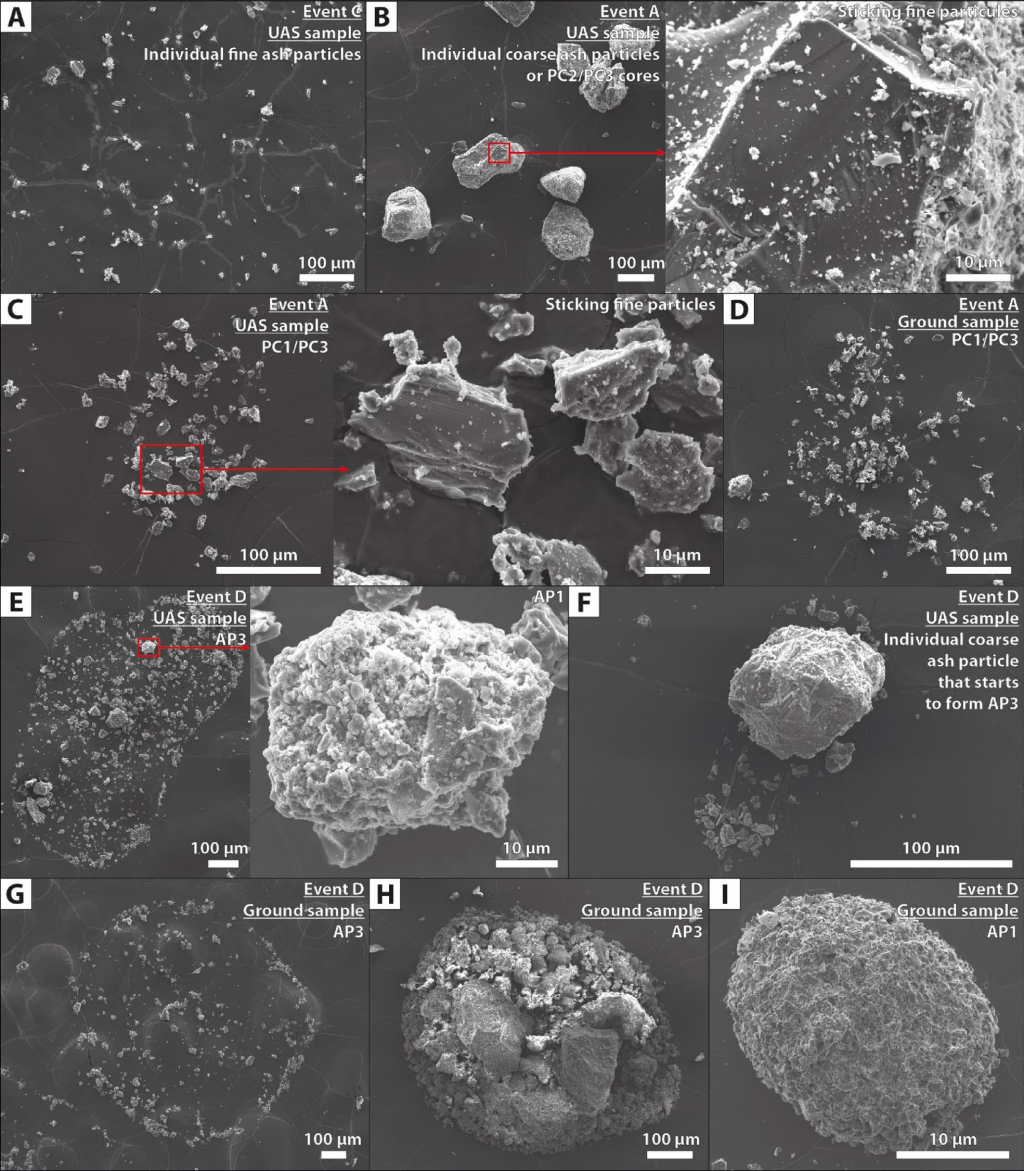
Examples of individual and aggregated particles collected on adhesive carbon tape, both at UAS flight altitude and ground level. Images are from Scanning Electron Microscopy (SEM) acquired in Secondary Electron (SE) mode. (**A**) Individual ash particles < 63 µm. (**B**) Individual ash particles > 63 µm or cores of PC2/PC3 aggregates. (**C**) and (**D**) PC1/PC3 aggregates. (**E**), (**F**), and (**G**) AP3. (**H**) and (**I**) AP1.

**Fig. 6:**
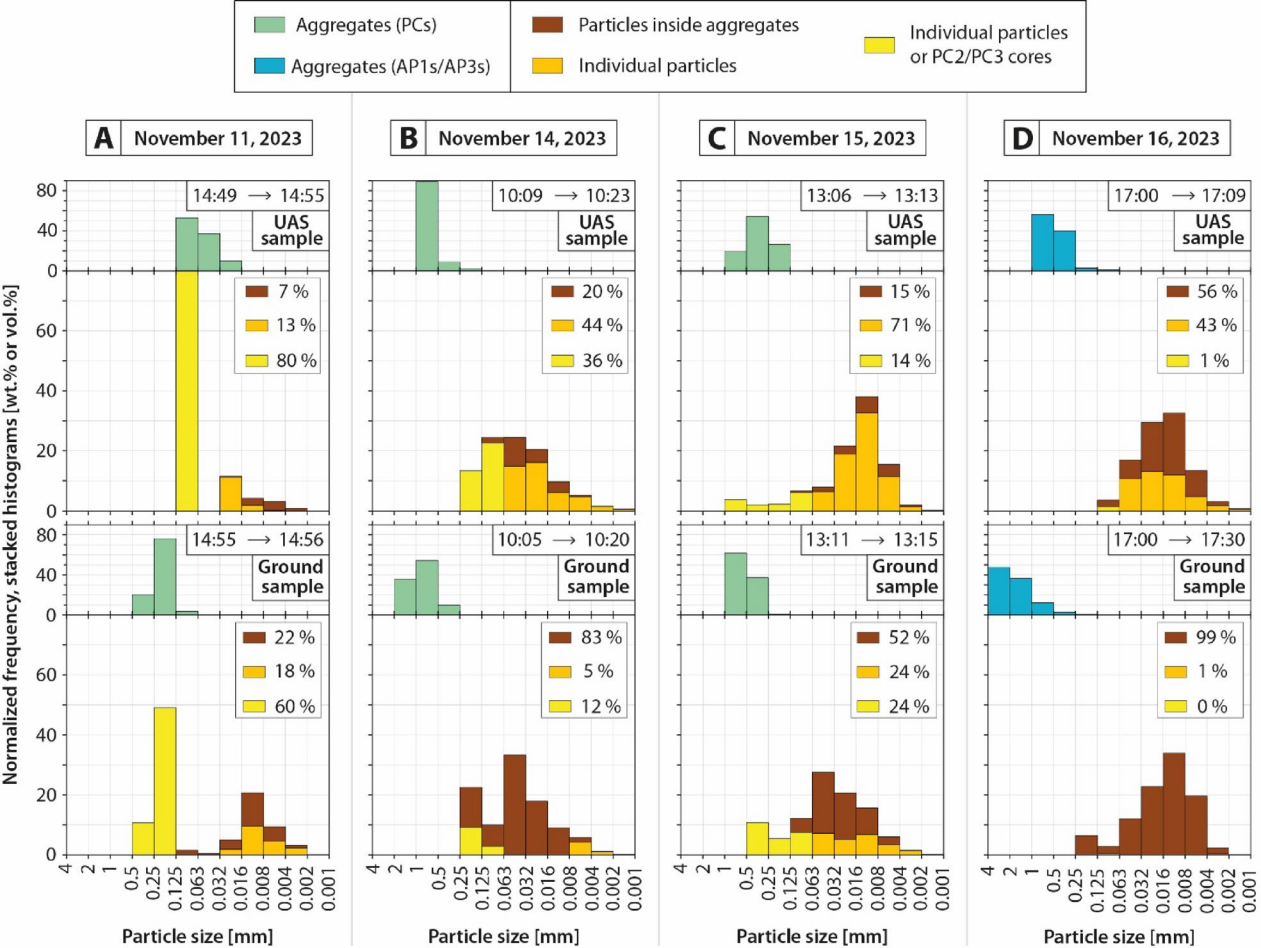
Grain size distributions of the samples collected on adhesive carbon tape, both at UAS sampling altitude (top row) and ground level (bottom row). Also note that sampling time windows are indicated for each sample. Green histograms represent the apparent aggregate sizes. Brown, orange, and yellow histograms represent individual particle sizes, but from different components, depending on whether the particle is individual or part of an aggregate. (**A**) Event A (November 11, 2023). (**B**) Event B (November 14, 2023). (**C**) Event C (November 15, 2023). (**D**) Event D (November 16, 2023).

**Fig. 7:**
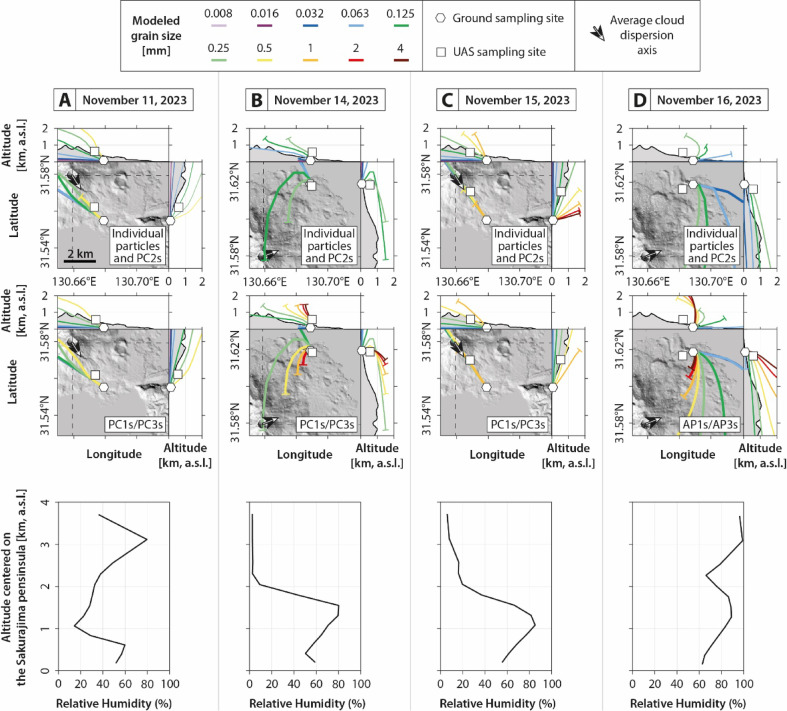
Modeled backward particle trajectories, from ground sampling sites to the maximum plume altitudes. Individual particles/PC2 aggregates (top row), as well as PC1/PC3/AP1/AP3 aggregates (middle row) are modeled. Each colored line corresponds to a specific grain size. Only observed grain sizes from the collected samples are displayed. Also, grain sizes < 8 µm are not displayed for clarity and behave similarly to the 8 µm size bin (i.e., aerosols). Relative Humidity (RH) values from ERA5 dataset as a function of altitude, centered and averaged on the Sakurajima peninsula, are also displayed (bottom row). (**A**) Event A (November 11, 2023). (**B**) Event B (November 14, 2023). (**C**) Event C (November 15, 2023). (**D**) Event D (November 16, 2023).

**Fig. 8:**
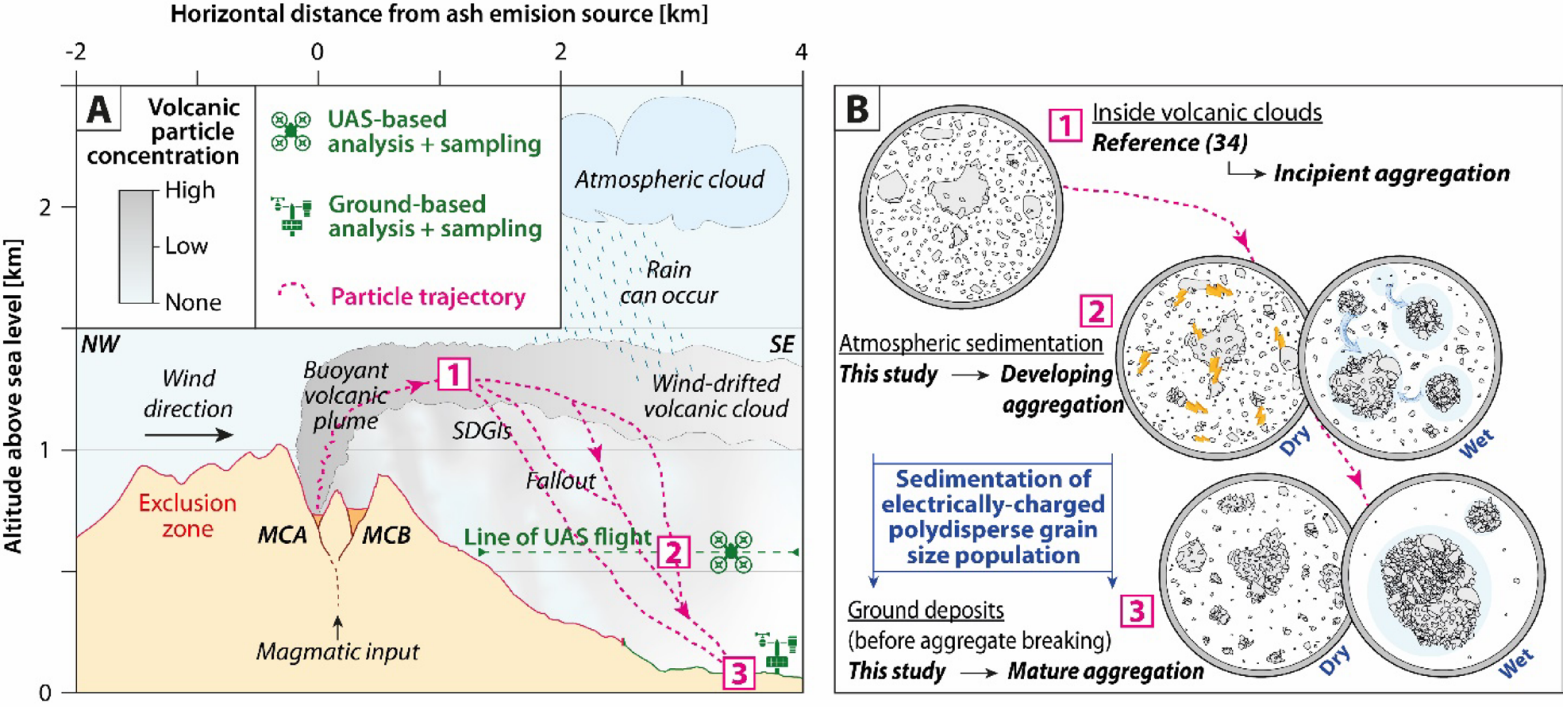
Sketches of the investigated processes. (**A**) Cross-section of Sakurajima. Numbered steps correspond to the life step of volcanic ash particles, which are detailed in (**B**). MCA: Minamidake crater vent A; MCB: Minamidake crater vent B. (**B**) Syn-sedimentation processes at particle scale. Dry conditions indicate no rainfall, whereas wet conditions denote the presence of rain.

A field campaign was conducted from November 9 to 19, 2023, which forms the basis of this work. This study examines four distinct volcanic events related to ash emissions, dispersion, and sedimentation. They were characterized based on a multidisciplinary approach combining several sampling devices and sensors. At ground level, high-resolution visible-wavelength cameras were set in relatively distant sites (7 to 12 km from the active vents, cf. symbols numbered 1 to 3 in Fig. 1C–E) to qualify the general dimensions of the generated plumes and clouds in space and time, as well as to quantify the plume heights (Fig. 2), following the approaches of^27,28^. Ground deformation and volcanic tremor acquired and automatically processed by the Sakurajima Volcano Observatory (SVO), following the approach of^29^, provide an estimation of the ash discharge rates at vent related to each of these events (Fig. 2). Optical disdrometers were placed in relatively proximal sites (from 3 to 5 km from active the vents) and under the sedimenting clouds (cf. symbols numbered 4 to 5 in Fig. 1C–E) to monitor the sedimentation rate, size, and settling velocity of relatively coarse (> 150 µm) particles (Fig. 3), following the approach of^30^. In addition, the VoltAsh electrical charge measurement system^31^ was installed in the same sites as the disdrometers (symbols numbered 4 to 5 in Fig. 1C–E) to record the electrical charge of falling particles (Fig. 3). In parallel, the AeroVolc UAS^32^ was systematically deployed 500 m above its take-off sites (which are located between 75 and 85 m a.s.l.) and at varying horizontal distances from the vent (from 1 to 5 km from the active vents, cf. symbols 7–9 in Fig. 1C–E). At these positions, the UAS monitored atmospheric concentrations of relatively fine particles (< 40 µm) using an Optical Particle Counter (OPC, Fig. 4), and collected tephra fallout samples in a fully controlled manner on thin sections covered with adhesive carbon tape. Similar thin sections were displayed at ground level next to the disdrometers (cf. symbols numbered 5 to 6 in Fig. 1C–E), following the approaches of^11,21,33^. Samples collected both with the UAS and at ground level were imaged by Scanning Electron Microscopy (SEM) in laboratory at the University of Geneva (Fig. 5), then analyzed and compared in terms of grain size distributions and proportion of particles counted within aggregates (Fig. 6), following an adapted image nesting approach from^34^. Numerical simulations of backward particle trajectories are presented for each event (Fig. 7) to assess the comparability between ground- and UAS-based samples, and to investigate potential transport pathways as a function of particle properties and atmospheric conditions, using the LagTrack model^35,36^. Finally, the results and discussion lead to the development of a conceptual model illustrating the ash aggregation process throughout the atmospheric journey of the studied ash particles (Fig. 8).

## Results

### Volcanic activity and atmospheric conditions

Ground-based images were analyzed to quantify the altitude of the emitted plumes and to estimate the overall spatial and temporal evolutions of the associated clouds in the atmosphere. Associated ash discharge rates at vent were also estimated to distinguish between Vulcanian explosions and ash-venting activity (Fig. 2, also cf. supplementary material Data_S1 for raw data).

The first studied event (event A, Fig. 2A) occurred in the afternoon of November 11, 2023, under dry atmospheric conditions (i.e., absence of rain), and was characterized by the production of three main particle emission pulses between 14:25 and 14:41. The onset of the first pulse was not recorded by the ground-based camera. The second and third pulses reached a maximum altitude of 1.9 and 1.6 km above sea level (a.s.l.), respectively. Associated clouds drifted towards the south-southeast (333°N, averaged conventional wind direction) at slightly increasing altitudes, placing the ground measuring and sampling site (i.e., Arimura lava observation deck, cf. symbol numbered 4 in Fig. 1C, E) within the particle sedimentation area. Estimated ash discharge rates at the vent are below the detection limit, because no ground deformation or volcanic tremor was recorded by the SVO^29^, suggesting that the nature of this event was ash-venting only (i.e., non-Vulcanian ash emission episode).

The second event (event B, Fig. 1B) occurred in the morning of November 14, 2023, under dry atmospheric conditions, and was marked by five main particle emission pulses between 09:27 and 09:34. These pulses reached a maximum altitude of 1.4 km a.s.l., and associated clouds were advected towards the east-northeast (249°N) at relatively constant altitudes, which resulted in the ground measuring and sampling site (cf. symbol numbered 5 in Fig. 1C, E) being located within the particle sedimentation area. Similarly to the first event, only ash-venting was observed for this case study.

The third event (event C, Fig. 2C) occurred on November 15, 2023, under dry atmospheric conditions, and featured six main particle emission pulses between 12:47 and 13:11. These pulses reached a maximum altitude of 1.7 km a.s.l., with the resulting plume bent-over towards the south–southeast (349° N) at slightly increasing altitudes, thereby positioning the ground measuring and sampling site (Arimura lava observation deck, cf. symbol numbered 4 in Fig. 1C, E) in the particle fallout area. Contrary to the events A and B, slight ground deformation and volcanic tremor were recorded by the SVO, suggesting low-intensity Vulcanian activity^29^ associated with maximum ash discharge rates of ca. 30 and 200 t min^−1^, for pulses 1 and 6, respectively.

The fourth and last studied event (event D, Fig. 2D) occurred on November 16, 2023, under wet atmospheric conditions (i.e., rainfall occurrence), and was not characterized through the ground-based cameras due to low visibility conditions. However, on-site observations suggested a global cloud dispersion towards the north-northwest (166° N), placing the ground sampling and measuring site (cf. symbol number five in Fig. 1C, E) within the particle sedimentation area. Similarly to the first and second events, only ash-venting is observed for this case study.

### Ash fallout at ground level

Data from disdrometers and electrical charge sensors were analyzed to characterize ash particles during their final stage of sedimentation, focusing on particle number, size, velocity, and electrical charge. This analysis aimed to quantify the physical properties of the relatively coarse fraction (> 150 µm) of particles settling at ground level (Fig. 3; see also Supplementary Data_S2 for raw data).

First, note that a systematic delay of 5 to 37 min between pulse generations (Fig. 2) and the arrival of the first particles detected at ground level (Fig. 3) is observed, which is due to variable particle transport durations influenced by the variable distance between the sampling points and the eruption source, particle downward velocity, plume height, as well as wind direction and velocity.

Disdrometer measurements suggest the presence of both individual and aggregated particles. Data filtering from^30^, which is based on the theoretical settling velocity of particles of known densities and shapes can distinguish between noncoherent values, relatively dense individual particles/PC2 aggregates (cf. orange dots in Fig. 3A–C), and less dense PC1/PC3 aggregates (cf. brown data in Fig. 3A–C) during dry atmospheric conditions (i.e., events A, B, and C). Note that rainfall occurred during event D (Fig. 3D), and most of the detected particles follow the typical pattern of rain droplets (cf. dark blue line in Fig. 3D), potentially containing volcanic ash (i.e., AP1/AP3 aggregates, cf. blue dots in Fig. 3D), which are distinguished from scarcer and less dense PC1/PC3 aggregates (cf. brown data in Fig. 3D). Note that cAP1s and AP2s were not observed in this study.

More specifically, event A (Fig. 3A) shows a relatively symmetric sedimentation rate pattern between 14:30 and 15:11 (duration of 41 min), with a peak of detected particles at 14:54 (58 particles per minute). The size of detected particles varies between 150 (lower limit of detection) and 400 µm, with particle settling velocities ranging from 0.2 to 3.2 m s^−1^. Data filtering shows the dominant presence of 83% of preferentially individual particles/PC2 aggregates and 17% of preferentially PC1/PC3 aggregates. A single electrical charge was measured at 1.4 10^−12^ C.

Event B (Fig. 3B) shows a relatively peaked sedimentation rate pattern between 10:03 and 10:11 (duration of 8 min), with a maximum of detected particles at 10:05 (50 particles per minute). Their size ranges from 180 to 600 µm, with particle settling velocities ranging from 0.5 to 2.9 m s^−1^. Data filtering shows that PC1/PC3 aggregates are dominant (67%) compared to individual particles/PC2 aggregates (33%). Two electrical charges were measured at 1.0 10^−12^ and 1.3 10^−12^ C.

Event C (Fig. 3C) shows a relatively asymmetric sedimentation rate pattern between 12:57 and 13:33 (duration of 36 min), with a peak of detected particles at 13:26 (200 particles per minute). The size of detected particles ranges from 150 to 900 µm, with settling velocities ranging from 0.2 to 3.5 m s^−1^. Data filtering shows that individual particles/PC2 aggregates are dominant (77%) compared to PC1/PC3 aggregates (33%). Eight particle electrical charges were measured between 1.3 × 10^−12^ and 1.3 × 10^−10^ C.

Event D (Fig. 3D) shows a relatively symmetric sedimentation rate pattern between 16:43 and 17:25 (duration of 43 min), with a peak of detected particles at 17:00 and 17:02 (18 particles per minute). Their size varies between 180 to 1300 µm, with settling velocities ranging from 0.6 to 4.8 m s^−1^. The majority of particles (94%) are interpreted as rain droplets potentially carrying AP1/AP3 aggregates as they follow the theoretical trend of rain droplets^37^ shown by the blue line (Fig. 3D), while 6% are identified as PC1/PC3 aggregates. Electrical charges of particles were not recorded for this event due to adverse atmospheric conditions.

### Ash fallout above ground level

Data acquired by the UAS-mounted OPC (cf. supplementary material Data_S3 for the UAS flight trajectories and data) were analyzed to characterize falling ash particles during their airborne sedimentation in terms of particle size, mass, and concentration, in order to quantify the physical properties of the relatively fine (< 40 µm) fallout above ground level (Fig. 4, also cf. supplementary material Data_S4 for raw data). Atmospheric Relative Humidity (RH) is also retrieved from the OPC measurements to evaluate the atmospheric conditions of the collected samples.

These data show specific zones of high concentrations of fine particles compared to the atmospheric background. Particulate matter (PM) concentrations were also determined from these measurements (PM_1_, PM_2.5_, and PM_10_ corresponding to particles < 1, < 2.5, and < 10 µm, respectively). In the following description, only particle concentration data acquired at stable altitudes (i.e., 500 m above the UAS take-off points, cf. red line in Fig. 4) and positions are considered.

During event A (Fig. 4A), numerous and brief peaks of particles from 0.3 to 14 µm are visible between 14:48 and 14:54, highlighting the discrete presence of fine particles below the volcanic cloud. This signal leads to highly oscillating PM concentrations, from background values (ca. 2, 5, and 10 µg m^−3^ for PM_1_, PM_2.5_, and PM_10_, respectively) to peak values up to 10 (ca. 5 times the background values), 60 (ca. 12 times the background values), and 1400 µg m^−3^ (ca. 140 times the background values) for PM_1_, PM_2.5_, PM_10_, respectively. RH values measured throughout the UAS flight ranged from 31 to 43%, indicating moderately dry (dry air is typically defined as 20 to 40% of RH) to slightly moist atmospheric conditions (moist air is typically defined as 40 to 80% of RH), consistent with the optical observations (Fig. 2A).

During event B (Fig. 4B), only one main peak of particles from 0.3 to 28 µm is evidenced from the atmospheric background between 10:09 and 10:21. This peak is observed at ca. 10:15 and its duration can be estimated at ca. 20 s and results in PM_1_, PM_2.5_, PM_10_ concentrations of 3 (ca. twice the background values), 50 (ca. 10 times the background values), and 1050 µg m^−3^ (ca. 100 times the background values), respectively. RH measured along the whole UAS flight ranged between 29 and 41%, indicating relatively dry to slightly moist atmospheric conditions, in agreement with the optical observations (Fig. 2B).

During event C (Fig. 4C), numerous and brief peaks of particles from 0.3 to 40 µm are observed between 13:04 and 13:08, and also between 13:11 and 13:12 with particles from 0.3 to 12 µm. Ultimately, two main distinct peaks of particles from 0.3 to 14 µm are distinguishable between 13:12 and 13:14, with a duration of ca. 20 s for each of them. The overall signal leads to an increase of PM_1_, PM_2.5_, PM_10_ concentrations up to 50 (ca. 25 times the background values), 280 (ca. 50 times the background values), and 1900 µm m^−3^ (ca. 190 times the background values), respectively. RH measured throughout the UAS flight ranged from 37 to 47%, indicating moderately dry to slightly moist atmospheric conditions, consistent with the optical observations (Fig. 2C).

During event D (Fig. 4D), numerous and brief peaks of particles from 0.3 to 28 µm are first observed between 17:00 and 17:04. Then, a highly concentrated patch of particles from 0.3 to 40 µm is observed between 17:04 and 17:08. The overall signal leads to an increase of PM_1_, PM_2.5_, PM_10_ concentrations up to 170 (ca. 80 times the background values), 1100 (ca. 220 times the background values), and 3420 µg m^−3^ (ca. 340 times the background values), respectively. RH measured along the whole UAS flight ranged between 39 and 64%, indicating moderately dry to relatively moist atmospheric conditions, in agreement with the optical observations (Fig. 2D).

### Particle size and aggregate proportion evolutions from Unoccupied Aircraft System (UAS) to ground samples

Collected samples both with the UAS and at ground level (Fig. 5, also cf. supplementary material Data_S5 for raw data) were manually analyzed in terms of grain size and componentry (i.e., particle classification depending if each grain is individual or part of an aggregate) following an adapted nested imaging workflow from^34^ to quantify and compare the size of the apparent aggregates on the thin sections (cf. green histograms in Fig. 6), the proportions of ash particles inside aggregates (cf. brown histograms in Fig. 6), the proportions of individual particles < 63 µm (cf. orange histograms in Fig. 6), and the proportions of undetermined particles (i.e., either individual particles > 63 µm or cores of PC2/PC3 aggregates, cf. yellow histograms in Fig. 6, also cf. supplementary material Data_S6 for raw data). In practice, particles appearing as individual particles (i.e., falling and depositing independently, cf. Fig. 5A) can be distinguished from particles being part of aggregates (i.e., falling and depositing in groups of particles). However, as cores of PC2/PC3 aggregates can separate from the rest of their aggregates upon impact with the ground^21^, the distinction between individual particles > 63 µm and cores of PC2/PC3 aggregates (Fig. 5B) is ambiguous. PC1/PC3 aggregates are easily recognizable (Fig. 5C, D) as they form groups of relatively fine particles < 63 µm that are dispersed by the impact, but they are indistinguishable from each other as PC3 cores tend to rebound and get ejected away from the shell of fine particles upon impact. PC aggregates are identified in events A, B, and C, while they are absent in event D, in which AP1 and AP3 aggregates are identified (Fig. 5E-I).

For event A (Fig. 6a), the apparent size distribution of aggregates collected at UAS sampling altitude (i.e., 16 to 125 µm in Circle Equivalent Diameter, D_CE_) is smaller than that of aggregates collected at ground level (i.e., 63 to 500 µm). Particles inside aggregates are less abundant in the UAS sample (i.e., 7%) than at ground level (i.e., 22%). Individual ash particles < 63 µm between UAS (i.e., 13%) and ground (i.e., 18%) samples are slightly varying, while individual particles > 63 µm or cores of PC2/PC3 aggregates are more abundant in the UAS sample (i.e., 80%) than at ground level (i.e., 60%).

For event B (Fig. 6B), the apparent size distribution of aggregates collected at UAS sampling altitude (i.e., 125 to 1000 µm in D_CE_) is smaller than that of aggregates collected at the ground level (i.e., 250 to 2000 µm). Particles inside aggregates are less abundant in the UAS sample (i.e., 20%) than in the ground one (i.e., 83%). Conversely, UAS-sampled individual ash particles are more abundant (i.e., 44% for particles < 63 µm, and 36% for particles > 63 µm or cores of PC2/PC3 aggregates) than those sampled at ground level (i.e., 5% and 12%, respectively).

For event C (Fig. 6C), the apparent size distribution of aggregates collected at UAS flight altitude is smaller (i.e., 125 to 500 µm in D_CE_), than aggregates collected at ground level (i.e., 250 to 500 µm). Particles inside aggregates are less abundant in the UAS sample (i.e., 15%) than in that collected at the ground level (i.e., 52%). In contrast, individual ash particles < 63 µm are more abundant in the UAS sample (i.e., 71%) than in the ground one (i.e., 24%), while individual particles > 63 µm or cores of PC2/PC3 aggregates are slightly varying between UAS (i.e., 14%) and ground samples (i.e., 24%).

For event D (Fig. 6D), the apparent size distribution of UAS-collected aggregates is smaller (i.e., 63 to 1000 µm in D_CE_), than aggregates sampled at ground level (i.e., 250 to 4000 µm). Particles inside aggregates are less abundant in the UAS sample (i.e., 56%) than in the sample at ground level (i.e., 99%). Conversely, individual ash particles < 63 µm are almost only observed in the UAS sample (i.e., 43%), with an insignificant proportion in the ground one (i.e., 1%). The occurrence of individual particles > 63 µm or cores of AP1/AP3 aggregates is almost absent in both UAS (i.e., 1%) and ground (i.e., 0%) samples.

Finally, it is important to note that no secondary chemical compounds were detected on particle surfaces in any of the collected samples, based on Scanning Electron Microscope (SEM) analyses. This suggests that these particles preserved their primary chemical and physical characteristics during atmospheric transport and sampling, without significant post-emission alteration and chemical precipitation.

### Numerical modeling of particle trajectories

In addition to the remote and in-situ field data, and associated sample analyses, numerical modeling of particle trajectories was conducted using LagTrack^35^ based on the model of^36^ to evaluate the possible paths of particles falling from the volcanic cloud to the ground, including their passage through the UAS sampling locations. Particle sedimentation durations are also modeled to compare with the real sampling timing performed in the field (cf. supplementary material Data_S7 for model information). More specifically, and for each event, both individual particles and observed aggregate types were modeled within the size range measured in the collected samples, using averaged densities derived from^21,30^ and morphologies derived from the collected samples. In this model, a backward trajectory framework was deliberately adopted to limit uncertainties related to plume dynamics, as forward simulations would require defining the initial position of particles, which are poorly constrained. The backward approach allows us to bypass this major source of uncertainty by starting from well-constrained ground deposition locations. Additional uncertainties remain, notably those associated with atmospheric forcing: the simulations rely on ERA5 reanalysis data^38^, which provide spatially and temporally averaged atmospheric conditions and cannot fully capture local-scale wind variability in the field. Consequently, the trajectories should be viewed as idealized approximations of likely transport pathways rather than unique and exact reconstructions and deterministic reconstructions. Also, sensitivity tests spanning particle density and morphology from realistic ranges did not lead to significant changes in the results. This indicates that the model outcomes are primarily controlled by the atmospheric input data, rather than by particle property assumptions. Implementing a full uncertainty-propagation framework would therefore add substantial complexity while providing limited additional insight within the scope of the present study, which intentionally focuses on a simplified, first-order approach.

The results provide constraints on grain size ranges that are dynamically consistent with sedimentation from the volcanic plumes and associated clouds (considered as the particle release zones), versus those that show inconsistent sedimentation paths (hence likely influenced by other mechanisms such as SDGIs that are not considered by the model). Modeled RH values as a function of altitude are also retrieved from ERA5 dataset^38^ to evaluate the atmospheric conditions of the studied fallout.

For event A (Fig. 7A), only individual particles or PC2 aggregates > 125 µm show coherent backward trajectories connecting the sampling sites to the plume or cloud, as well as PC1/PC3 aggregates > 250 µm. Note that the UAS operated within the area intersected by these particle trajectories. Also, the modeled settling duration range for the observed particles between the UAS and the ground is estimated between 2 and 5 min for individual particles or PC2 aggregates, and ca. 5 min for PC1/PC3 aggregates, which corresponds to the sampling delay between the UAS sampling window and the ground sampling window applied in the field (Fig. 6), meaning that both sampling may catch the same sedimentation streak, as confirmed by the model. Individual particles or PC2 aggregates < 125 µm follow low-altitude paths, inconsistent with a direct plume or cloud origin. Similarly, PC1/PC3 aggregates smaller than 250 µm appear to originate from lower atmospheric layers, thus cannot be explained by individual sedimentation only. For this event, the RH profile between 0 and 4 km a.s.l., centered on the Sakurajima Peninsula, shows values between 15 and 80%. These values indicate that the lower atmosphere was not saturated with water vapor (i.e., RH < 100% and no rain formation), in agreement with the optical observations (Fig. 2A). They also suggest that the atmosphere below the volcanic cloud was slightly moist, with RH values < 60%, consistent with the RH values recorded by the UAS (Fig. 4A).

For event B (Fig. 7B), the results mirror those of event A, with coherent backward trajectories observed only for individual particles or PC2 aggregates > 125 µm, as well as PC1/PC3 aggregates > 250 µm. Note that the UAS operated within the area intersected by these particle trajectories. Also, the modeled settling duration range for the observed particles between the UAS and the ground is estimated < 3 min for both individual particles and aggregates, which encompasses the sampling delay applied in the field (Fig. 6). Smaller individual particles and aggregates display low-altitude paths, suggesting that they cannot originate from direct and individual sedimentation from the eruption plume and cloud. For this event, the RH profile between 0 and 4 km a.s.l., centered on the Sakurajima Peninsula, shows values between 5 and 95%. These values indicate that the lower atmosphere was not saturated with water vapor, in agreement with the optical observations (Fig. 2B). They also suggest that the atmosphere below the volcanic cloud was slightly to relatively moist, with RH values < 70%, consistent with the RH values recorded by the UAS (Fig. 4B).

For event C (Fig. 7C), only individual particles or PC2 aggregates > 250 µm exhibit plausible backward trajectories linking sampling locations to the plume and cloud. For PC1/PC3 aggregates, only those > 500 µm are consistent with a direct sedimentation path. Note that the UAS operated within the area intersected by these particle trajectories. Also, the modeled settling duration range for the observed particles between the UAS and the ground is estimated between 4 and 8 min for individual particles and between 4 and 10 min for PC1/PC3 aggregates, which encompasses the sampling delay applied in the field (Fig. 6). Smaller individual particles and aggregates show evidence of a lower-altitude origin. For this event, the RH profile between 0 and 4 km a.s.l., centered on the Sakurajima Peninsula, shows values between 10 and 85%. These values indicate that the lower atmosphere was not saturated with water vapor, in agreement with the optical observations (Fig. 2C). They also suggest that the atmosphere below the volcanic cloud was slightly moist to humid (humid air is typically defined above 80 and below 100% of RH), with RH values < 85%, consistent with the RH values recorded by the UAS (Fig. 4C).

For event D (Fig. 7D), coherent paths are observed for individual particles or PC2 aggregates > 125 µm. For AP1/AP3 aggregates, only those > 250 µm show coherent transport pathways from the cloud to the ground. Note that the UAS was located west of the particle trajectories but still within the area of sedimentation. Also, the modeled settling duration range for the observed particles between the UAS and the ground is estimated < 2 min for both individual particles and aggregates, which encompasses the sampling delay applied in the field (Fig. 6). Smaller individual particles and aggregates again show evidence of a lower-altitude origin. For this event, the RH profile between 0 and 4 km a.s.l., centered on the Sakurajima Peninsula, shows values between 60 and 100%. These values indicate that the atmosphere above 2 km a.s.l. was saturated with water vapor (i.e., rain formation), in agreement with the optical observations (Fig. 2D). They also suggest that the atmosphere below the volcanic cloud was relatively moist to humid, with RH values < 80%, and was experiencing rain fallout, consistent with the RH values recorded by the UAS (Fig. 4D).

## Discussion

### Conditions for the formation of volcanic ash aggregates

This study demonstrates that volcanic ash aggregation can occur even during low-intensity volcanic events (Fig. 2), including ash-venting (events A, B, and D), as well as mild Vulcanian explosions (event C), both producing low-altitude plumes and clouds (i.e., < 2 km a.s.l.).

Two distinct aggregation mechanisms are involved, each associated with specific atmospheric conditions and aggregate types. Under dry, moist, or humid but unsaturated conditions (i.e., RH < 100%, events A, B, and C, cf. Figs. 2A–C, 4A–C, and 7A–C), particle aggregation is mainly driven by electrostatic forces, as evidenced by the occurrence of electrically charge fallout particles (Fig. 3A–C) and the presence of loosely bound PC aggregates (Fig. 5A–D), known to form because of these electrostatic forces^20,21,24^. Under water-saturated conditions (i.e., occurrence of rain, event D, cf. Figs. 2D, 4D, and 7D), our results highlight the critical role of liquid water in enhancing particle aggregation efficiency and in the formation of APs (Fig. 5E–I). This combination of conditions likely facilitated cohesive interactions among ash particles through liquid bridging, promoting the growth of stable aggregates^5,10,39^, as also shown by the disdrometer data (cf. blue dots in Fig. 3D). Conversely, eruptions exhibiting elevated relative humidity but lacking liquid water did not produce APs, indicating that vapor-phase moisture alone is insufficient to initiate AP formation, in agreement with the conclusions of^11^.

Backward particle trajectory modeling reveals that only relatively coarse ash particles (> 125 to 500 µm, depending on the studied event) sedimented as individual particles (Fig. 7). Conversely, sedimentation of smaller particles (< 125 to 500 µm depending on the studied events) cannot be modeled as individual-particle sedimentation. Hence, the coarsest particles that are found in the collected samples (Fig. 5B) have deposited individually, at least during dry conditions. This observation is confirmed by the disdrometer data (cf., orange data in Fig. 3). In contrast, smaller particles that are found in the collected samples (Fig. 5) must have been deposited through collective sedimentation processes such as aggregation and SDGIs (cf. the two following sections).

### Dynamic evolution of aggregation processes during tephra fallout

To enable a valid comparison between ground and UAS samples (Fig. 6), backward particle trajectories were modeled (Fig. 7) to verify whether the observed particles intersected the UAS flight paths, and whether their sedimentation times matched the delay between UAS and ground sampling. This ensures that both datasets represent the same fallout streak. Also note that UAS-based particle sampling is not affected by drone-generated turbulence, as the sampler is mounted on top of the drone, where turbulence has been shown to be negligible^40–42^.

The particle trajectory models confirm that particles between 250 and 1000 µm (including both individual particles and aggregates, depending on the event) passed through both the UAS and ground sampling areas (Fig. 7). Model simulations also indicate sedimentation times from the UAS positions to the ground ranging from 2 to 10 minutes, consistent with the sampling dlay applied in the field between the UAS and the ground (Fig. 6). Together, these results validate the comparison between ground and UAS samples for the observed coarse ash particles. In contrast, the sedimentation of finer particles (< 125 to 250, depending on the event) cannot be explained by simple individual settling, but most likely with collective settling such as particle aggregation and/or SDGIs (cf. following section).

Grain size distribution data reveal that aggregation intensified sharply within the last 500 vertical meters of fallout. Indeed, the fraction of particles found as aggregates increased from 7, 20, 15, and 56% in UAS samples to 22, 83, 52, and 99% in ground samples for events A, B, C, and D, respectively (see brown data in Fig. 6). Disdrometer data (at ground level) show the same trend, with minimum aggregation rates of 17, 67, 23, and 100% for the same events (see brown dots in Fig. 3).

In addition, modeling of aggregates in the observed size range (between 16 and 4000 µm) suggests that only the larger ones (> 250 to 500 µm, depending on the event) follow the modeled sedimentation trajectories (Fig. 7). However, smaller aggregates were also observed in both UAS and ground samples (green histograms in Fig. 6), although they are not predicted by the model. This discrepancy suggests that these small aggregates formed below the volcanic cloud during sedimentation. Also, it is important to note that aggregate sizes in collected samples (Fig. 6) are overestimated due to breakage and spreading upon impact on the thin sections (Fig. 5). Hence, the original aggregate sizes before impacts are relatively lower than the quantified aggregate sizes after impact, which is also in agreement with the disdrometer data that indicate maximum aggregate sizes of 400, 600, 900, and 1300 µm for events A, B, C, and D, respectively (Fig. 3). These latter values are indeed consistent with independent measurements at Sakurajima using high-speed cameras and computed tomography measurements on unbroken aggregates^12,21^.Overall, the comparison between UAS and ground samples supports a scenario in which aggregation is not limited to the volcanic cloud but substantially develops throughout the final 500 meters of sedimentation. This is evidenced by the larger aggregate sizes and higher aggregate fractions in ground samples (Fig. 6) compared to the UAS ones, and endorsed by disdrometer measurements (Fig. 3) and particle trajectory modeling (Fig. 7). The atmospheric transport pathway of volcanic particles can be summarized as follows (Fig. 8): initial emission (not constrained due to lack of direct observations), (1) cloud formation and dispersion with incipient aggregation (as described for the same UAS dataset by^34^), (2) sedimentation, during which aggregation develops further, and (3) final deposition, where aggregation reaches maturity.

### Fine ash sedimentation

Despite their low individual settling velocities, fine volcanic ash particles (< 63 µm) are frequently observed as single particles in UAS samples and, to a lesser extent, in ground deposits (Figs. 5 and 6). These fine ash particles are progressively incorporated into the aggregates during fallout, and some of them remain individual until they settle on the ground. The presence of these individual particles in the collected samples cannot be explained by individual sedimentation alone (Fig. 7). This observation suggests the involvement in other sedimentation mechanisms, which are discussed hereafter.

SDGIs involve the formation of ash-rich fingers (sometimes referred to as streak fallouts) that descend rapidly from volcanic clouds through the atmosphere, enhancing the downward transport of fine ash despite strong drag forces^8,43,44^. SDGIs are triggered when local particle concentrations exceed a critical threshold, creating density instabilities that propagate downward, even in dilute clouds^45–47^. Although such finger-like structures were rarely detected with the visible-wavelength imagery in our videos (Fig. 2B), this may be due to low mass loading resulting in low particle concentrations, which hinder visual detection. However, similar SDGI structures have been optically observed and measured in-situ^48^ at Sakurajima during more intense ash emissions. OPC data reveal stratified vertical layers enriched in 0.3 to 40 µm particles beneath the volcanic clouds (Fig. 4). These anomalies, which last from ca. 20 s to 1 min in dry conditions, offer strong in-situ evidence for such a process, as these discrete, particle-rich layers are inconsistent with individual settling trajectories or simple atmospheric diffusion (Fig. 7). More concentrated and longer anomalies are observed under wet conditions, which may correspond to water micro-droplets typically within the OPC measurement range^49^. These droplets can host and agglomerate fine volcanic particles, ultimately leading to the formation of AP3s during their fallout^39^. In parallel, disdrometer data detecting relatively coarser particles at ground levels show significant variations in particle sedimentation rate, these variations also being visible in minute-scale durations (Fig. 3). Importantly, SDGIs and aggregation are not mutually exclusive: SDGIs may efficiently transport fine ash in the early phases of the eruptions, while aggregation can further increase sedimentation efficiency later on^8,43,44^.

Other mechanisms, such as turbulent clustering, sedimentation waves, atmospheric remnants, and aggregate disintegration, can also influence fine ash sedimentation in volcanic clouds. However, in the specific case studies presented here, their role appears to be limited compared to the combined effects of SDGIs and aggregation. (i) Turbulent clustering arises from particle–turbulence interactions that cause inertial particles to preferentially concentrate in certain regions of a turbulent flow^50–52^. Such processes could, in principle, contribute to the localized enrichment of fine particles observed in OPC profiles (Fig. 4). Yet, turbulent clustering is typically associated with more vigorous or sustained currents than those produced by the weak ash emissions documented in this study, making it an unlikely dominant mechanism here. (ii) Sedimentation waves, generated by pulsed emissions or source variability, can produce quasi-periodic vertical structures that enhance the fallout efficiency of fine ash^53^. Given the short-lived and low-intensity nature of the investigated ash emissions (Fig. 2), this process can reasonably be excluded. (iii) Atmospheric remnants of earlier eruptive phases may also contribute fine particles to the observed fallout. At a persistently active volcano such as Sakurajima, fine ash emitted during previous pulses can remain suspended for extended periods and later settle together with more recent material. While this mechanism may increase the overall fine ash content, it cannot account for the sharply stratified layers detected by the OPC (Fig. 4), nor for the efficient sedimentation observed where individual settling alone is implausible. (iv) Finally, aggregate disintegration can release fine particles that subsequently settle independently^10,19,54^. Nevertheless, the significant number of individual fine particles captured at higher altitudes by UAS sampling cannot be explained by this mechanism, since aggregate fragmentation generally occurs upon impact at ground level.

## Conclusions

This study presents the first UAS investigation of volcanic ash aggregation during the sedimentation from volcanic clouds. Thanks to the unique natural laboratory offered by Sakurajima volcano, this study captured new insights into ash sedimentation processes through a combination of UAS- and ground-based measurement techniques, as well as laboratory measurements of collected samples and particle trajectory modeling. Key aspects are highlighted by this multidisciplinary study:

- Volcanic ash aggregation can occur with low mass eruption rates (Fig. 2) and particle concentrations (Figs. 3 and 4), such as during weak Vulcanian explosions and even ash-venting, and does not necessarily require the high concentrations typical of large explosive eruptions.
- Ground- and UAS-based data comparison (Figs. 5 and 6) reveals that while volcanic ash aggregation is already observed above ground (i.e., in or below volcanic clouds), it significantly intensifies during the final stages of fallout (i.e., last 500 m above ground). These findings are consistent with previous isolated in-cloud observations suggesting incipient aggregation within volcanic clouds, and highlight the major role of syn-sedimentation aggregation.
- Field observations show that aggregation is promoted by polydisperse grain size distributions, favoring particle collisions between coarse (up to 1 mm) and fine (down to 0.3 µm) particles, which have different settling velocities. Coarse particles predominantly settle individually or as PC2s (evidenced by the collected samples and numerical models; Figs. 5, 6, and 7), while fine particles must settle collectively (evidenced by the collected samples, OPC measurements, and numerical models; Figs. 4, 5, 6, and 7). This study also confirms how particle sticking is facilitated by electrostatic forces in dry conditions, leading to the formation of PCs (evidenced by the collected samples, and particle charge measurements; Fig. 3), while in wet conditions water condensation is even more efficient in aggregating particles, promoting the formation of APs (evidenced by the collected samples, disdrometer, and atmospheric measurements; Figs. 3, 4, and 7). Although chemical binding may enhance aggregation in some contexts, no evidence of such processes was detected in the collected samples.

This study emphasizes the necessity of direct field, in-situ, and airborne observations to complement laboratory experiments and numerical models. Field data provide essential insights into the dynamics and efficiency of ash aggregation and collective sedimentation, which are critical for improving volcanic ash dispersal forecasts and hazard assessment models. Overall, the insights gained from this study lay important groundwork for refining models of tephra transport, atmospheric residence times, and ash deposition patterns. However, several aspects remain to be addressed. Future work should aim to better constrain the initial conditions at the time of fragmentation, extend observations to eruptions of different styles and intensities, and investigate aggregation processes at higher altitudes directly within volcanic clouds, where data remain scarce. In addition, quantifying the efficiency and frequency of collective particle settling across eruption types will be essential. Finally, the development of sampling methods capable of preserving fragile airborne aggregate structures (e.g., resin embedding rather than adhesive carbon tape) would greatly improve our ability to characterize these processes.

## Materials and methods

### Visible-wavelength imaging and parameterization of volcanic plumes and clouds

The visible-wavelength videos were captured using a Canon Legria HF G40 and a Canon Legria HF G50, both operating at a frame rate of 50 Hz, a resolution of 1920 × 1080 pixels, and with adjustable Field Of Views (FOVs). These ground-based cameras were set to record continuously and were always placed together at a chosen location (Fig. 1D), but with different FOVs to capture both plume dynamics and sedimentation processes. The camera locations varied throughout the campaign (Fig. 1C), mainly to align with prevailing wind directions. These placements allowed volcanic clouds to be captured from viewing angles as close to perpendicular as possible. The camera orientation (i.e., bearing of their Line Of Sight (LOS) from North), inclination (angle of their LOS from the horizontal), and auxiliary measures for determining the FOV, were recorded at the time of each setup.

Following the approaches of^28,55^, the maximum heights of each pulse were inferred as a function of time (Fig. 2). A correction to account for the wind effect was also applied, which is detailed in^27^. The wind direction was extracted from the ERA5 hourly data on pressure levels^38^ (https://cds.climate.copernicus.eu/stac-browser/collections/reanalysis-era5-single-levels?.language=en) (also cf. supplementary material, Data_S1, direct data file used available at: https://gofile.me/7ACTk/fBYxJGIQB). Following these procedures, the plume heights were automatically calculated by taking the crater rim as the vent position. However, this assumption may result in significant errors for Sakurajima volcano, as the Minamidake crater rim, depending on the observation position, can be up to 300 m higher than the actual vent locations^56^. To correct this bias, the difference between the height of the crater rim (previously taken as a reference) and the estimated vent height (750 m a.s.l.) from^56^ was added to the measured plume heights.

### Geophysical data to estimate ash discharge rates at vent

Ash discharge rates were estimated following the approach developed by^29^. This method combines ground deformation measurements and seismic tremor analysis provided by the Sakurajima Volcano Observatory (SVO) (data available at https://gofile.me/7ACTk/AyvGQ18XR) (also cf. supplementary material, Data_S1, direct data file used available at: https://gofile.me/7ACTk/fBYxJGIQB). Ground deformation associated with Vulcanian eruptions was analyzed using a Mogi model to estimate pressure source volume changes, which strongly correlate with the emitted ash mass (as measured in the field by a disdrometer network) through an empirical ratio. The amplitude of volcanic tremor signals in the 2–3 Hz frequency band was also used, as it similarly shows a strong correlation with the emitted ash mass. The total ash discharge mass was then calculated through a linear empirical model combining both contributions, expressed as:

1 $${\mathrm{W}} = \alpha {\mathrm{A}} + \beta {\mathrm{V}} + \gamma$$

where W is the estimated ash mass, A is the summed seismic amplitude, and V the summed pressure source volume change. α, β, and γ are empirically derived coefficients optimized to maximize the correlation between the field-measured and calculated ash masses. Ash discharge rates were finally obtained by applying this model over the selected time resolution (Fig. 2).

### Disdrometers to estimate coarse particle (> 150 µm) sedimentation rate, size, and settling velocity at ground level

The mobile disdrometers used in this field campaign were the Thies Clima^TM^ Laser Precipitation Monitors (LPMs), which are optical disdrometers (Fig. 1D) that were originally developed to detect liquid and solid hydrometeors but were recently applied for tephra fallout quantification and differentiation based on their size and velocity signatures^30^. The LPMs were set up in two different sites to cover two main cloud dispersion axes (Fig. 1C). The LPMs particle size measurement range is 150 to 8000 µm, with particle settling velocities between 0 and 10 m s^−1^. Based on the raw data acquired by the LPMs (i.e., time, as well as particle sizes, numbers, and velocities), particle sedimentation rates can be computed (cf. supplementary material, Data_S2).

We also set up useful data filters to separate dense invalid data from individual particles or PC2 aggregates and from less dense PC1/PC3 aggregates (Fig. 3A–C), based on measured particle density and shape characteristics, following the approach of^30^. This method uses a drag equation^57^ assuming a maximum particle density of 2700 kg m^−3^ and a maximum particle sphericity of 1, which corresponds to dense, compact particles. This setup defined a margin trend of particle size and velocity, above which particles were considered noncoherent (due to physically unrealistic particle densities or sphericities). Additionally, to discriminate individual particles or dense PC2 aggregates with less dense PC1/PC3 aggregates, the approach introduced an individual particle trend, based on a lower density limit (2150 kg m^−3^) and a sphericity of 0.55, corresponding to the 90th percentile of the measured particle shapes. Particles falling above this trend display similar signatures of the individual particles or PC2 aggregates, while particles falling below this trend display similar velocity and size signatures of the PC1/PC3 aggregates observed by high-speed camera measurements^11^. Data acquired during wet conditions and the occurrence of rain (Fig. 3D) is also compared to the empirical law described for pure water droplets^37^.

### Measurements of the electrical charge of falling particles at ground level

The electrical charges carried by volcanic ash particles were measured at ground level with an Arduino-based device designed at the University of Geneva^31^ for continuous analysis of the electrical charge of falling particles (Fig. 1D). This instrument is inspired by laboratory setups^58,59^, and is constituted of a brass Through-Type Faraday Cage (TTFC) connected to an amplifier circuit and an Arduino for data acquisition. Charged material crossing the TTFC induces an electronic flow on the inner wall of the cage. This produces a very low voltage upon entrance of the material in the TTFC and a symmetrical signal upon exit from the TTFC. After passing through the amplifier circuit, the voltage (V) outputed to the Arduino (with quantified analytical errors) becomes inversely proportional to the bulk electrical charge (Q) of the material (Fig. 3A–C) within the TTFC such as:

2 $${\mathrm{Q}} = - {\mathrm{V}} \times {\mathrm{Cf}}$$

with Cf the reference capacitance of the condenser in the amplifier circuit (cf. supplementary material, Data_S2).

### Ground-based sampling of falling particles

Falling particles were collected using thin section glasses coated with adhesive carbon tape (Figs. 1D and 5), which were simply laid on the ground following the sampling approaches described in^11,21,33^. Special care was taken to position the thin sections in areas protected from remobilized dust and ash, and close to the disdrometer and the electrical charge measurement device.

### Unoccupied Aerial System (UAS)-based imaging, measurements and sampling

The Unoccupied Aerial System (UAS) used in this study (AeroVolc) is detailed in^32^. For field operations, the DJI^TM^ Matrice 30 quadcopter (Fig. 1D) was chosen for its robustness and operational efficiency in harsh volcanic settings. Weighing under 4 kg, the platform is engineered to withstand environmental stressors such as fine ash particles, precipitation, and strong winds. The drone is equipped with a sophisticated multi-camera array, including first-person-view, wide-angle, and zoom lenses, facilitating precise navigation and multi-directional imaging in complex terrains. Typical flight durations, when carrying scientific payloads, do not exceed 30 minutes (cf. supplementary material, Data_S3 for flight details). The drone is rated for a recommended payload of up to 299 g, allowing the deployment of compact yet advanced sampling and sensor systems (Fig. 1D). Critically, prior aerodynamic analyses and computational fluid dynamics simulations have consistently demonstrated that turbulence associated with quadcopter rotors is concentrated directly be- neath the propellers, while airflow above and between the rotors remains relatively undisturbed^40–42^. This aerodynamic characteristic is particularly advantageous for air sampling missions, ensuring that measurements taken above the rotor plane are minimally influenced by drone-induced disturbances, including volcanic ash aggregate sampling.

In this work, data were obtained using a custom-built payload named the Airborne Ash Collector and Counter (A_2_C_2_), also described in detail in^32^ (Fig. 1D). This system integrates an Alphasense^TM^ N3 optical particle counter (OPC) to provide in-situ atmospheric data (temperature, RH, and pressure), as well as in-situ airborne granulometric data for solid particles ranging from 0.3 to 40 µm in diameter (cf. supplementary material, Data_S4), hence ignoring larger particles. The OPC operates by detecting light scattered by individual particles as they traverse a laser beam within a controlled airflow. The instrument is factory-calibrated with reference particles of known size and refractive index, offering reliable and reproducible measurements^60^. Particle mass concentrations, specifically PM_1_, PM_2.5_, and PM_10_, are derived from the raw number concentration data. This conversion assumes a uniform particle density of 2700 kg m^−3^^21^ and treats the optical equivalent diameter as a proxy for the geometric diameter (Fig. 4). In addition to the OPC, the A_2_C_2_ includes a remotely actuated particle sampling module, which contains sample holders equipped with thin sections similar to those deployed in ground-based sampling stations (Fig. 5).

### Optical microscopy, scanning electron microscopy (SEM), and image analysis

Collected samples from thin sections coated with carbon tape were first imaged with a Keyence^TM^ VHX-7000 optical microscope, which enabled the acquisition of multi-focused (z-stacking technique) high-definition images, allowing the global pictures of each sample and the selection of representative sample areas to image at the SEM. Samples were then coated with a thin carbon layer to improve conductivity and prevent charging during imaging at the SEM. Samples were analyzed using a JEOL^TM^ JSM-7001F Field Emission Scanning Electron Microscope (FE-SEM). High-resolution images were acquired at 10 kV, with a working distance of 10 mm in secondary electron mode, as well as at different magnifications ranging from ×40 to ×20,000 to capture both the overall particle assemblages and fine-scale surface textures. Special attention was given to ensure that imaging was representative across all grain size fractions. Energy Dispersive X-ray Spectroscopy (EDS, with JEOL JED-2300 sensor) was used to qualitatively check for chemical composition and possible post-depositional surface alterations (cf. supplementary material, Data_S5 for all raw and segmented images).

An image nesting strategy was adopted for the image analysis. Multiple images at increasing magnifications were systematically acquired to cover the full grain size range while maintaining statistical representativeness. This approach follows a two-dimensional adaptation of the nested methodology described in^34^ for vesicles inside volcanic rocks. Particle componentry was manually classified by visually inspecting the SEM images. Particles were categorized based on whether they appeared as isolated individual grains or were incorporated into aggregates. Grain size distributions were reconstructed using a semi-automated workflow (cf. supplementary material, Data_S6). After image binarization, particle outlines were extracted, and particle sizes were calculated based on the D_CE_, which is defined as the diameter of a circle having the same projected area as each analyzed particle. Raw data from image analysis were processed using the Matlab-based code FOAM^34^ to build grain size distributions from 1 to 4 mm (Fig. 6).

### Numerical modeling of falling particle trajectories

Particle trajectories were modeled using LagTrack^35^, which is a Matlab-based Lagrangian particle tracking code that applies the method of^36^ to calculate the drag of non-spherical particles across a wide range of Reynolds numbers (10^-4^ to 10^7^) (also cf. supplementary material Data_S7) (code available at: https://github.com/e5k/LagTrack). Particle trajectories were simulated starting from the ground site locations using the backward mode until they reached the maximum plume heights. The DEM of the Sakurajima area was downloaded from the SRTM dataset (30 m resolution)^61^ (https://portal.opentopography.org/datasetMetadata?otCollectionID=OT.042013.4326.1, direct data file used available at: https://gofile.me/7ACTk/fBYxJGIQB). As particles are also initialized in time and their initial motion is defined by the local wind field, reanalyzed atmospheric data were downloaded for the selected days from the ERA5 hourly data^38^. This dataset includes the East-, North- (horizontal space resolution of 0.25°), and vertical components (based on pressure levels) of wind speed, as well as other needed atmospheric parameters (e.g., air density and viscosity). The time step for the trajectory calculations was set at 0.1 s (given the ratio between the maximum wind speed and the atmospheric data resolution), and the atmospheric dataset was linearly interpolated in time and space at each step. Each particle was characterized by its equivalent diameter (D_CE_), density (ρ), flatness (f), and elongation (e), following the definitions of^62^. Modeled D_CE_ span the size range observed in each collected sample. ρ is set at 2700 kg m^−3^ for individual particles and PC2 aggregates, while it is set at and 760 kg m^−3^ for PC1/PC3 aggregates, following the density values provided by^21,30^. Note that^12^ highlights slightly different density values (between 2900 and 3800 kg m^−3^ for individual particles and PC2 aggregates and between 1200 and 3200 for PC1/PC3 kg m^−3^ aggregates). These differences may lead to minor variations in absolute particle trajectory modeling, but the relative trends and interpretations remain unchanged, as shown by sensitivity tests. Considering that measured particles by the disdrometer follow the trend of micro-water droplets^37^, ρ is set at 1000 kg m^−3^ for the AP1/AP3 aggregates. Considering the collected samples, f and e were set at 0.8 for both individual particles and PC aggregates. As water micro-droplets have pseudo-spherical shapes in the modeled size range^63^, f and e were set at 1 for AP aggregates. Note that decision-making during the campaign was supported by tailored Numerical Weather Prediction (NWP) and transport simulations using the Weather Research and Forecasting (WRF)^64^ (https://www2.mmm.ucar.edu/wrf/users/download/free_data.html, direct data file used available at: https://gofile.me/7ACTk/fBYxJGIQB) and FALL3D models^65^, as described in^35^.

## Supplementary Information

Supplementary Information 1: Raw data of the plume altitudes analysis (Events A, B, and C), as well as raw data of the ash discharge rates analysis (Events A, B, C, and D).

Supplementary Information 2: Raw data acquired by the disdrometers (Events A, B, C, and D), as well as raw data acquired by the electrical charge measurement devices (Events A, B, and C).

Supplementary Information 3: Raw data of the Unoccupied Aerial System (UAS) (Events A, B, C, and D).

Supplementary Information 4: Raw data of the Optical Particle Counter (OPC) (Events A, B, and C, D).

Supplementary Information 5: Images of the collected samples and their segmented versions (Events A, B, C, and D).

Supplementary Information 6: Raw data of the grain size and grain componentry analysis (Events A, B, C, and D).

Supplementary Information 7.

Supplementary Information 8.

## Data Availability

All data are available in the main text or the supplementary materials. All samples analyzed in this study are stored and available at the Department of Earth Sciences, University of Geneva. The data is also available via the following public link: [https://gofile.me/7ACTk/CnyHxu38t]. All necessary permissions and licenses for the collection of falling particles, ash, and aggregates, as well as for conducting drone flights, were obtained.
