# Supplementary material for: In-situ evidence of volcanic ash aggregation during fallout from combined ground- and UAS-based observations
Source: Sci Rep. 2026 Mar 26;16:15083. doi: 10.1038/s41598-026-45460-x (PMC13171961; doi:10.1038/s41598-026-45460-x)

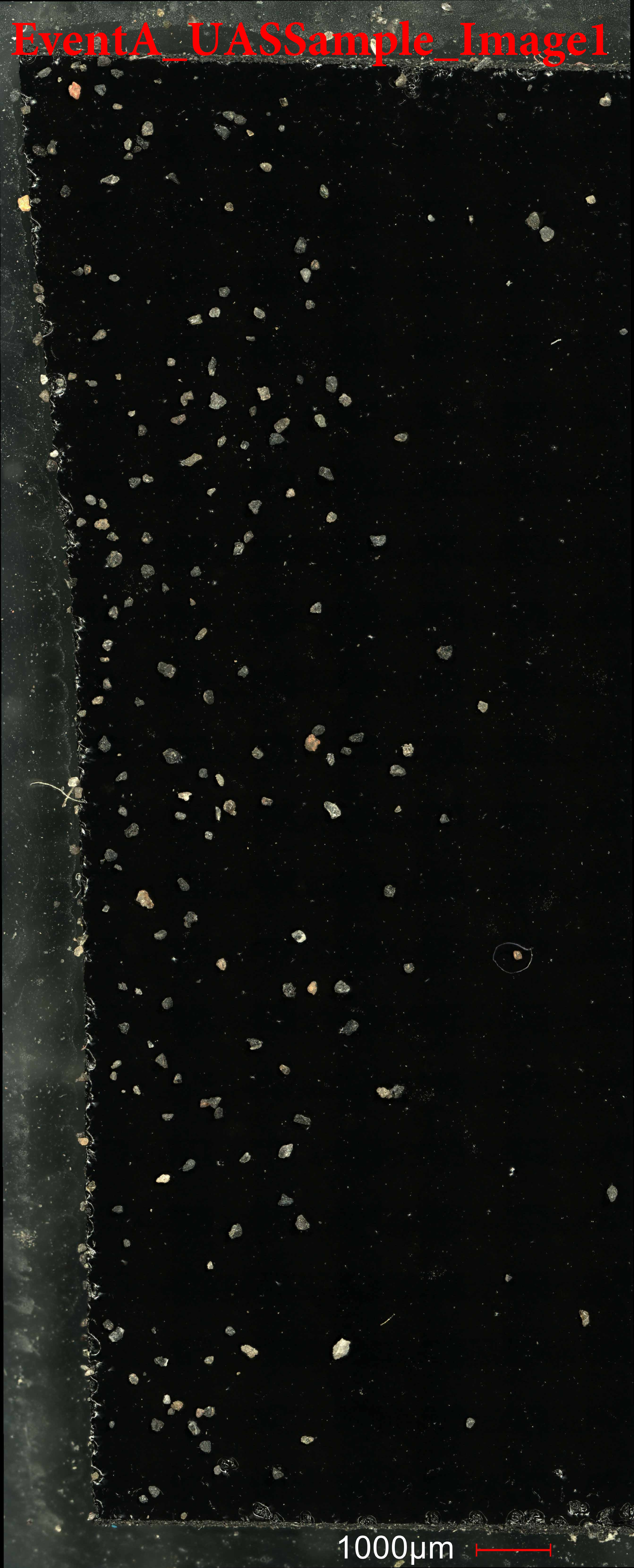
EventA_UASSample_Image1
1000µm

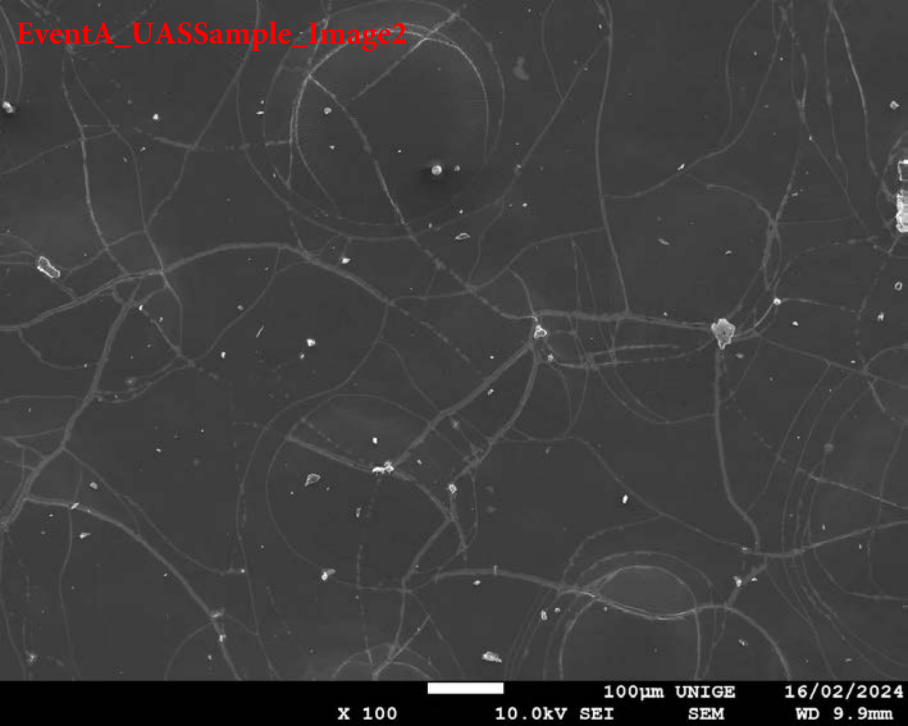
EventA_UASSample_Image2
100µm UNIGE
16/02/2024
X 100
10.0kV SEI
SEM
WD 9.9mm

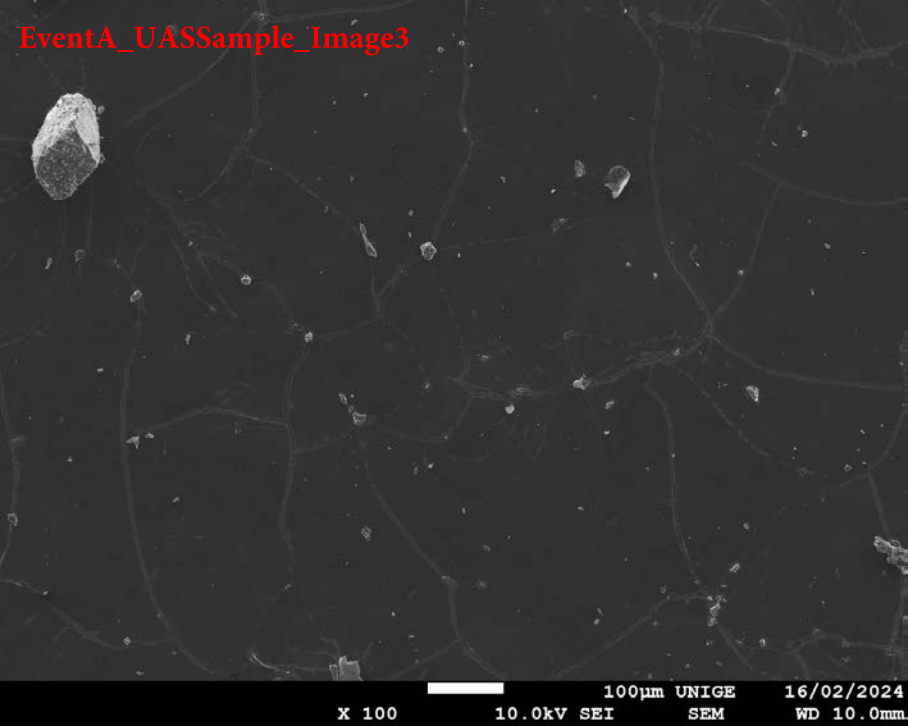
EventA_UASSample_Image3
100µm UNIGE
16/02/2024
X 100
10.0kV SEI
SEM
WD 10.0mm

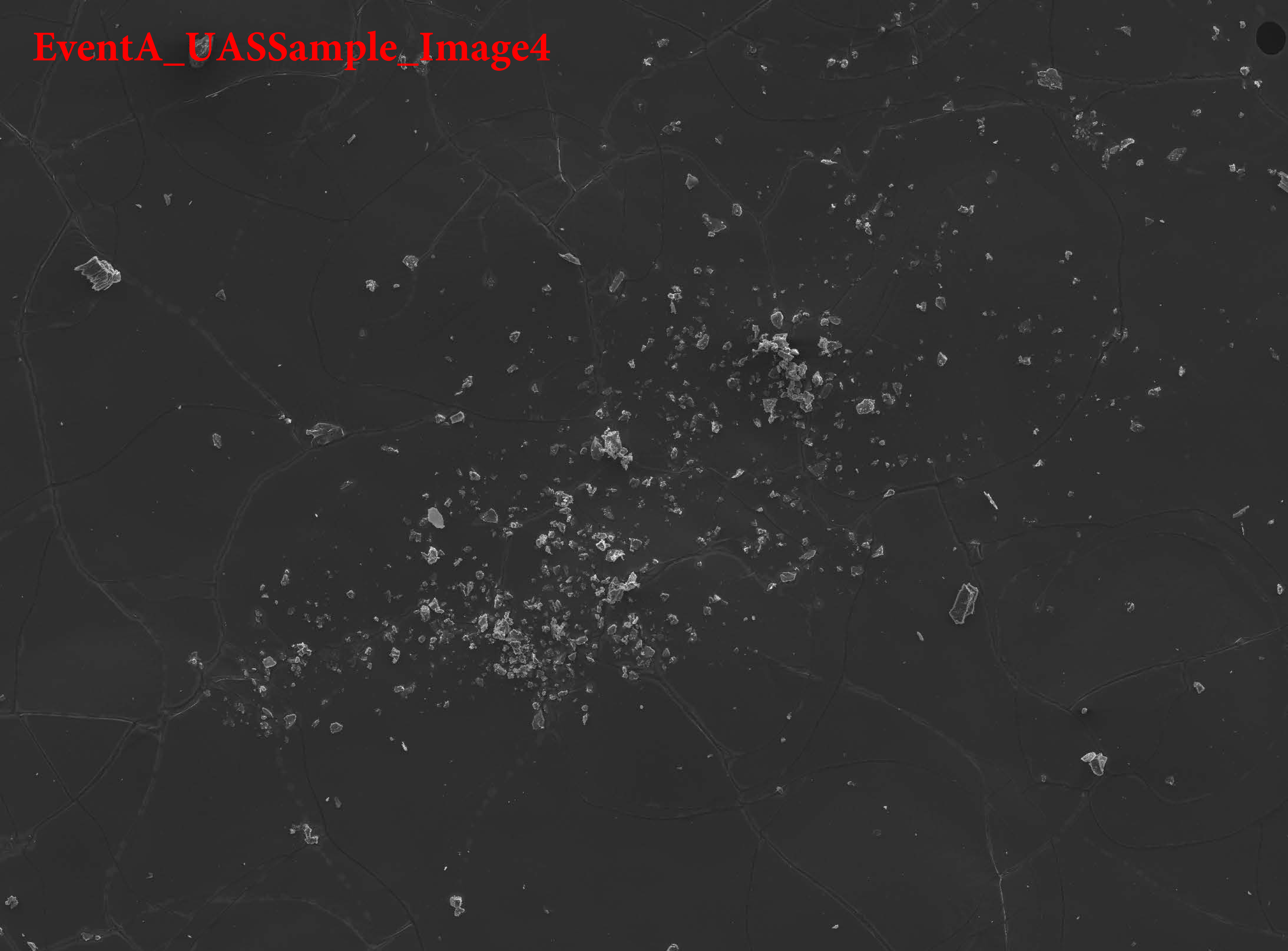
EventA_UASSample_Image4

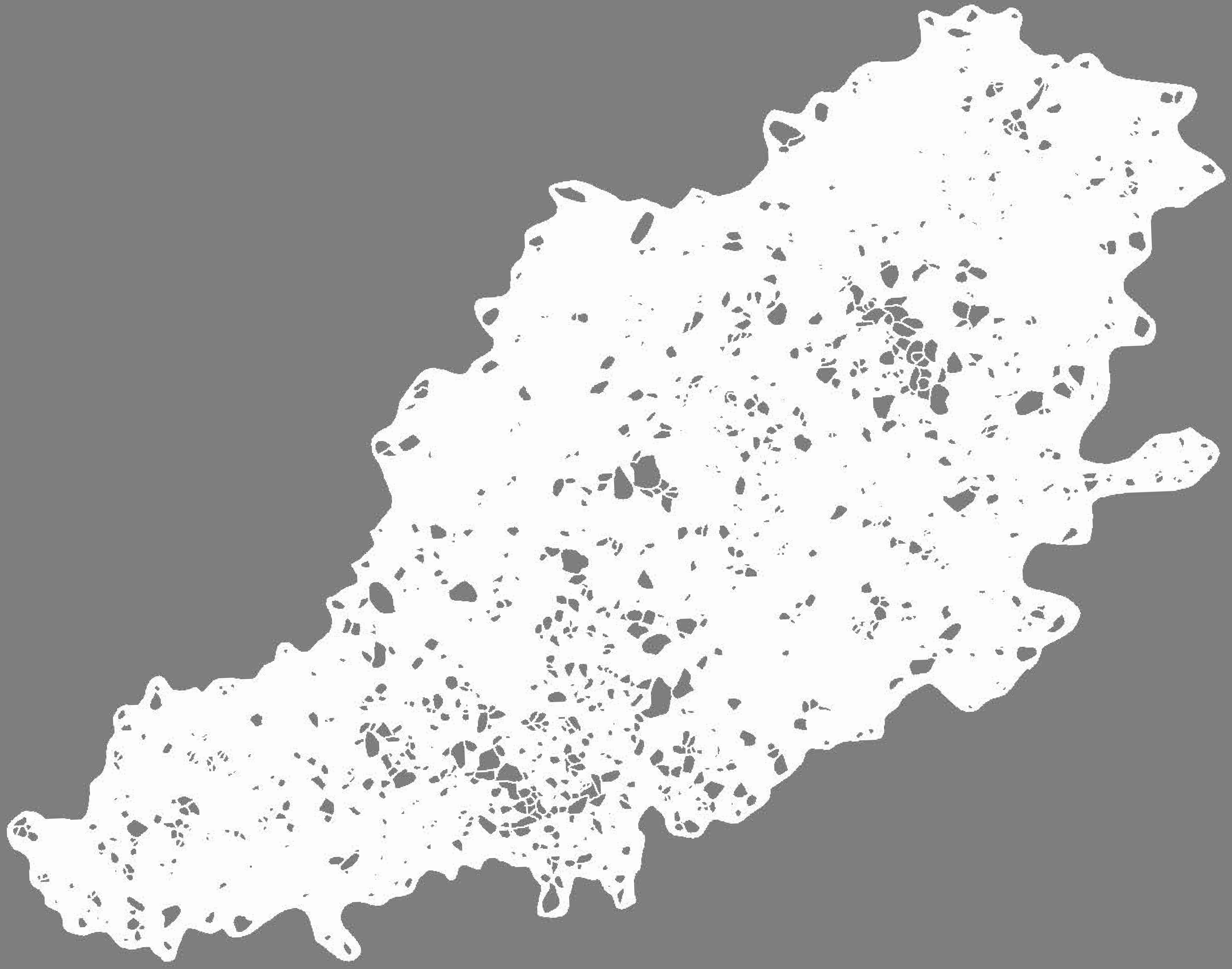

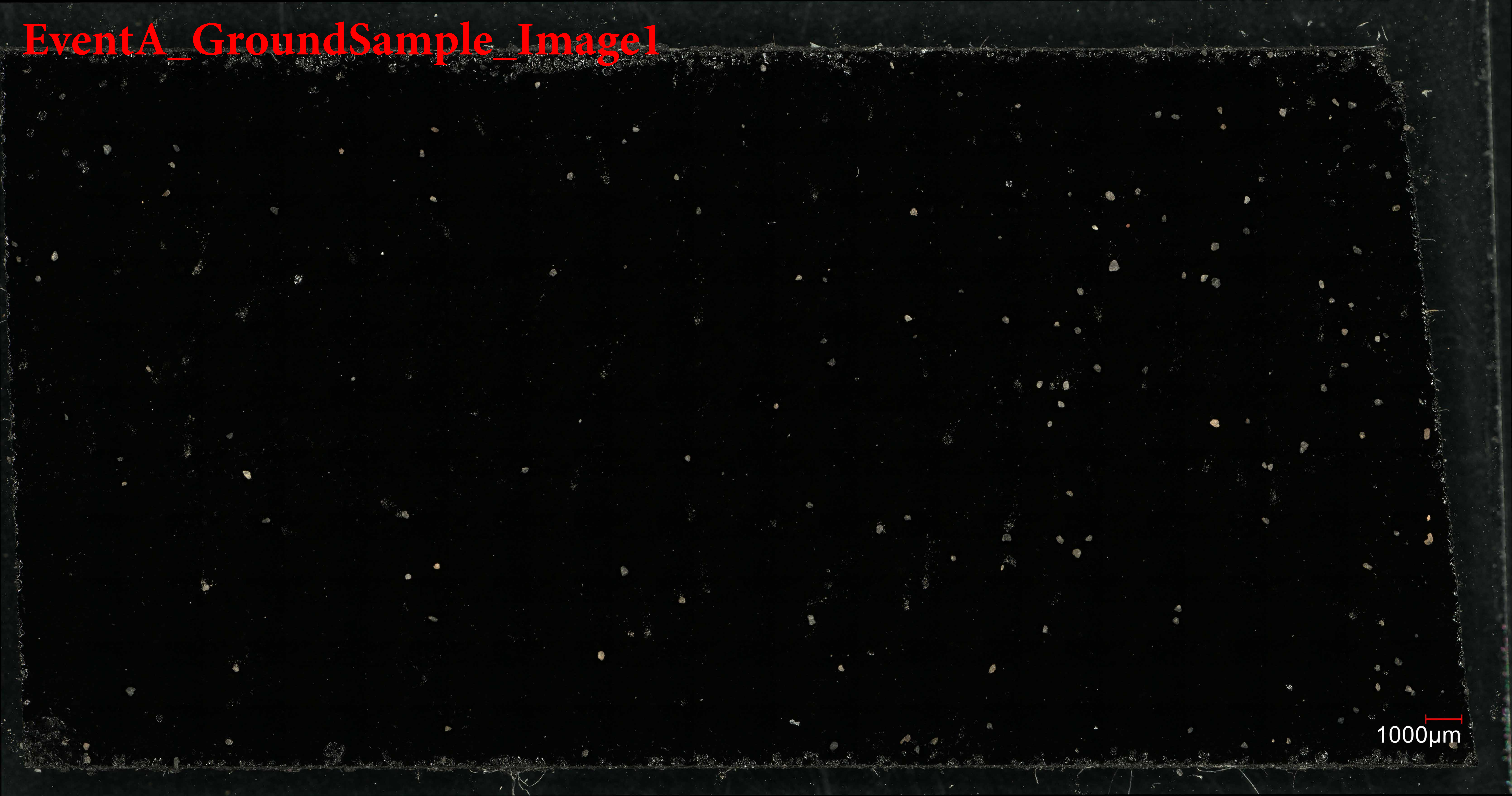
EventA_GroundSample_Image1
1000µm

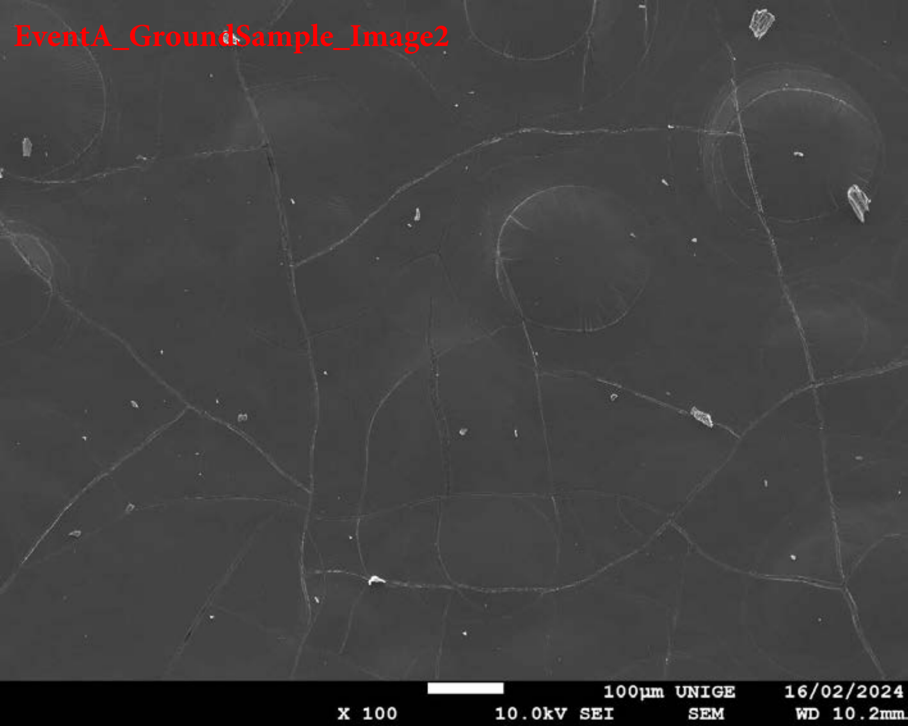
EventA_GroundSample_Image2
100µm UNIGE
16/02/2024
X 100
10.0kV SEI
SEM
WD 10.2mm

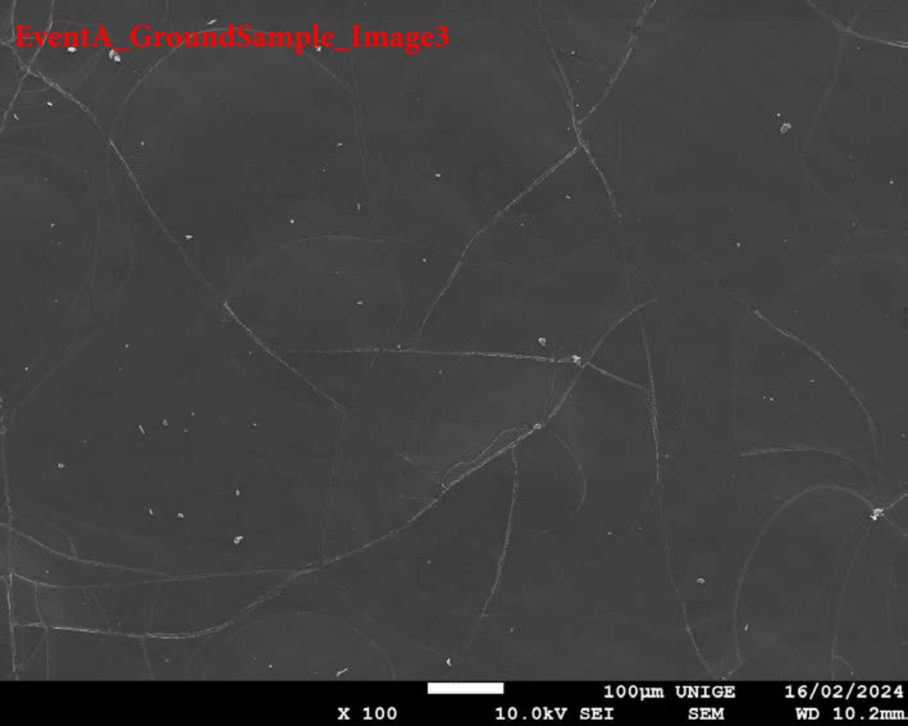
EventA_GroundSample_Image3
100µm UNIGE
16/02/2024
X 100
10.0kV SEI
SEM
WD 10.2mm

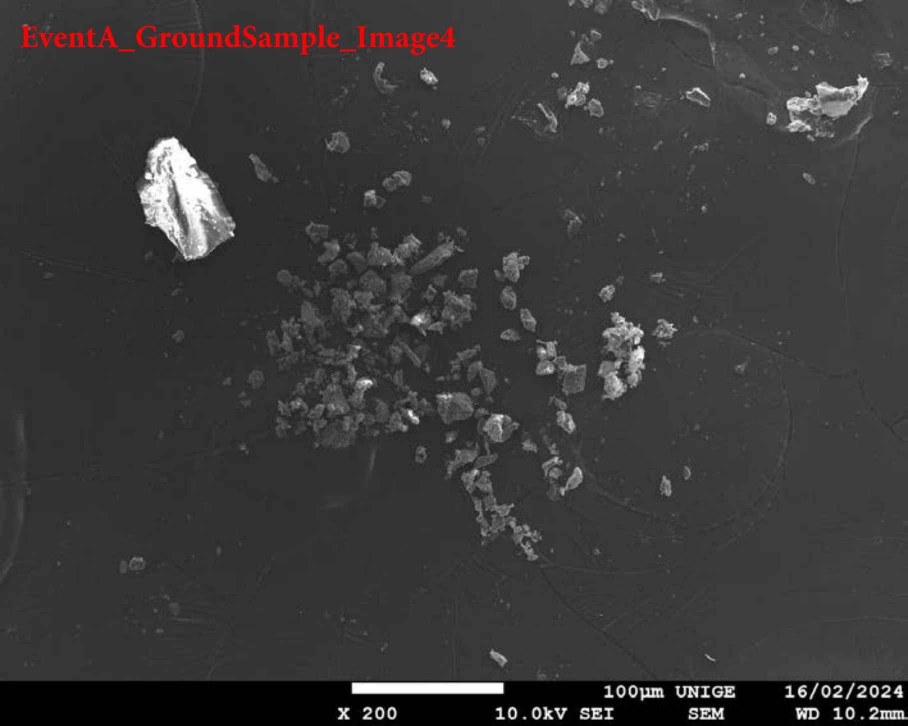
EventA_GroundSample_Image4
100µm UNIGE
16/02/2024
X 200
10.0kV SEI
SEM
WD 10.2mm

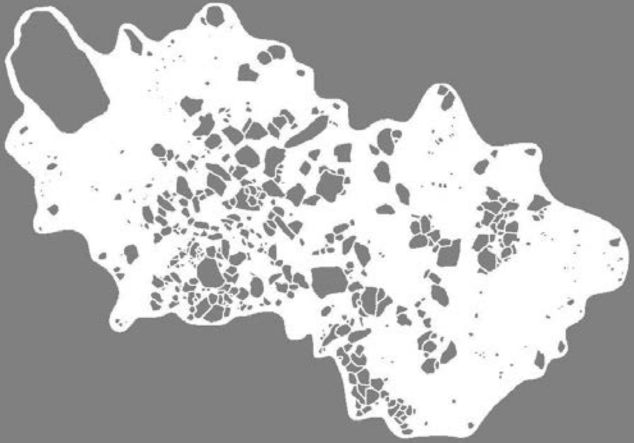

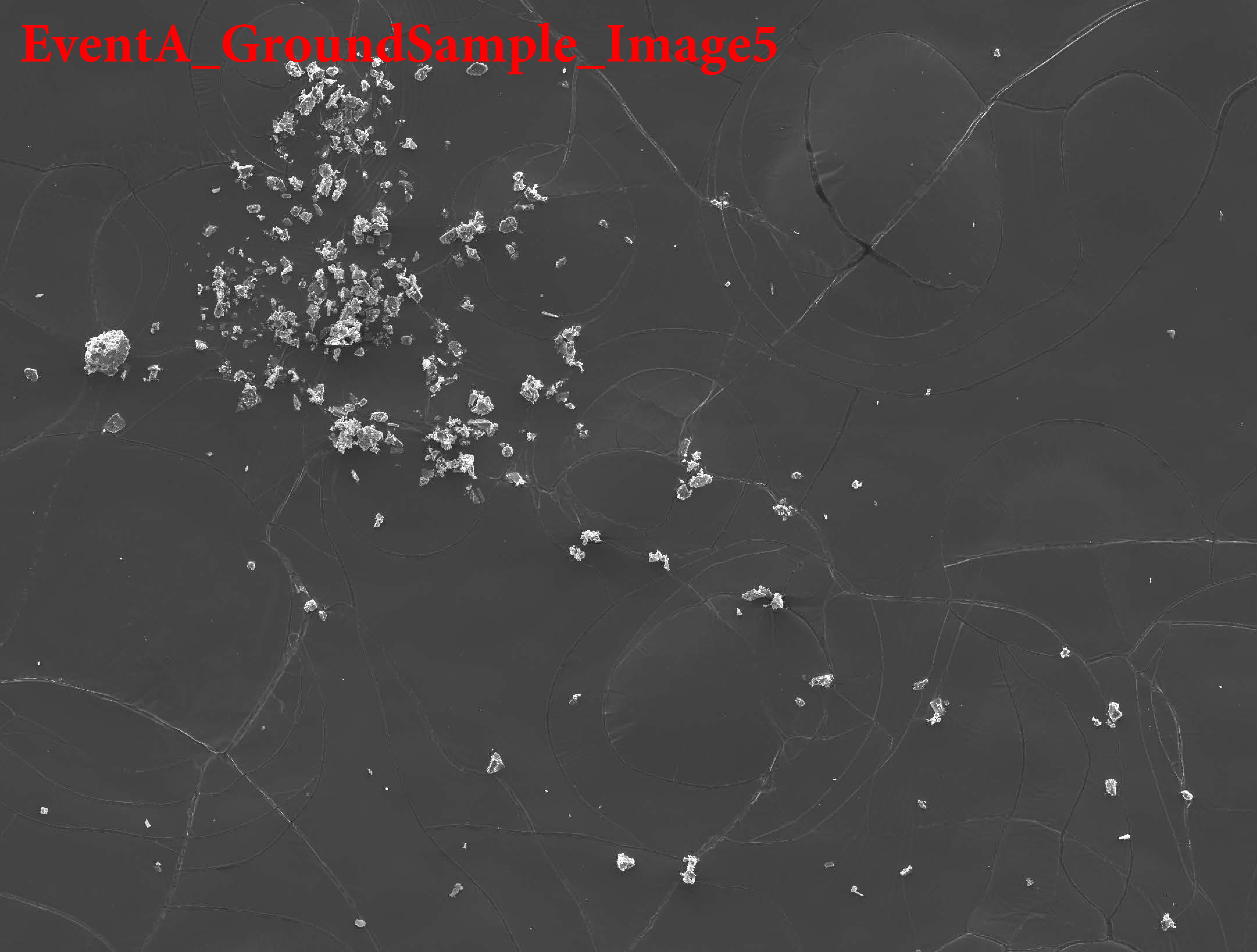
EventA_GroundSample_Image5

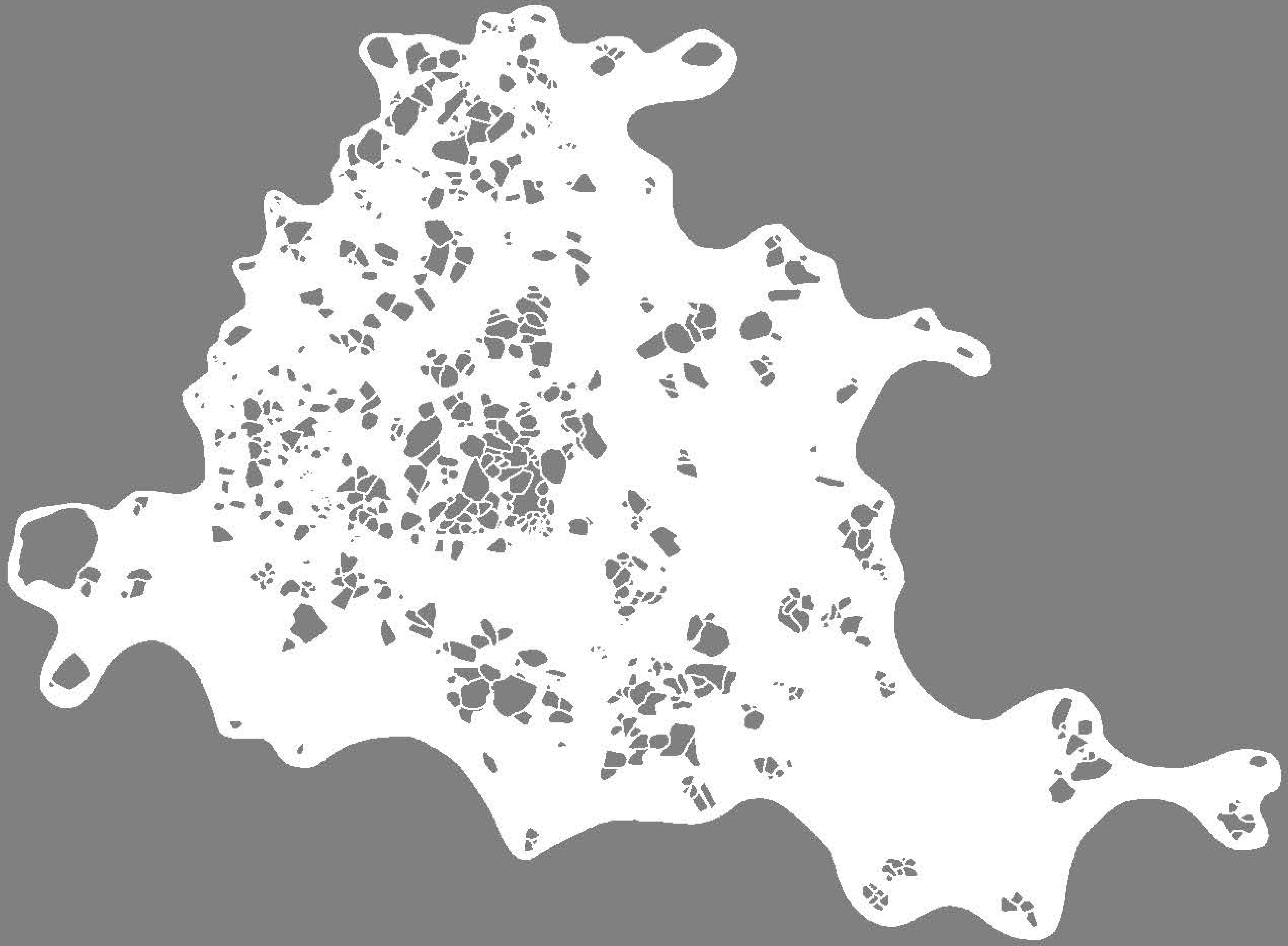

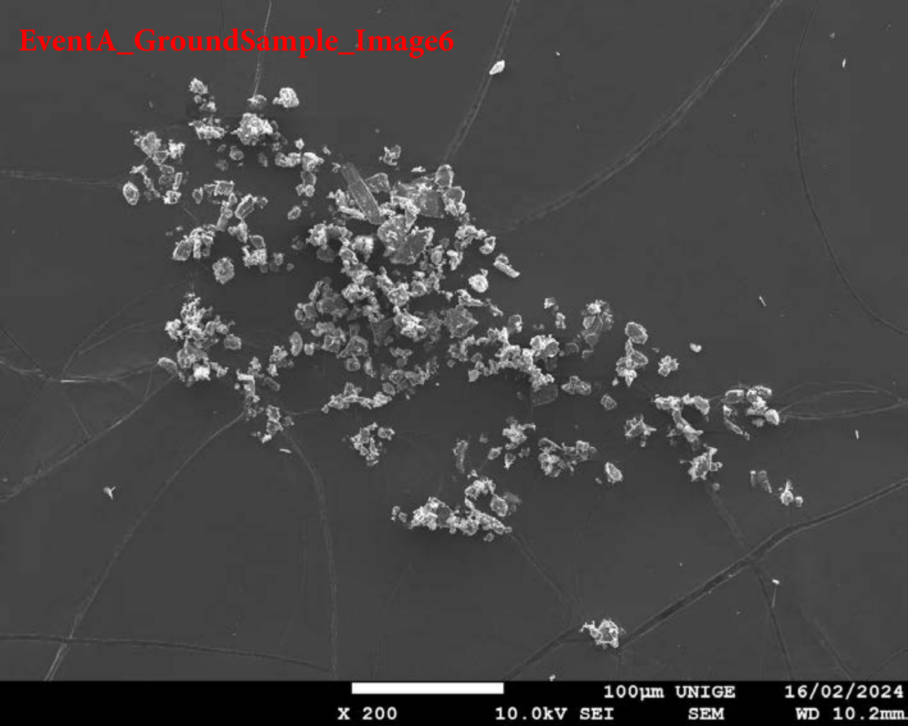
EventA_GroundSample_Image6
100µm UNIGE
16/02/2024
X 200
10.0kV SEI
SEM
WD 10.2mm

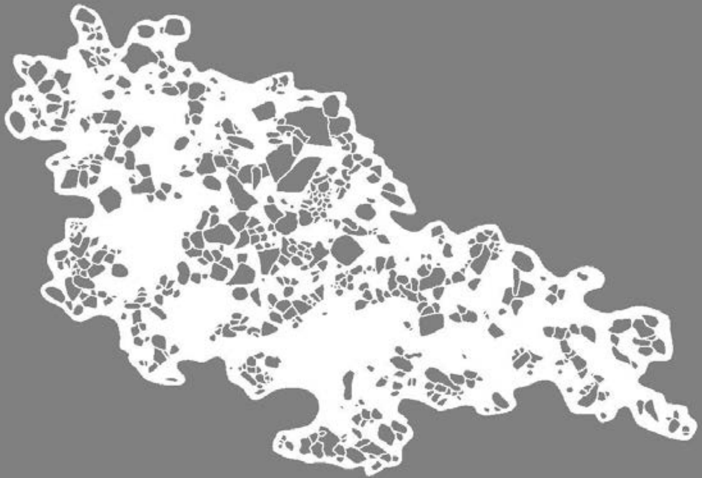

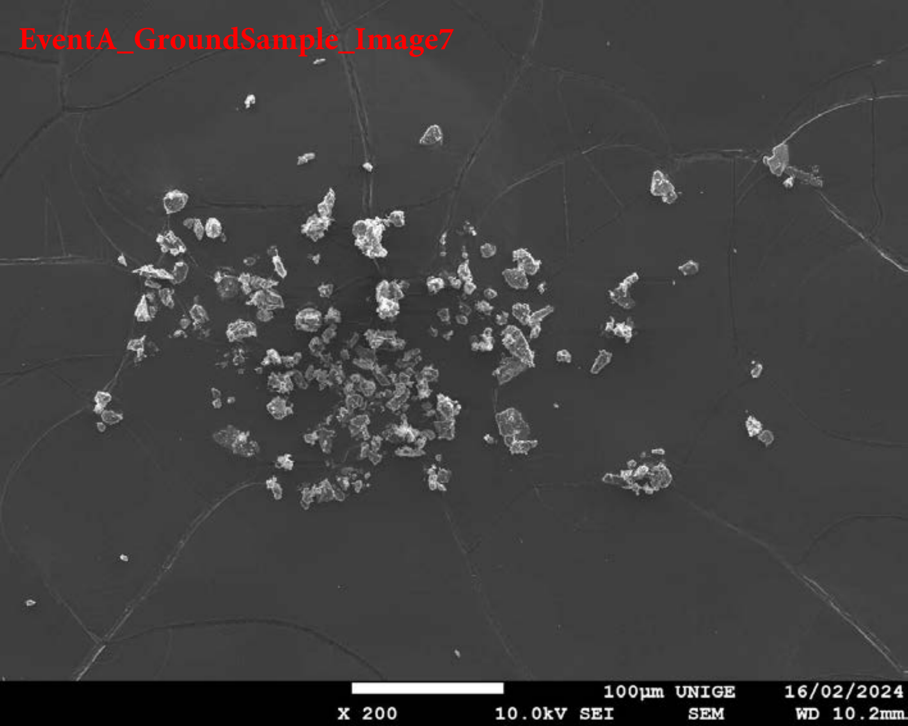
EventA_GroundSample_Image7
100µm UNIGE
16/02/2024
X 200
10.0kV SEI
SEM
WD 10.2mm

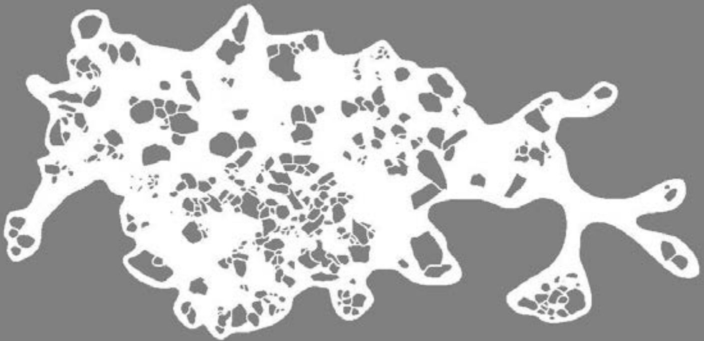

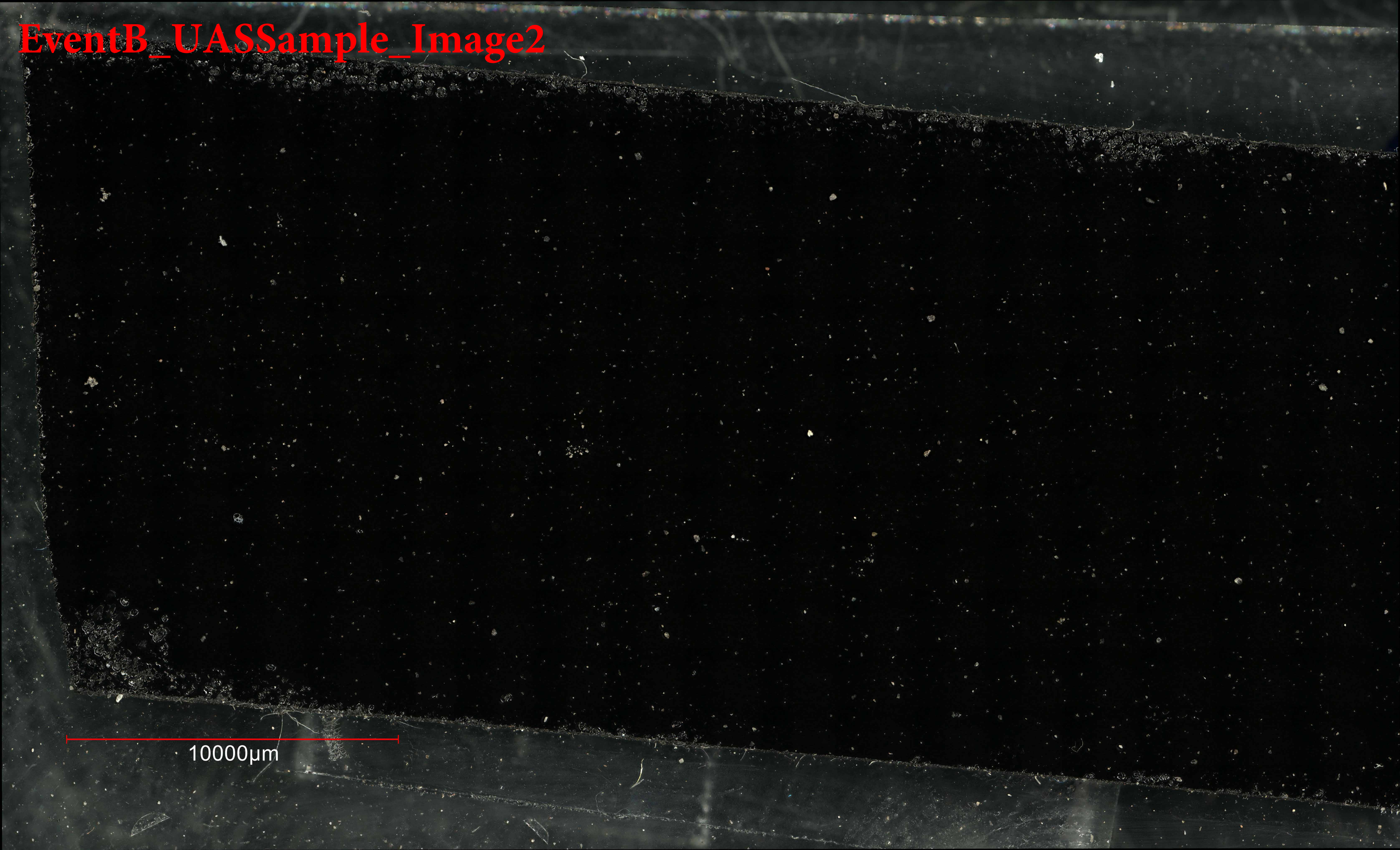
EventB_UASSample_Image2
10000μm

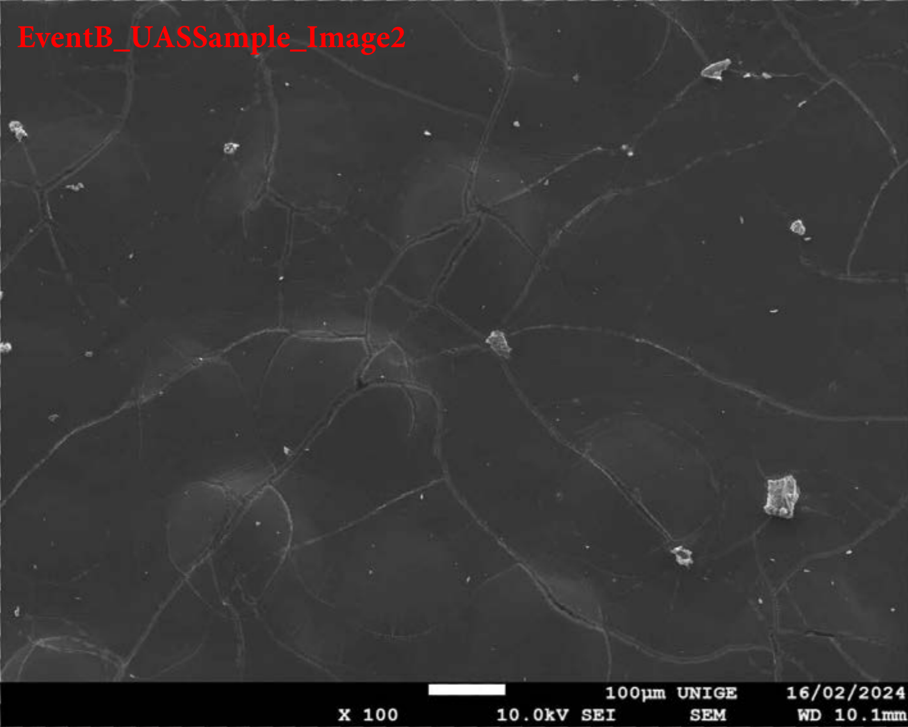
EventB_UASSample_Image2
100µm UNIGE
16/02/2024
X 100
10.0kV SEI
SEM
WD 10.1mm

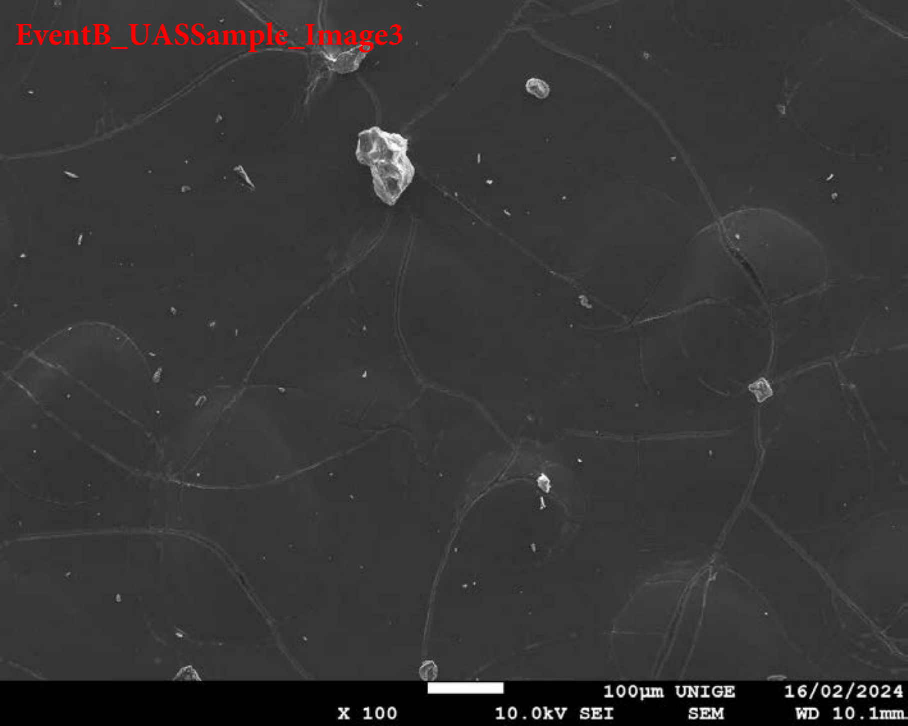
EventB_UASSample_Image3
100µm UNIGE
16/02/2024
X 100
10.0kV SEI
SEM
WD 10.1mm

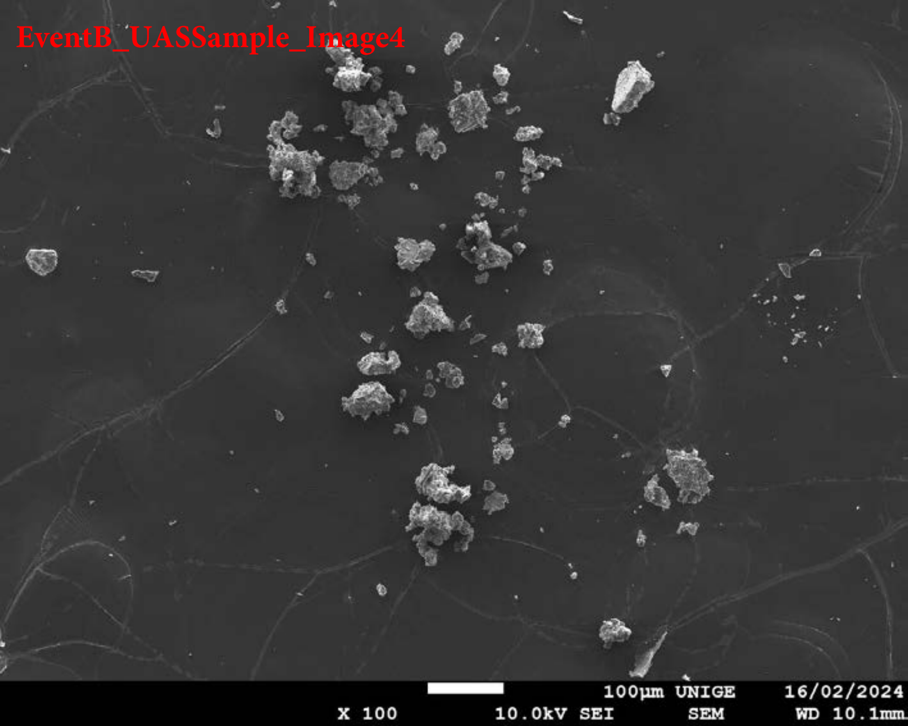
EventB_UASSample_Image4
100µm UNIGE
16/02/2024
X 100
10.0kV SEI
SEM
WD 10.1mm

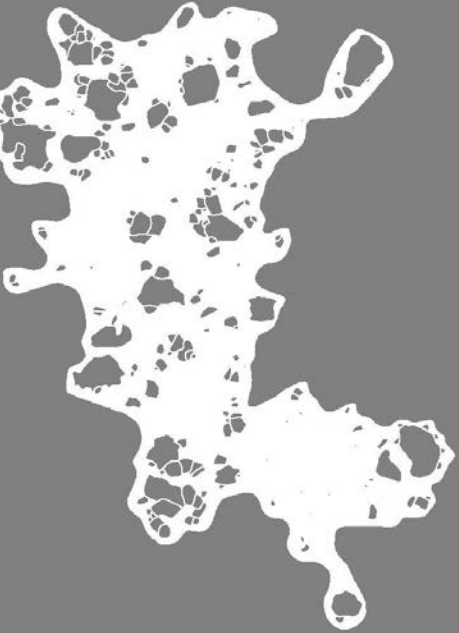

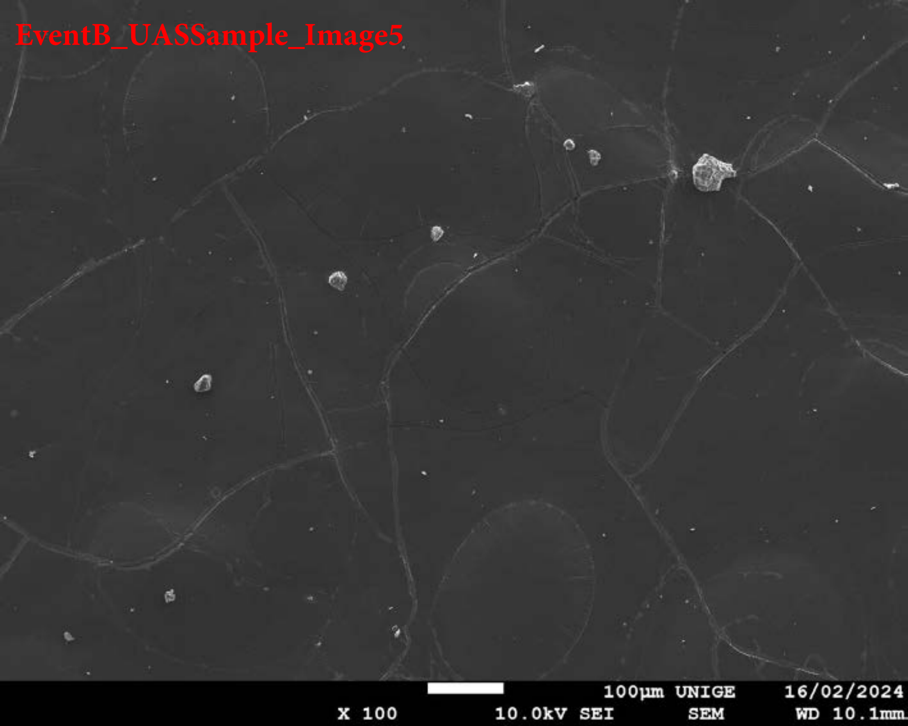
EventB_UASSample_Image5
100µm UNIGE
16/02/2024
X 100
10.0kV SEI
SEM
WD 10.1mm

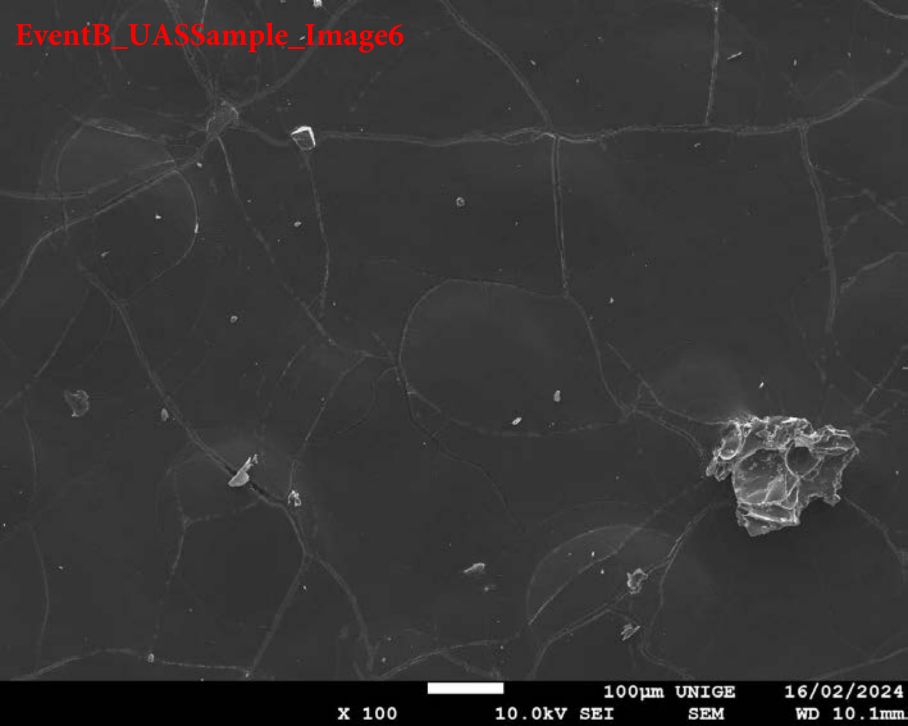
EventB_UASSample_Image6
100µm UNIGE
16/02/2024
X 100
10.0kV SEI
SEM
WD 10.1mm

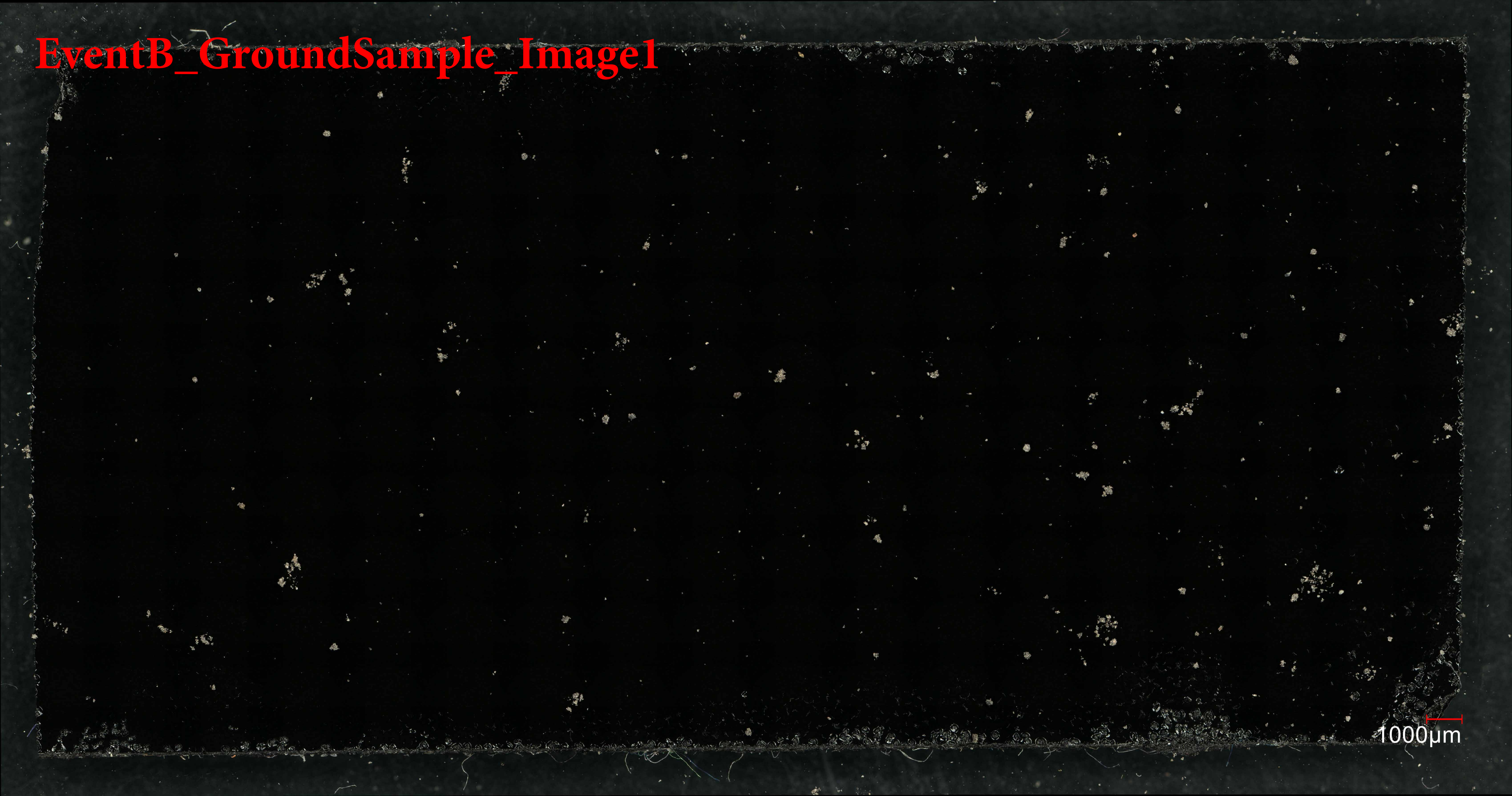
EventB_GroundSample_Image1
1000μm

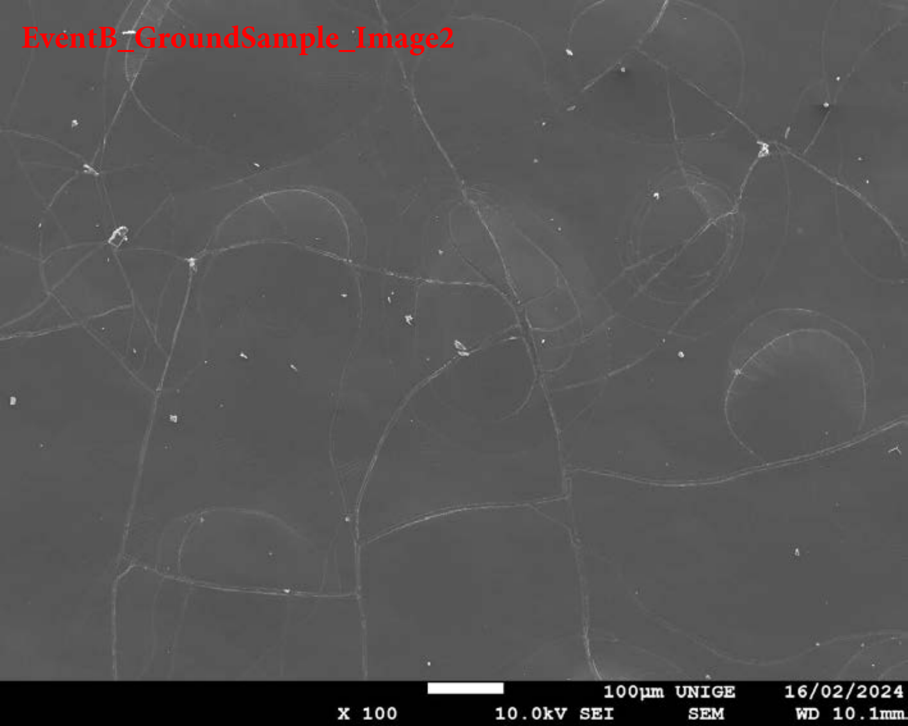
EventB_GroundSample_Image2
100µm UNIGE
16/02/2024
X 100
10.0kV SEI
SEM
WD 10.1mm

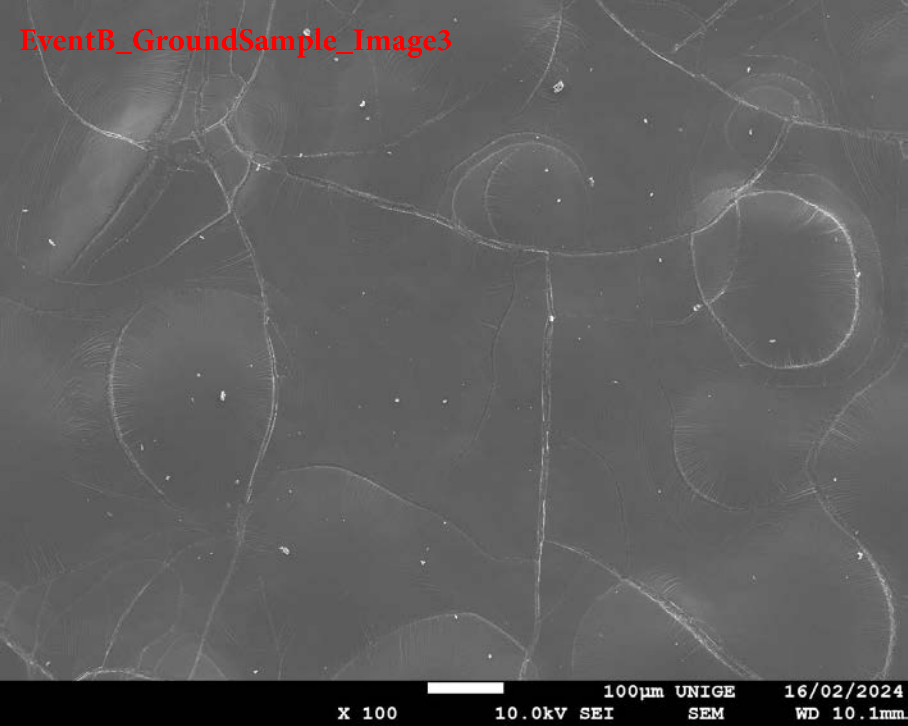
EventB_GroundSample_Image3
100µm UNIGE
16/02/2024
X 100
10.0kV SEI
SEM
WD 10.1mm

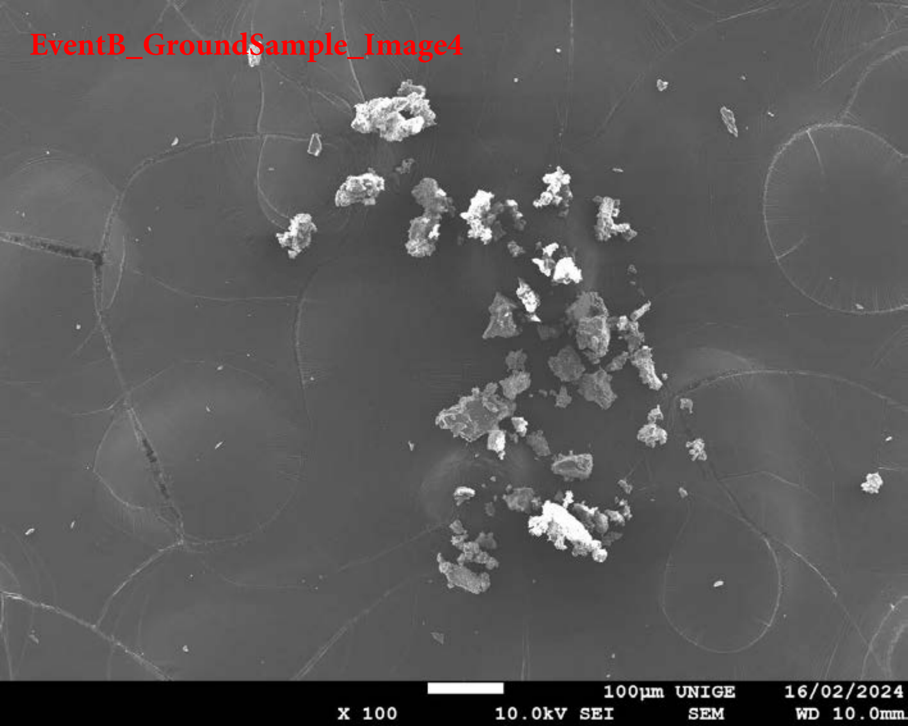
EventB_GroundSample_Image4
100µm UNIGE
16/02/2024
X 100
10.0kV SEI
SEM
WD 10.0mm

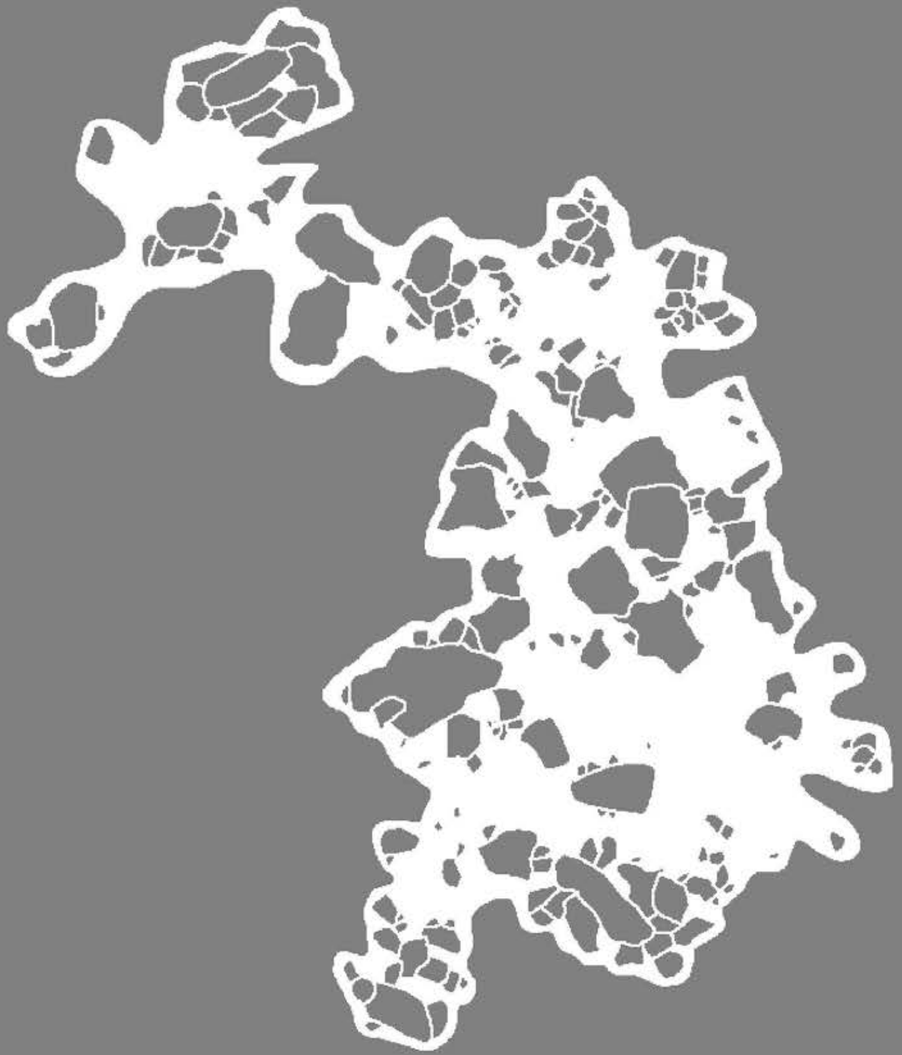

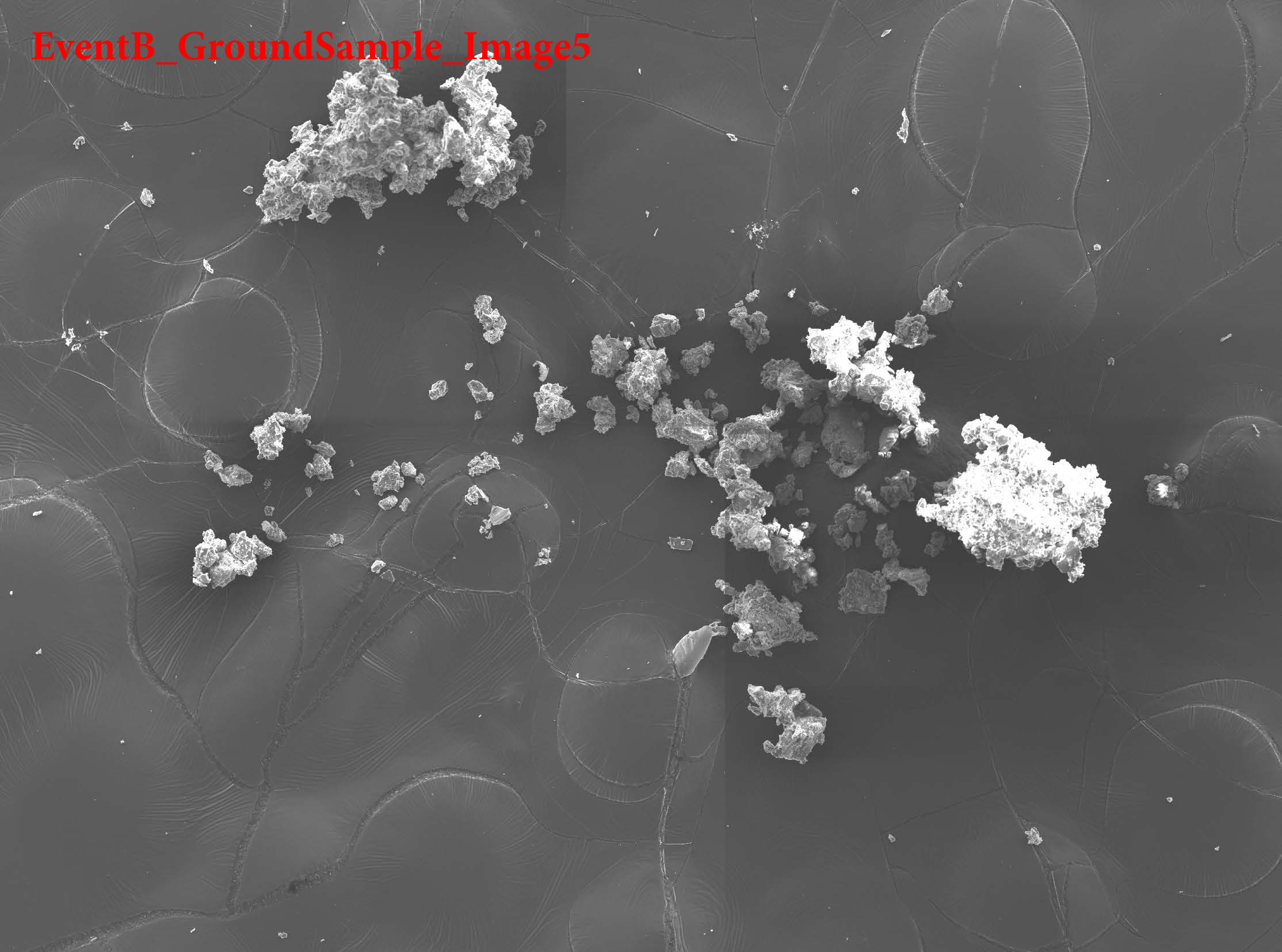
EventB_GroundSample_Image5

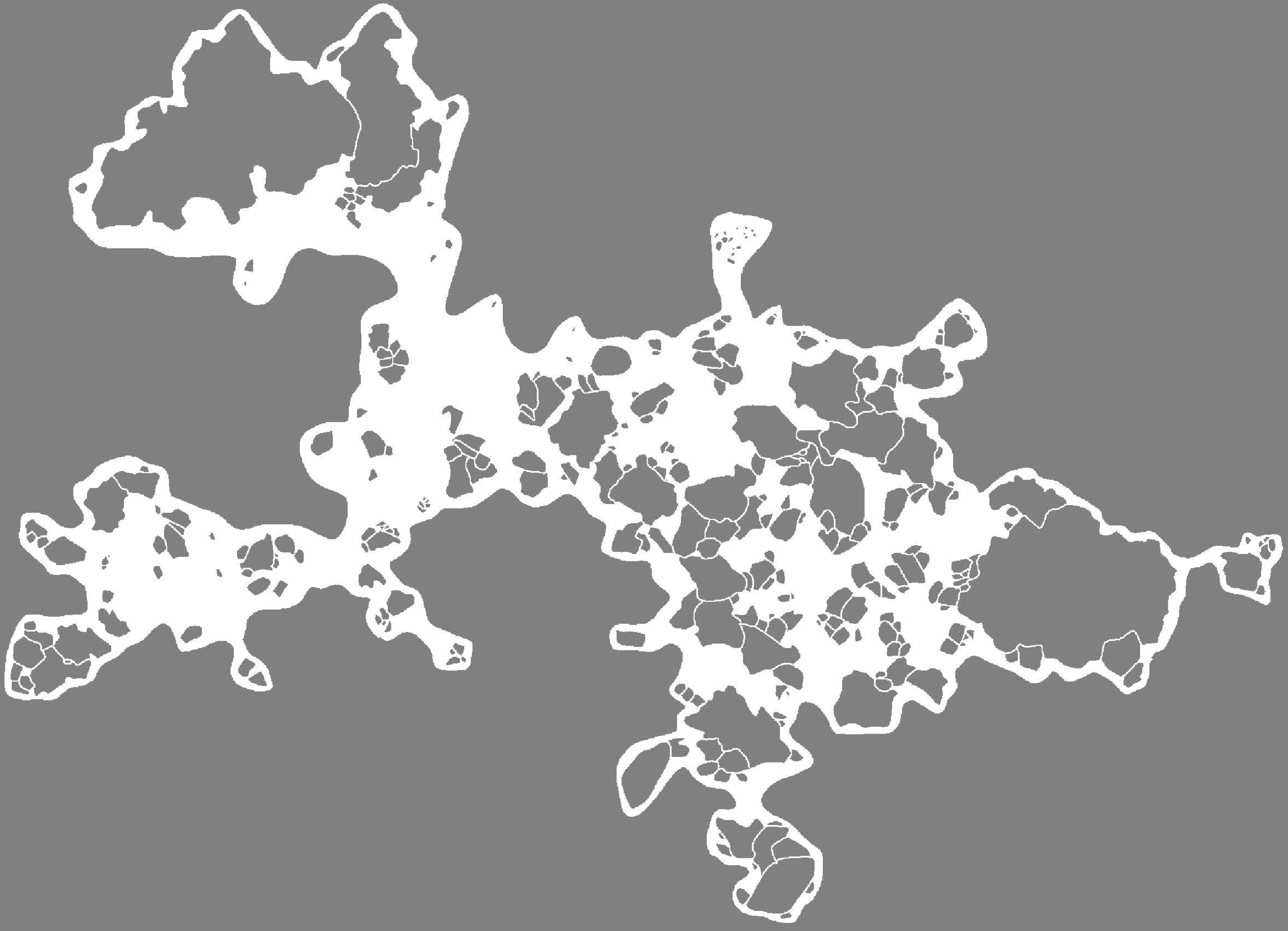

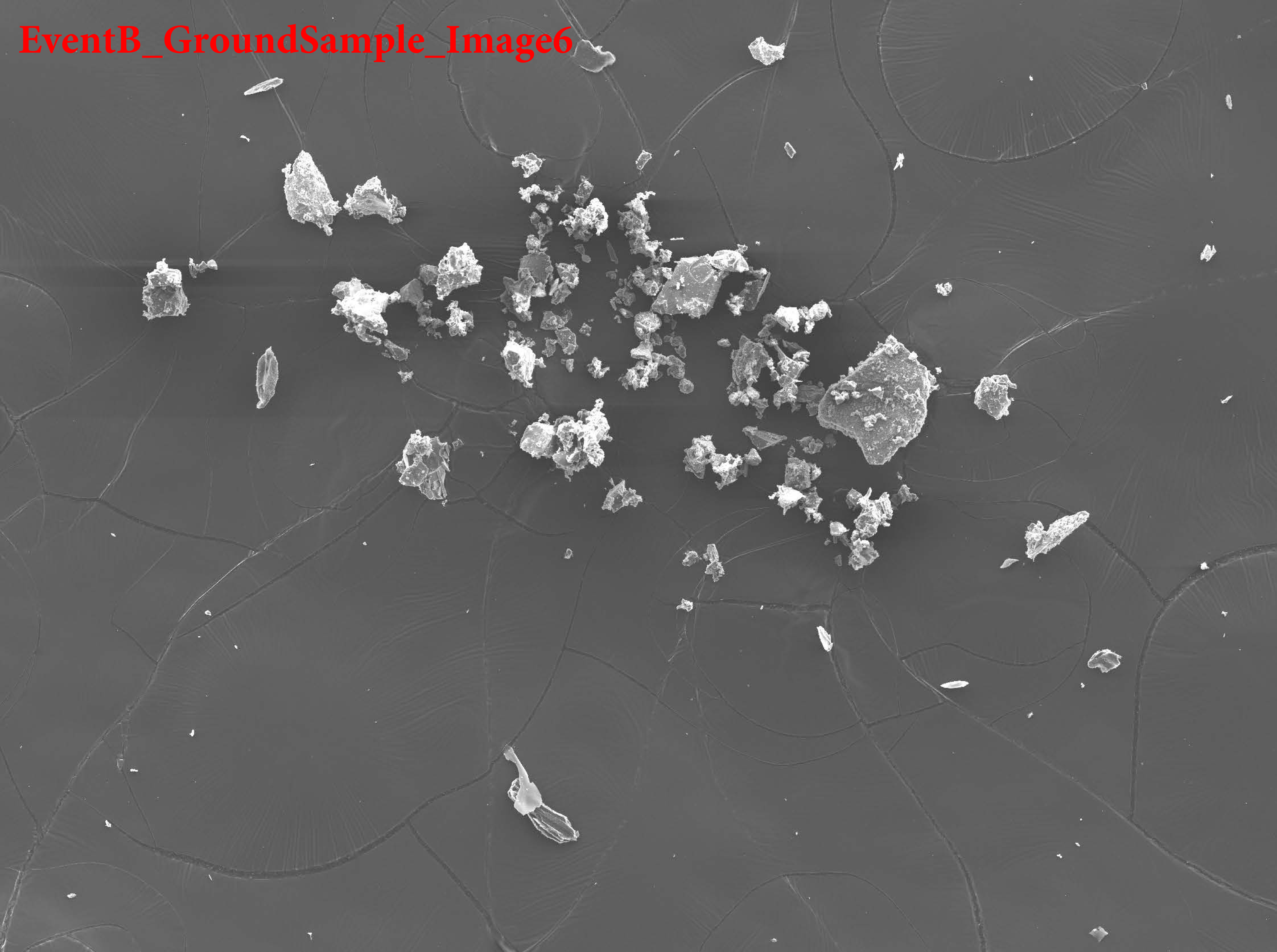
EventB_GroundSample_Image6

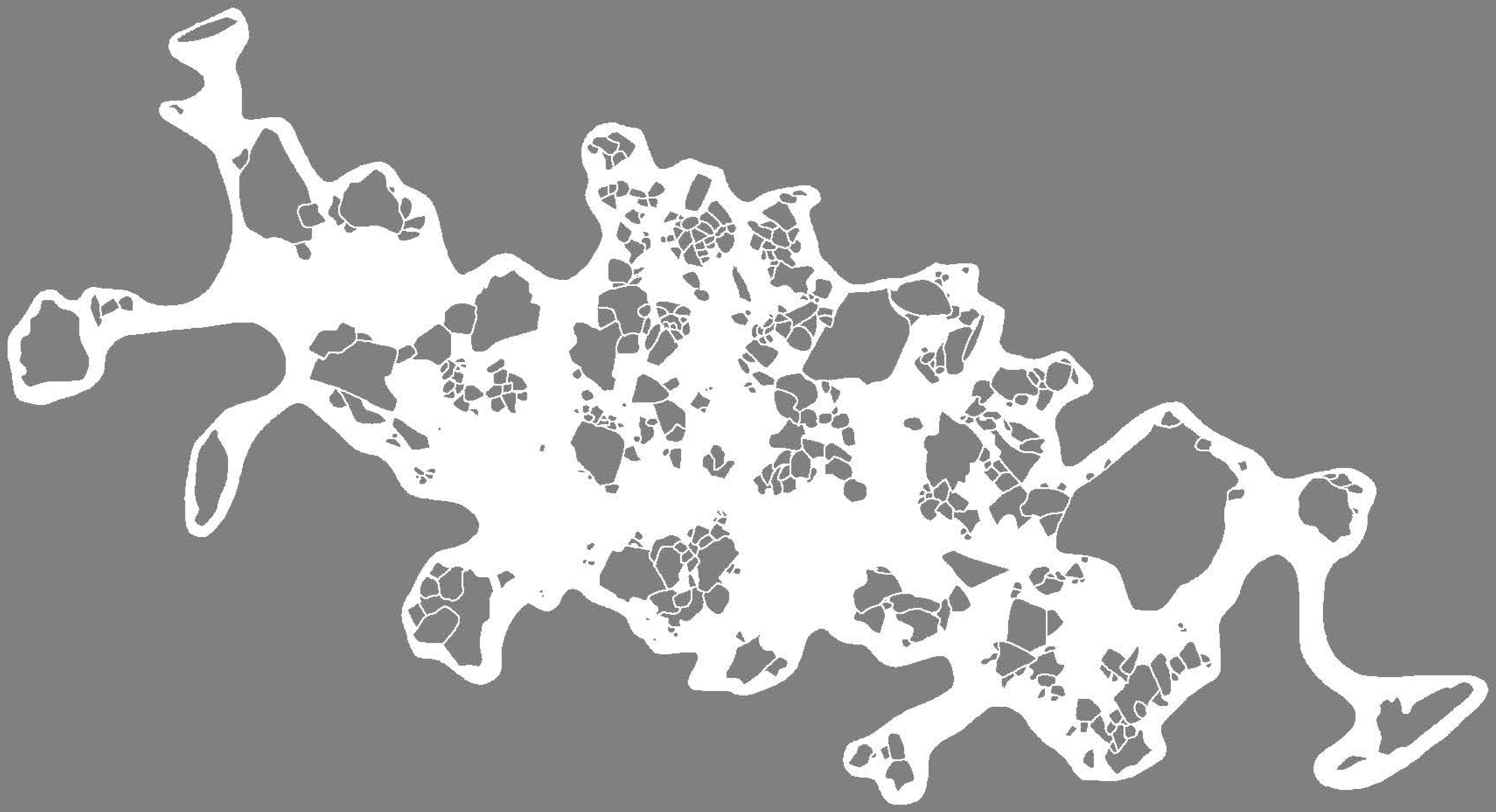

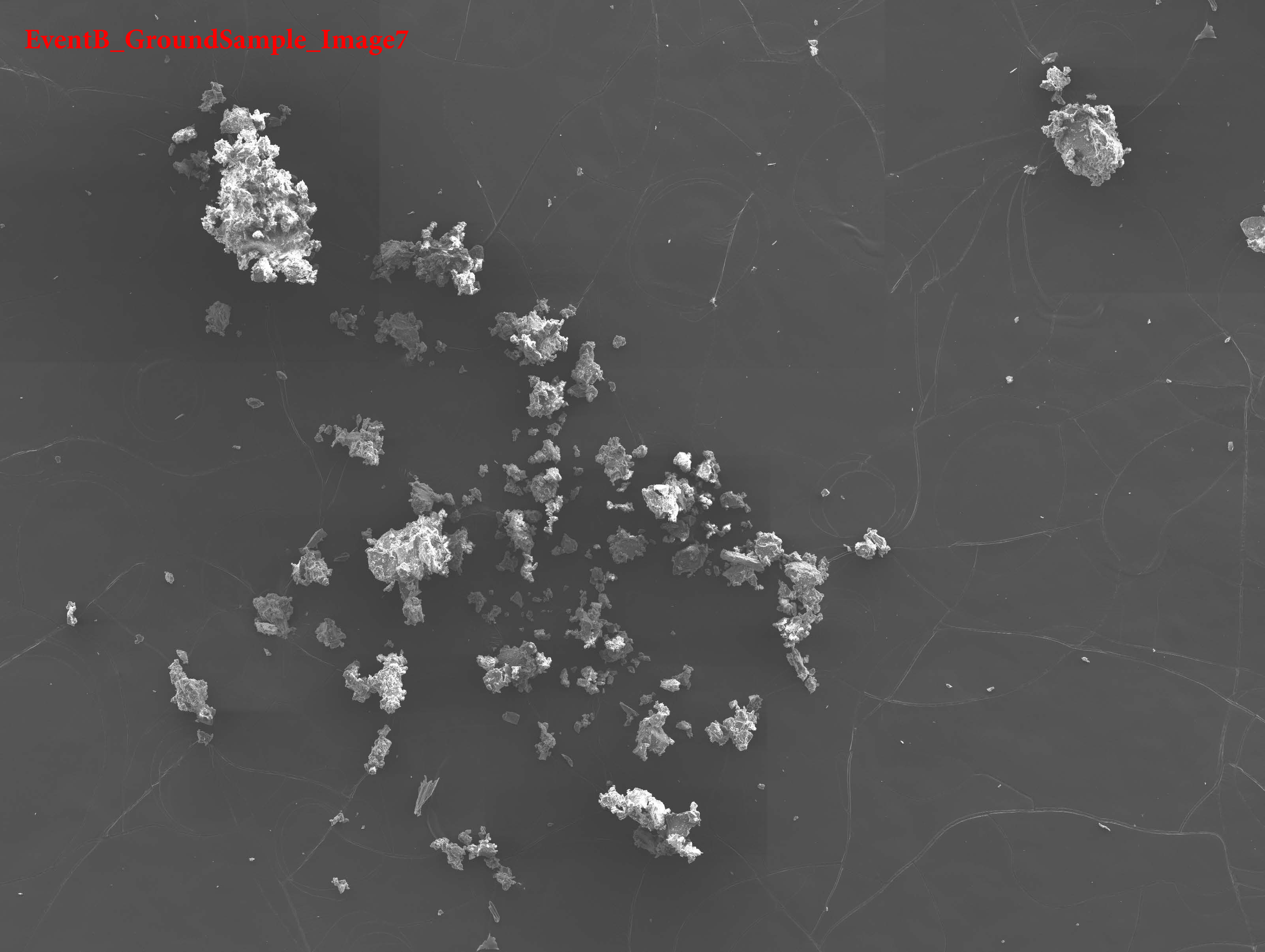
EventB_GroundSample_Image7

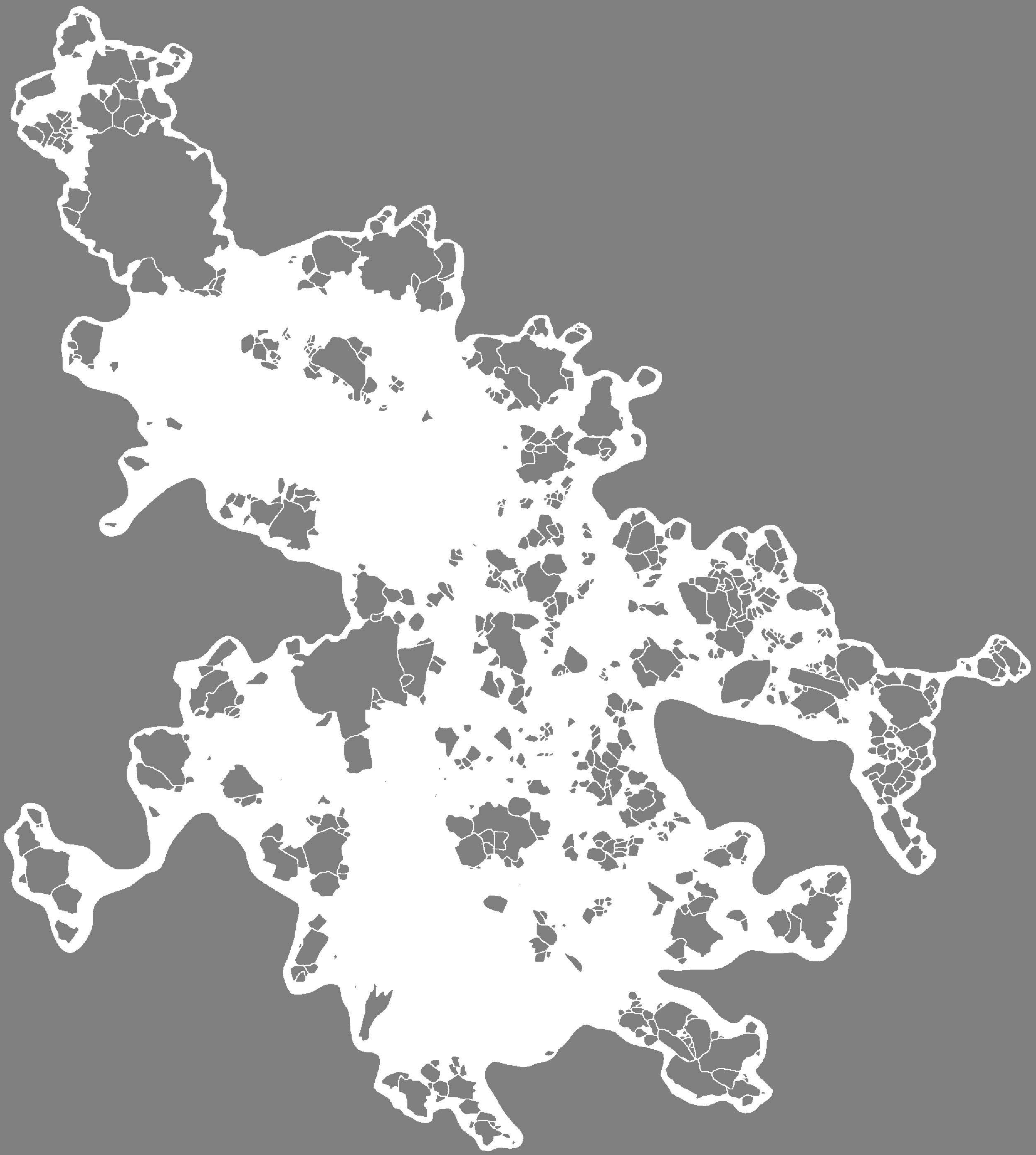

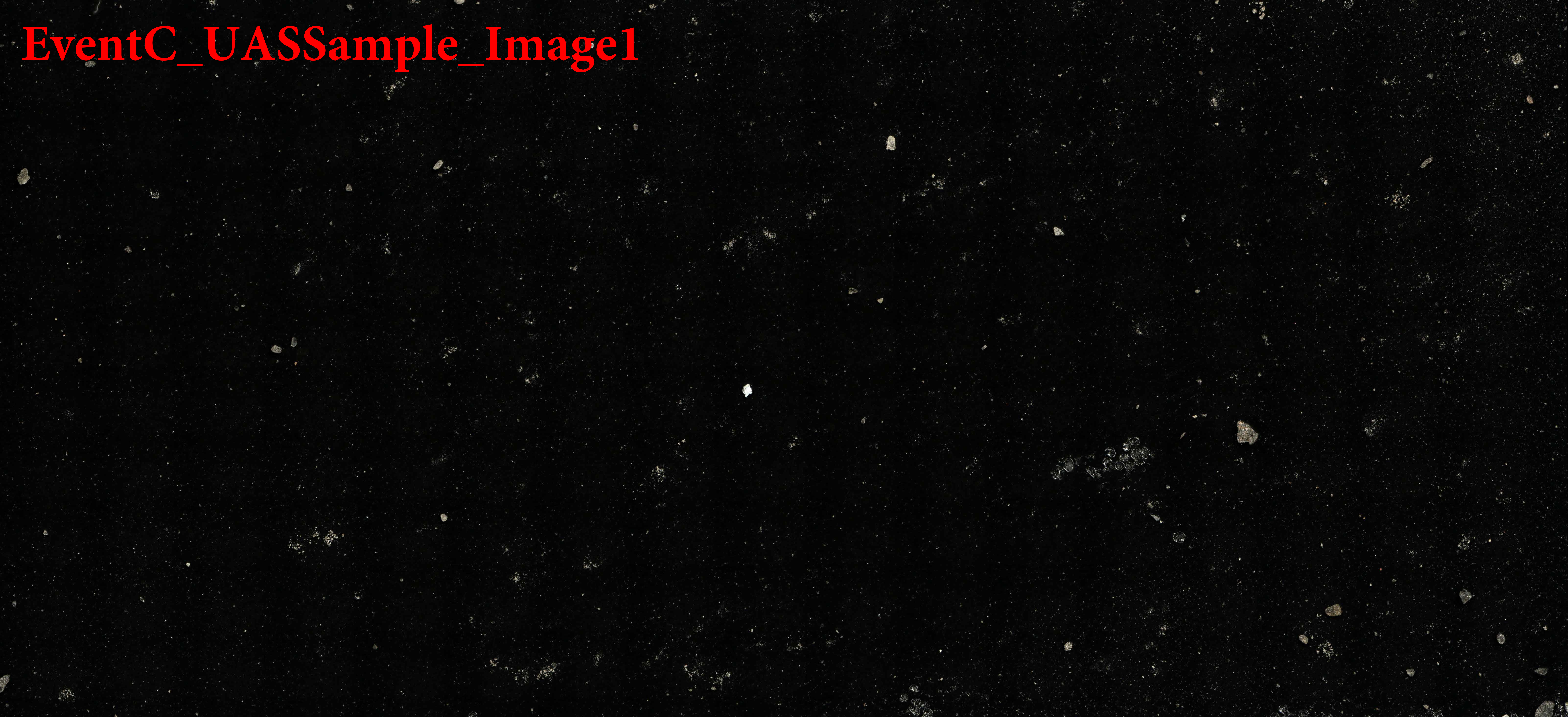
EventC_UASSample_Image1

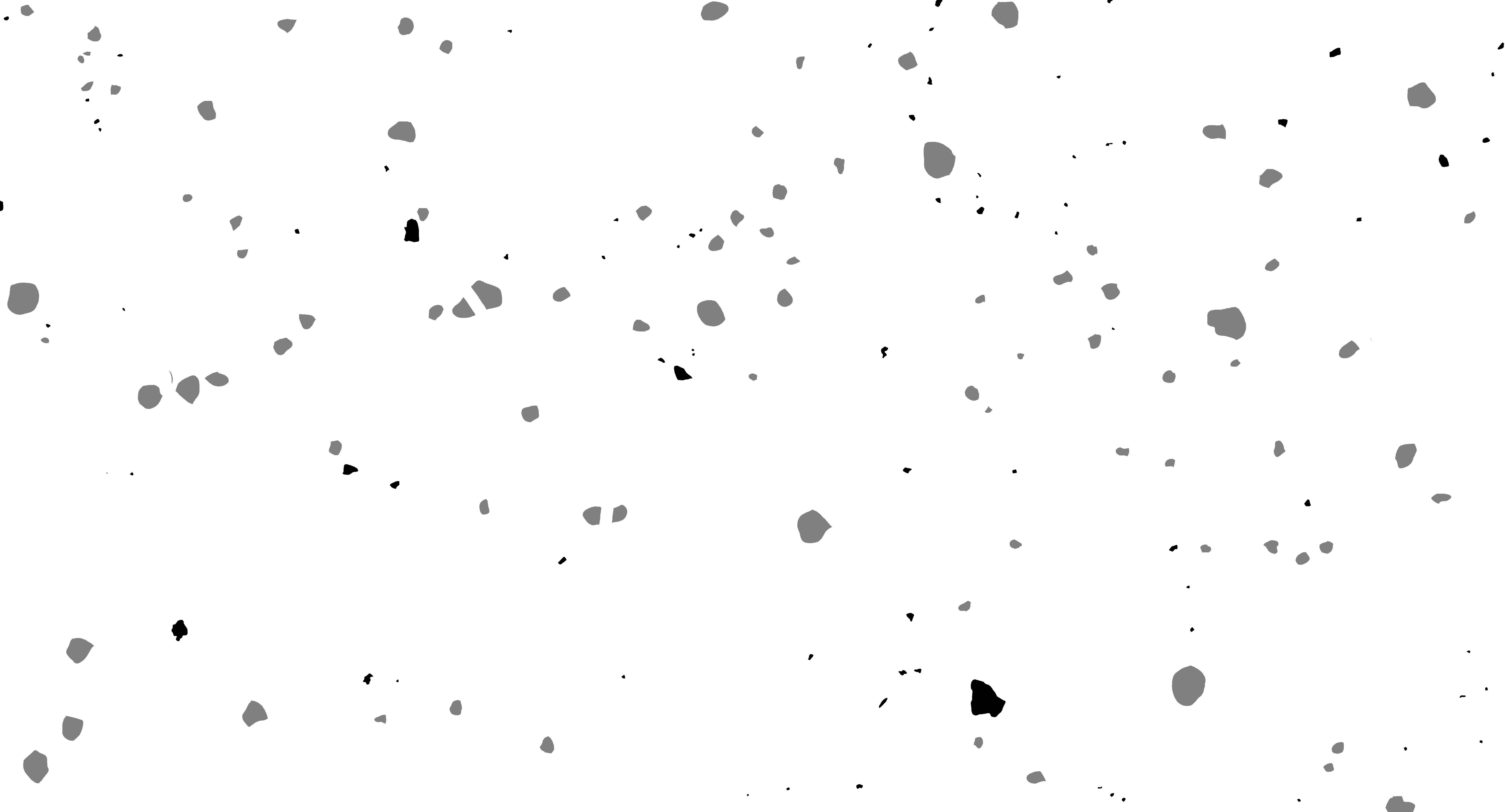

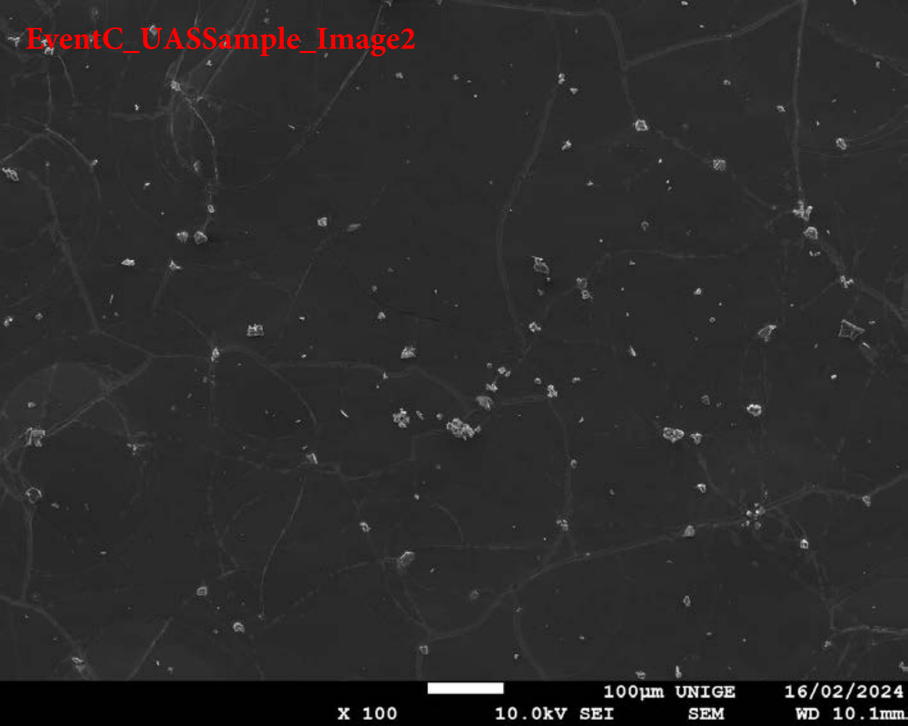

EventC_UASSample_Image2
100µm UNIGE
16/02/2024
X 100
10.0kV SEI
SEM
WD 10.1mm

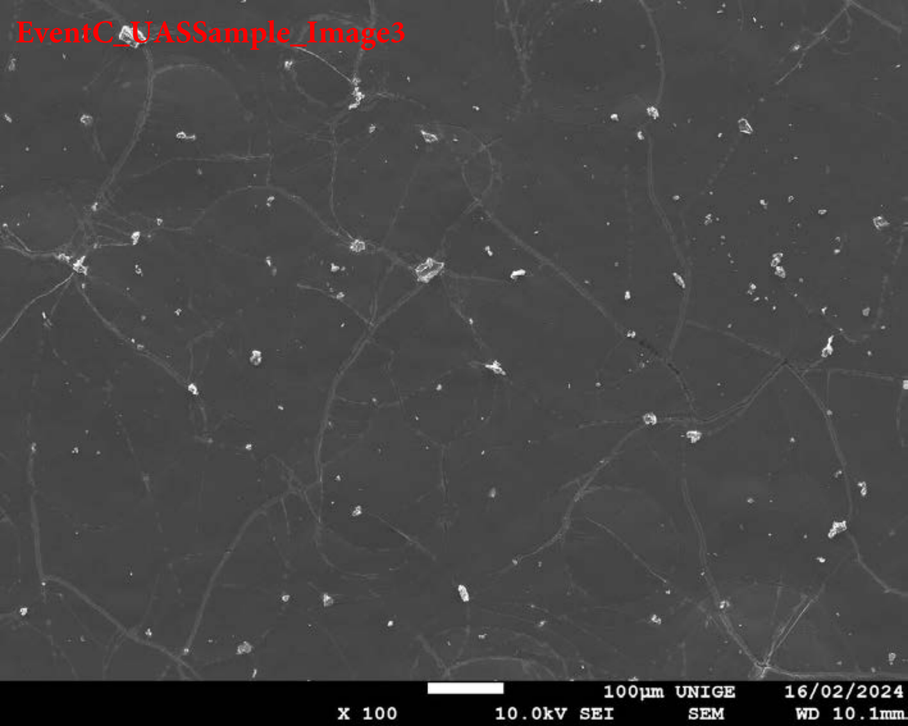
EventC_UASSample_Image3
100µm UNIGE
16/02/2024
X 100
10.0kV SEI
SEM
WD 10.1mm

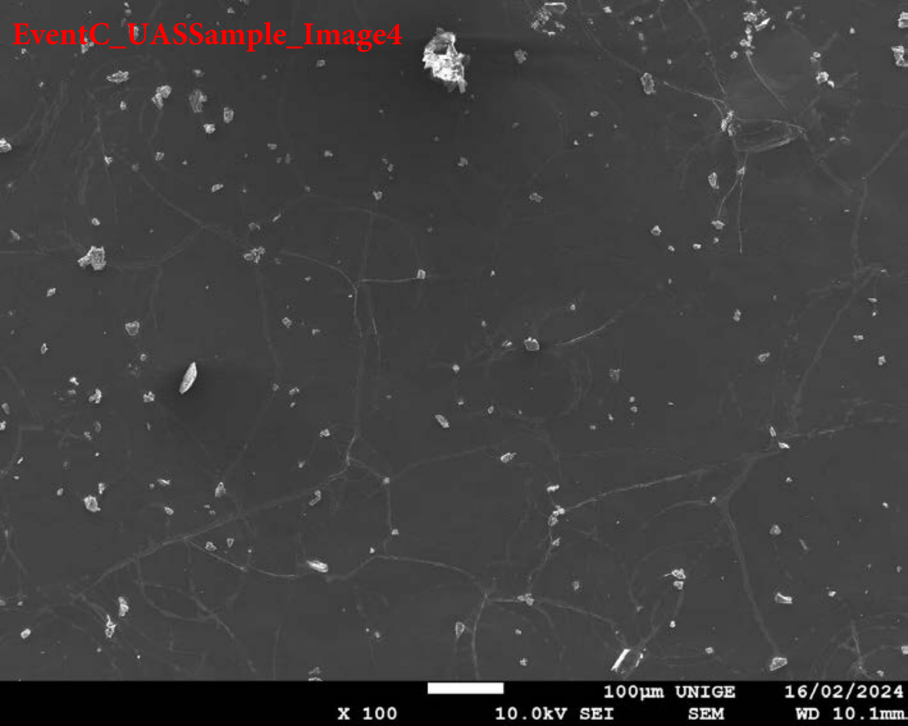
EventC_UASSample_Image4
100µm UNIGE
16/02/2024
X 100
10.0kV SEI
SEM
WD 10.1mm

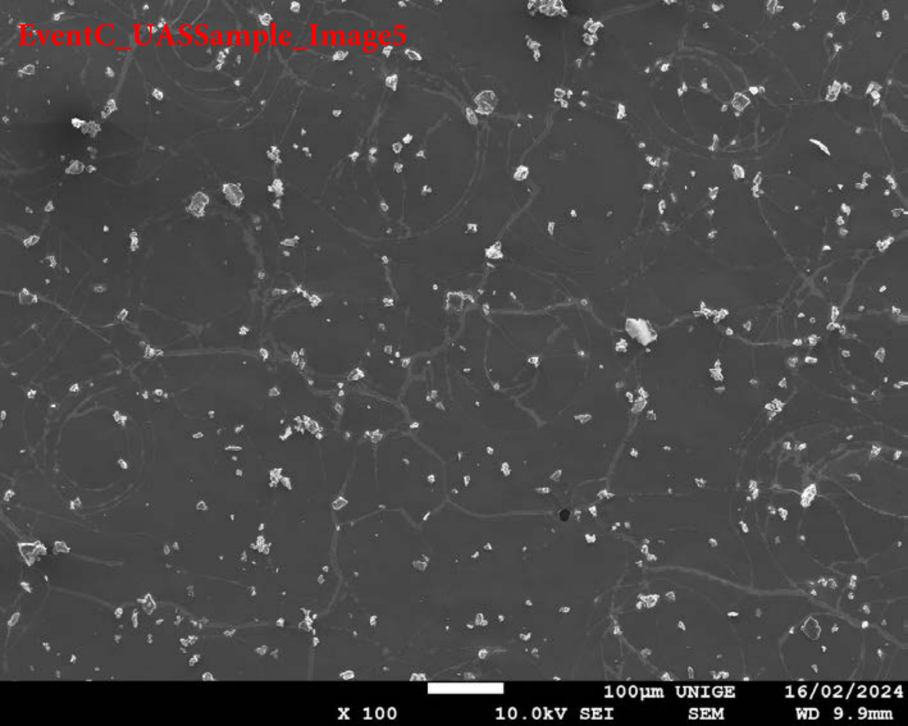
EventC_UASSample_Image5
100μm UNIGE 16/02/2024
X 100 10.0kV SEI SEM WD 9.9mm

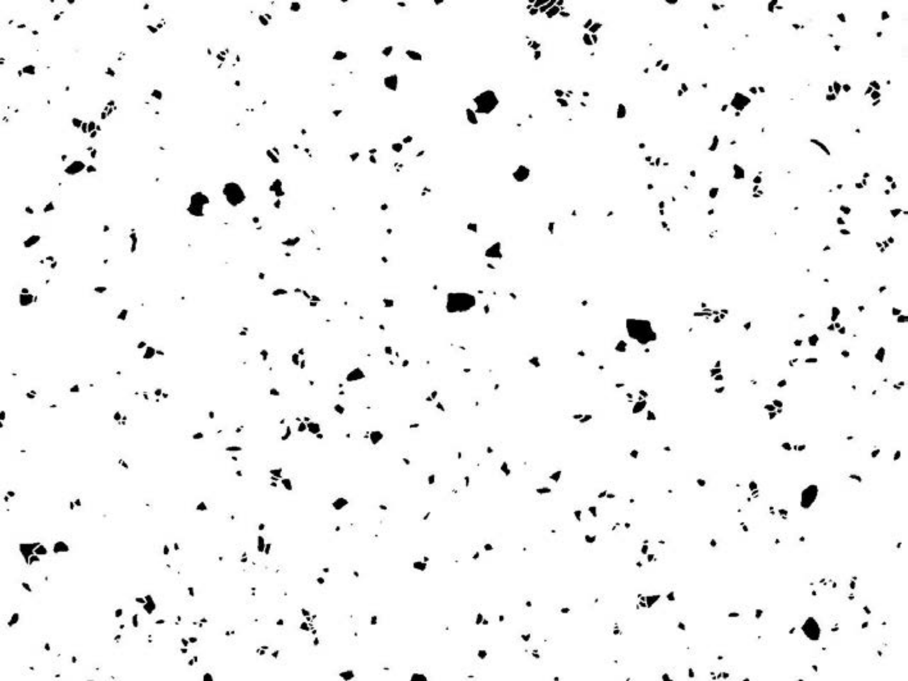

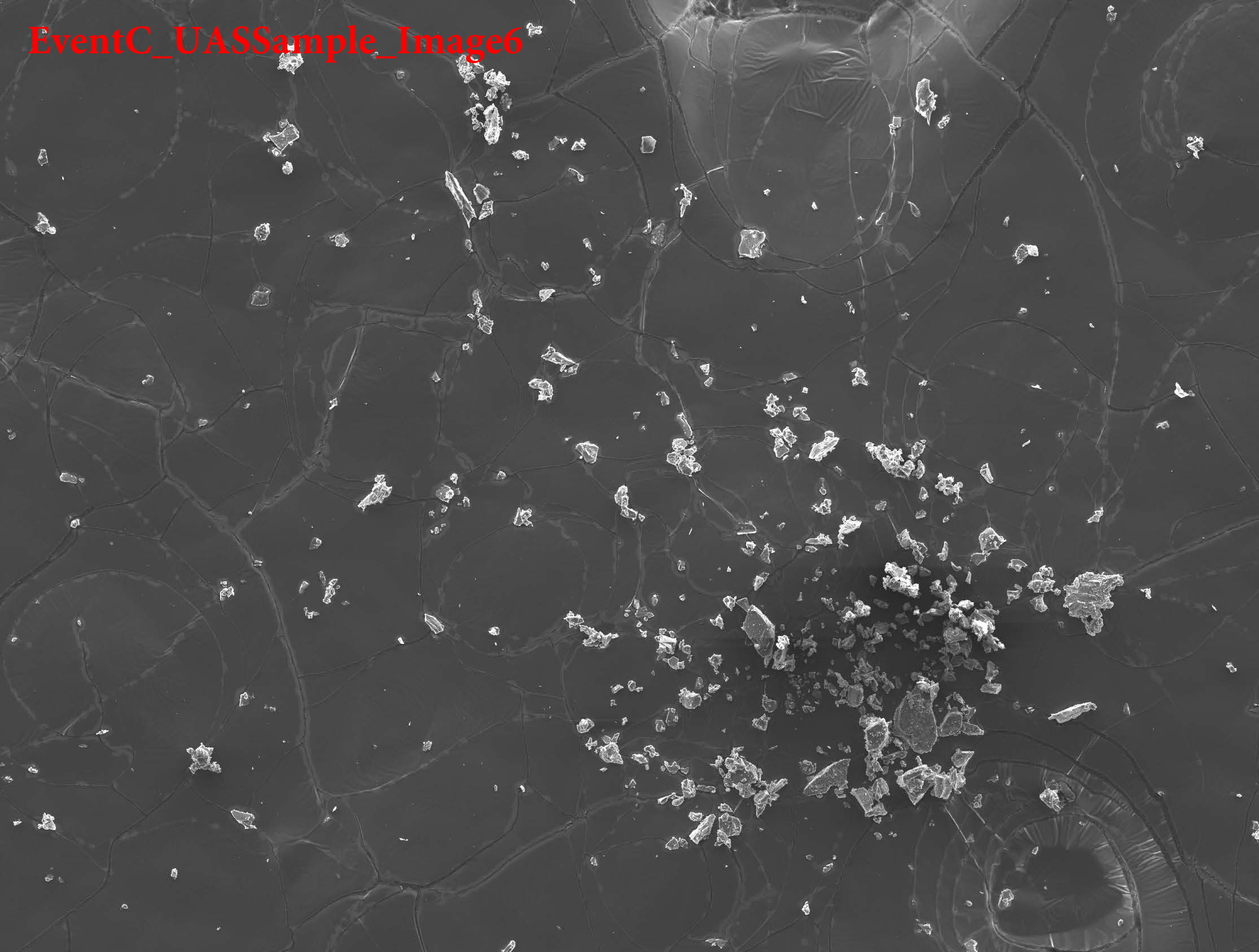
EventC_UASSample_Image6

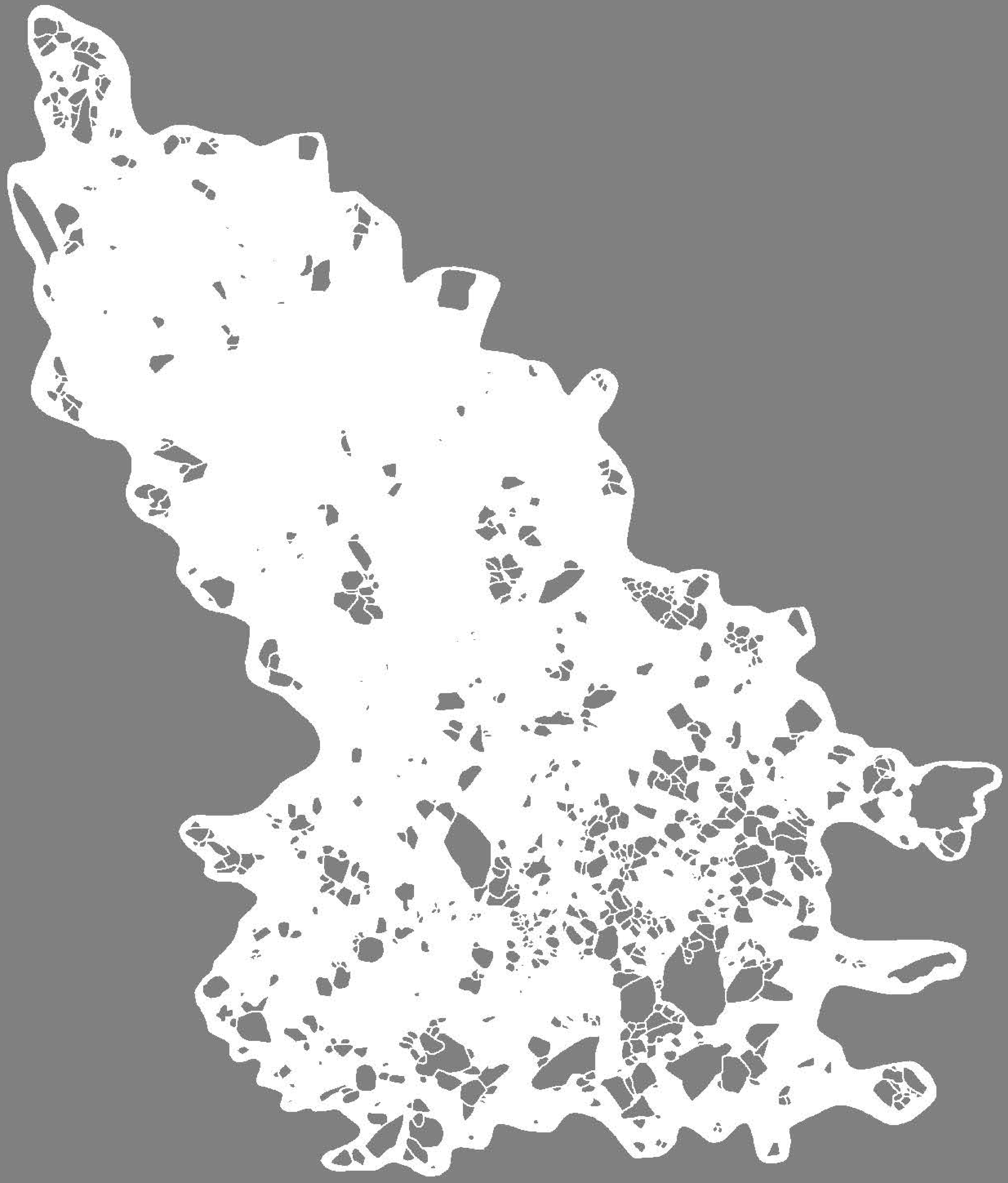

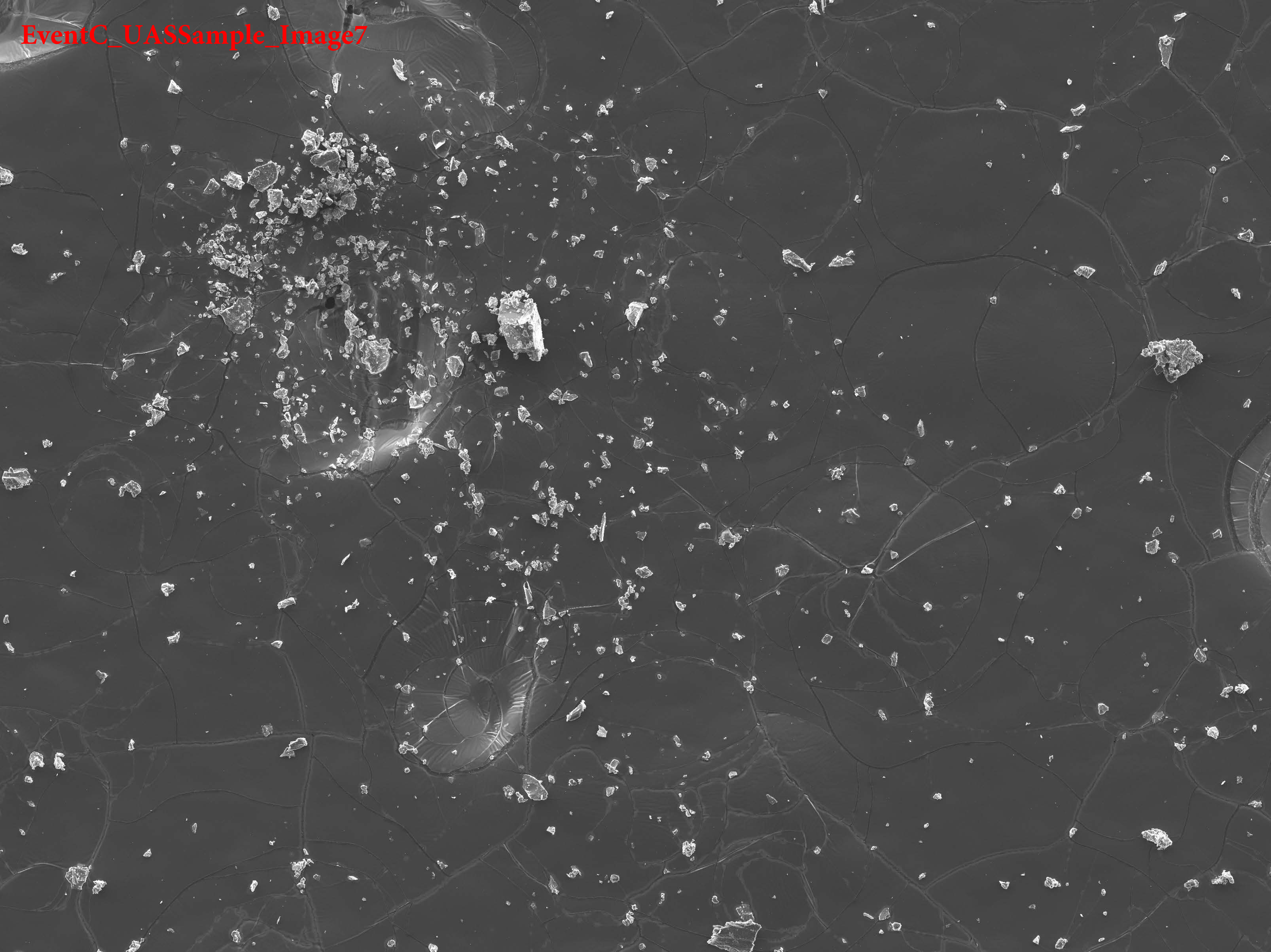
EventC_UASSample_Image7

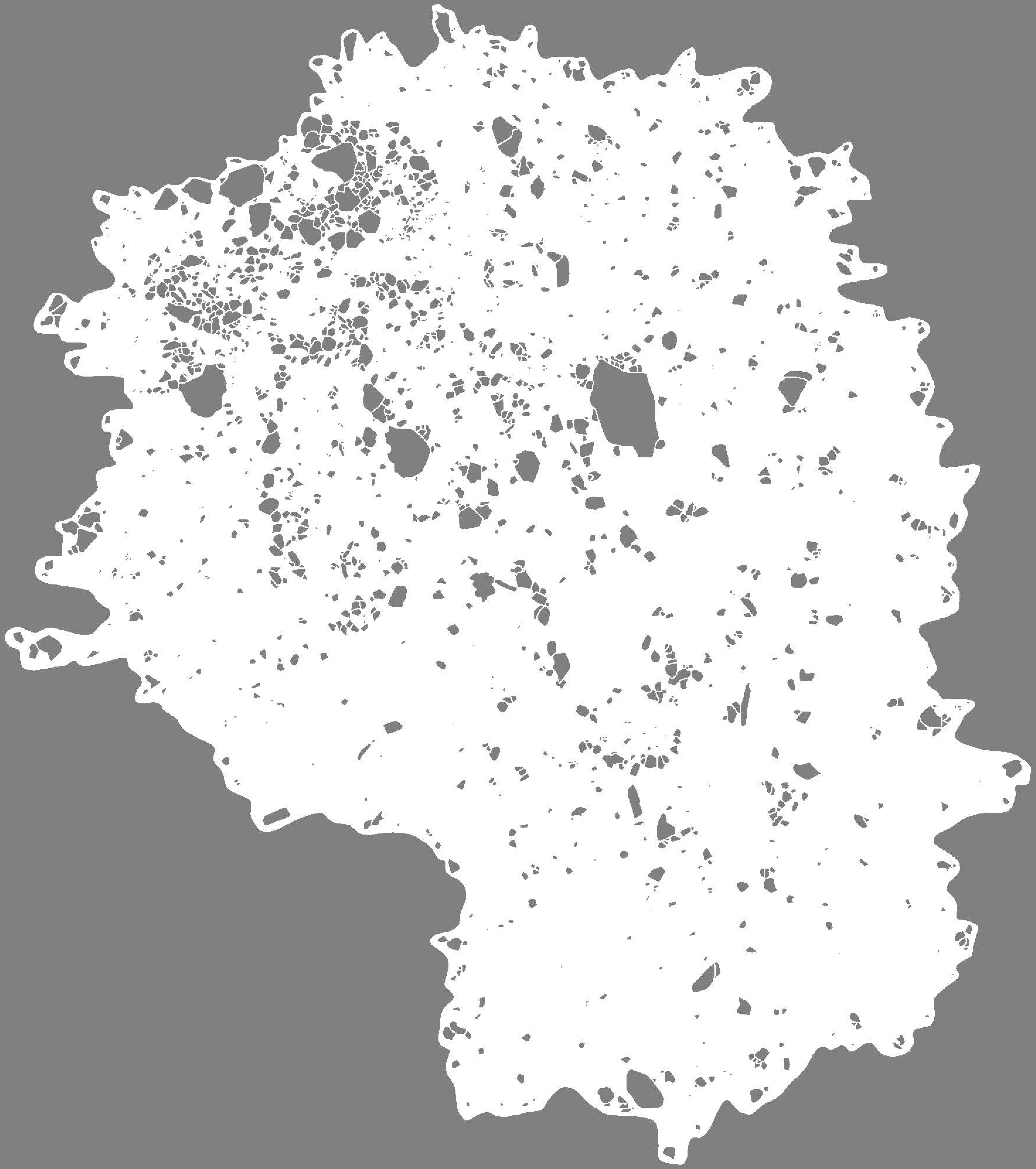

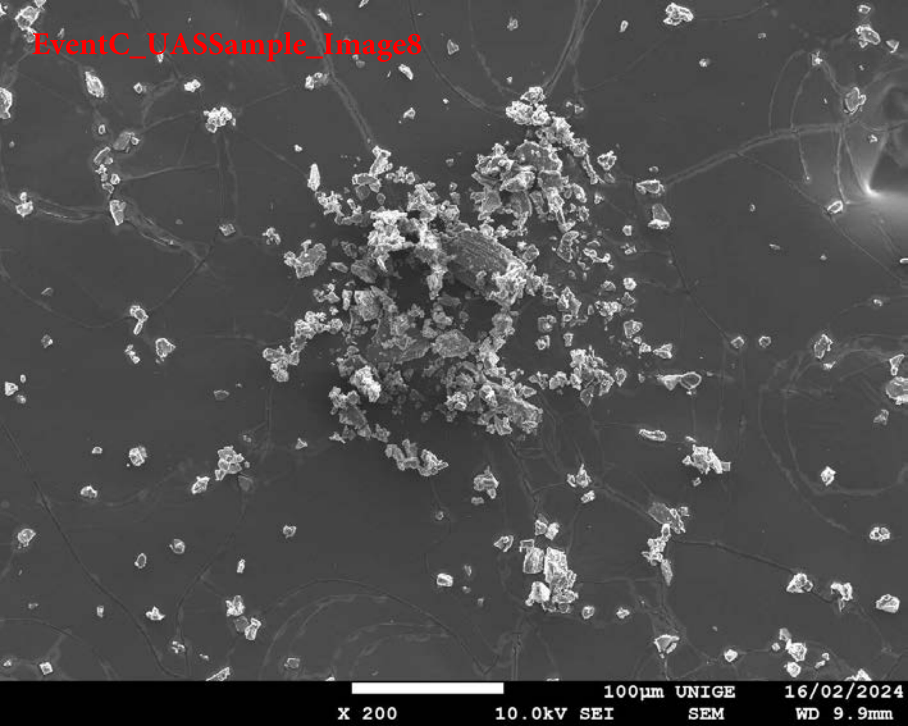
EventC_UASSample_Image8
100µm UNIGE
16/02/2024
X 200
10.0kV SEI
SEM
WD 9.9mm

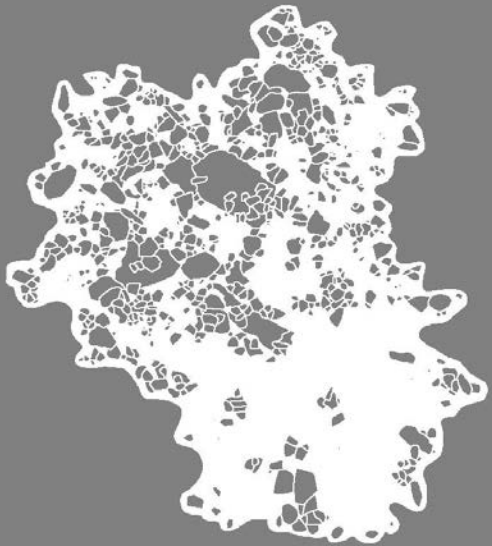

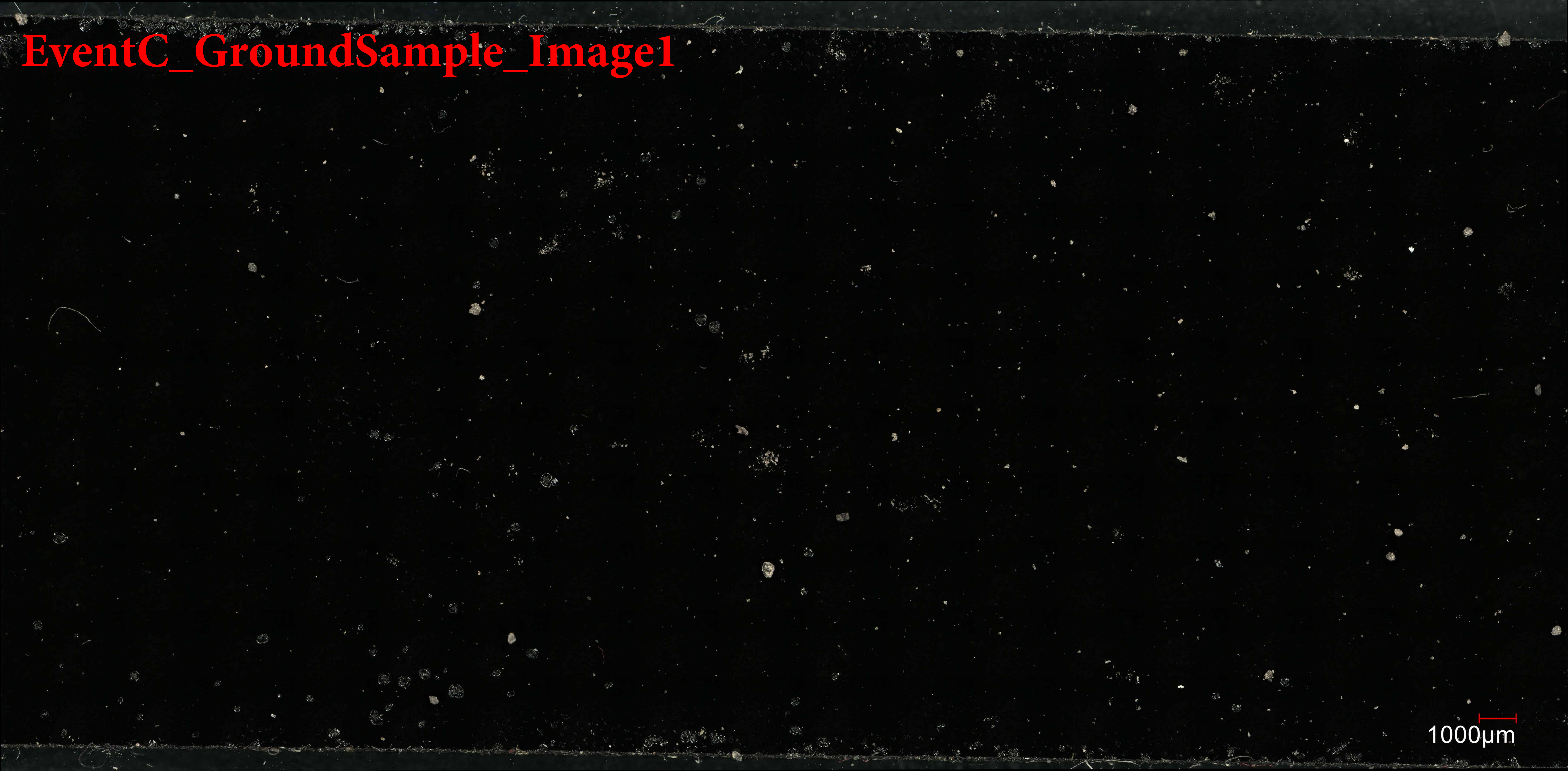
EventC_GroundSample_Image1
1000μm

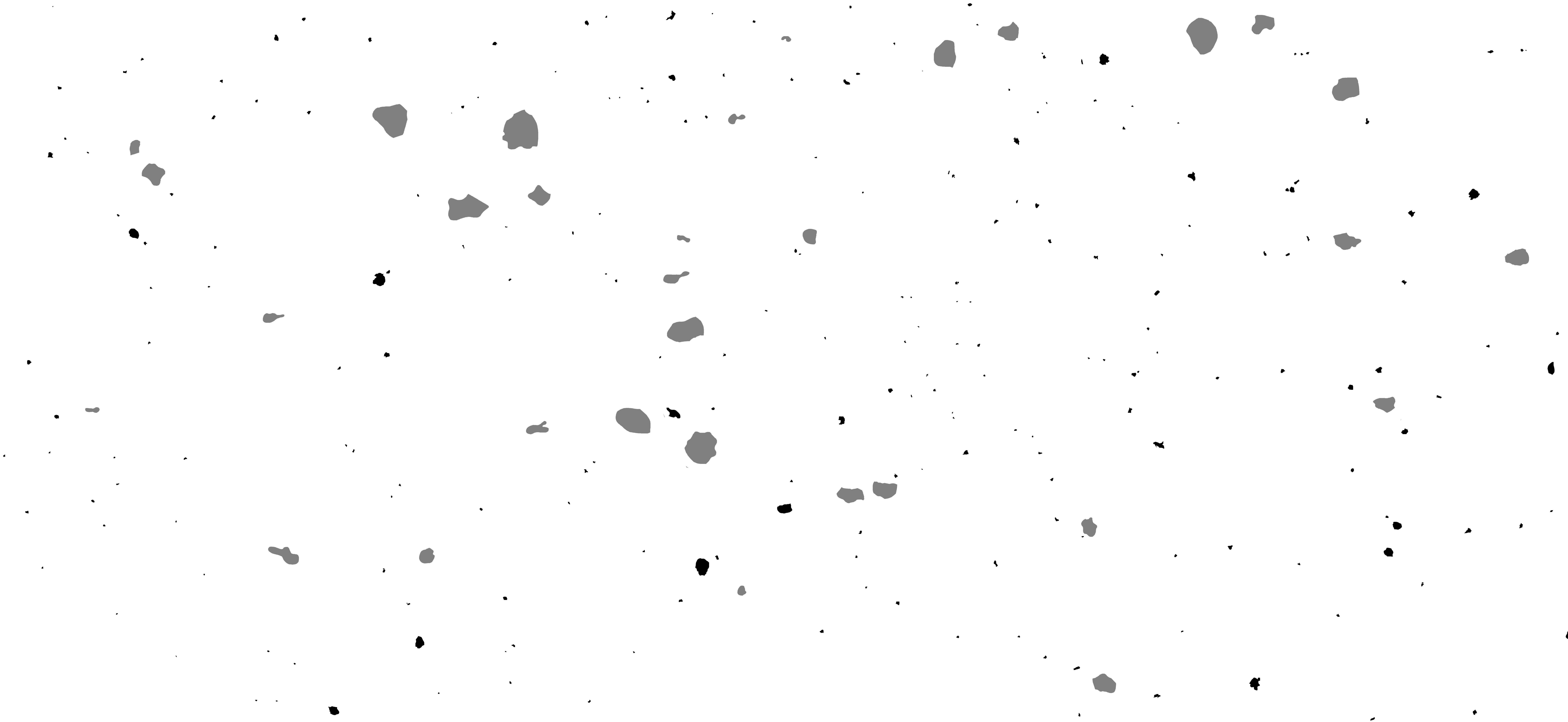

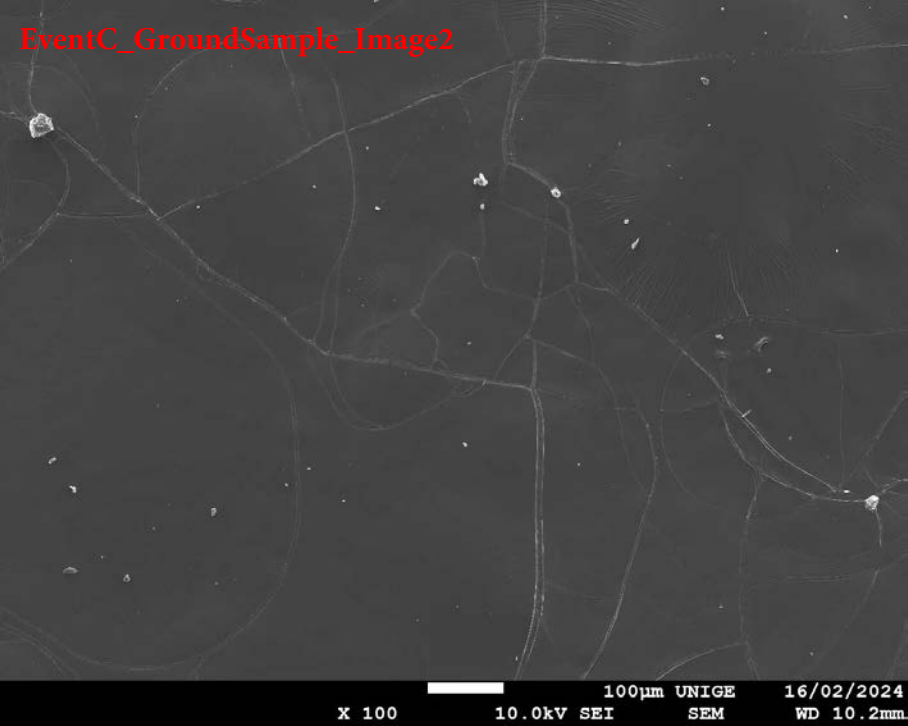
EventC_GroundSample_Image2
100µm UNIGE
16/02/2024
X 100
10.0kV SEI
SEM
WD 10.2mm

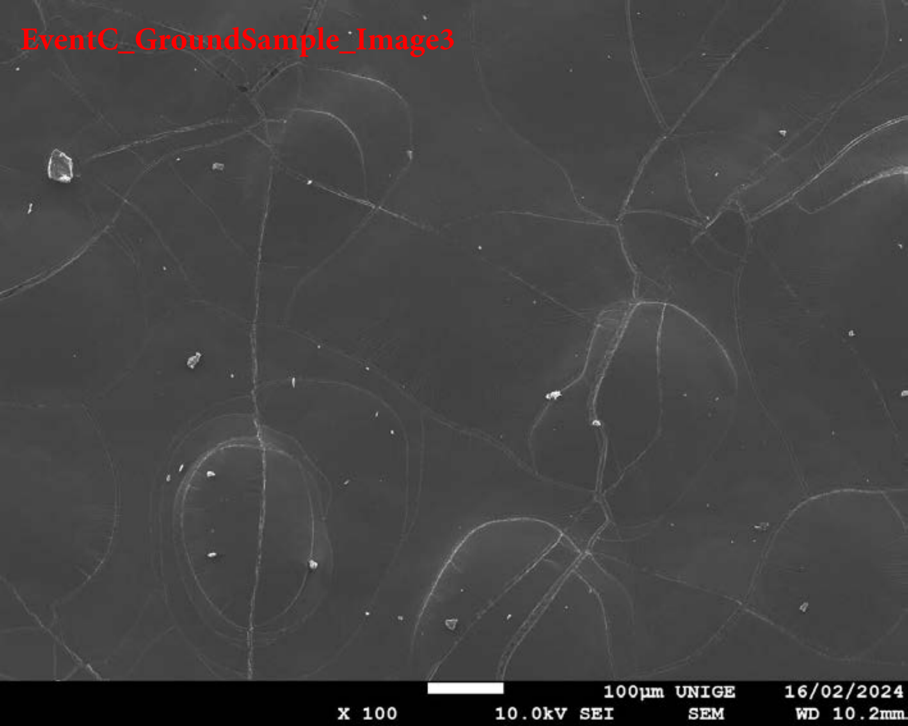
EventC_GroundSample_Image3
100µm UNIGE
16/02/2024
X 100
10.0kV SEI
SEM
WD 10.2mm

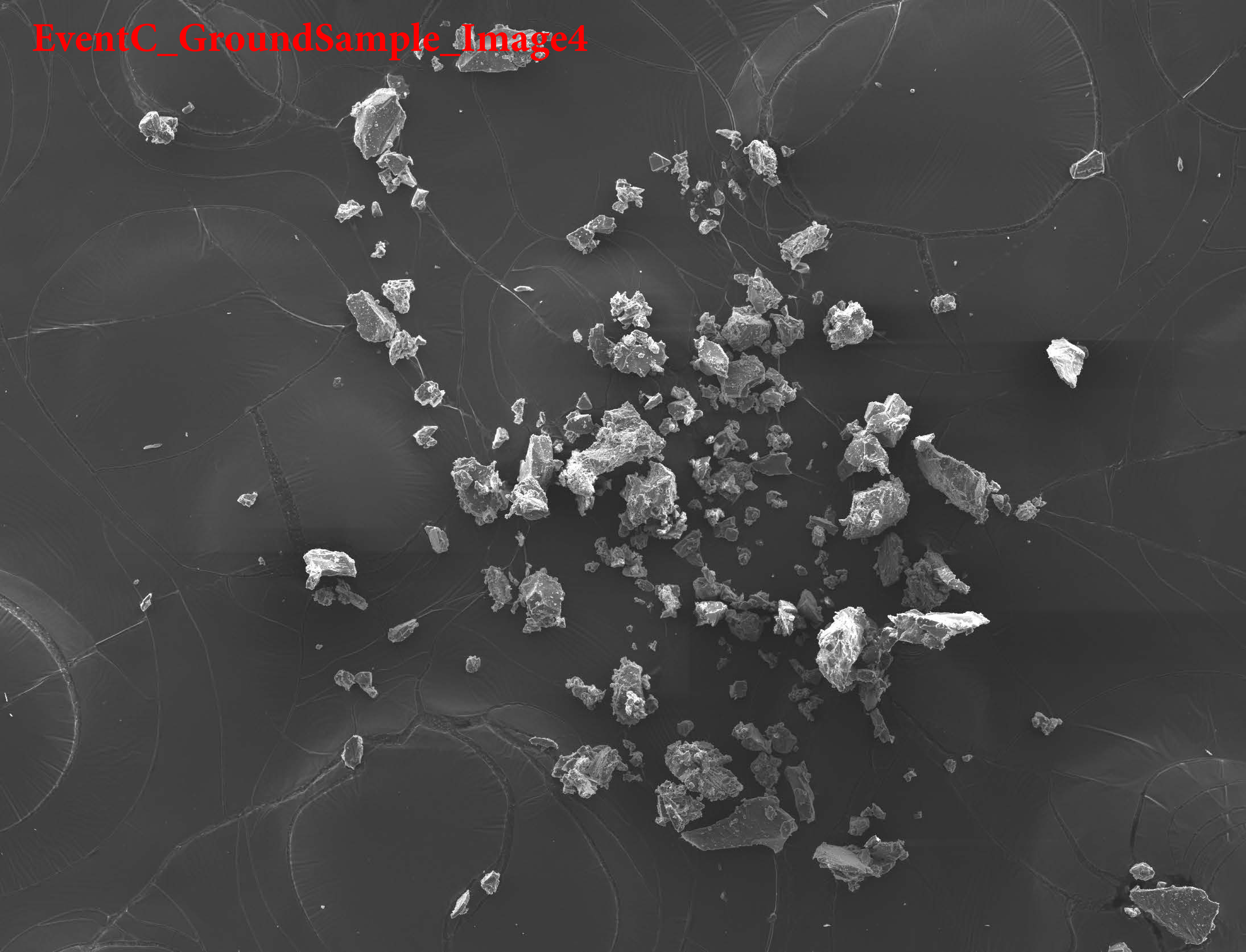
EventC_GroundSample_Image4

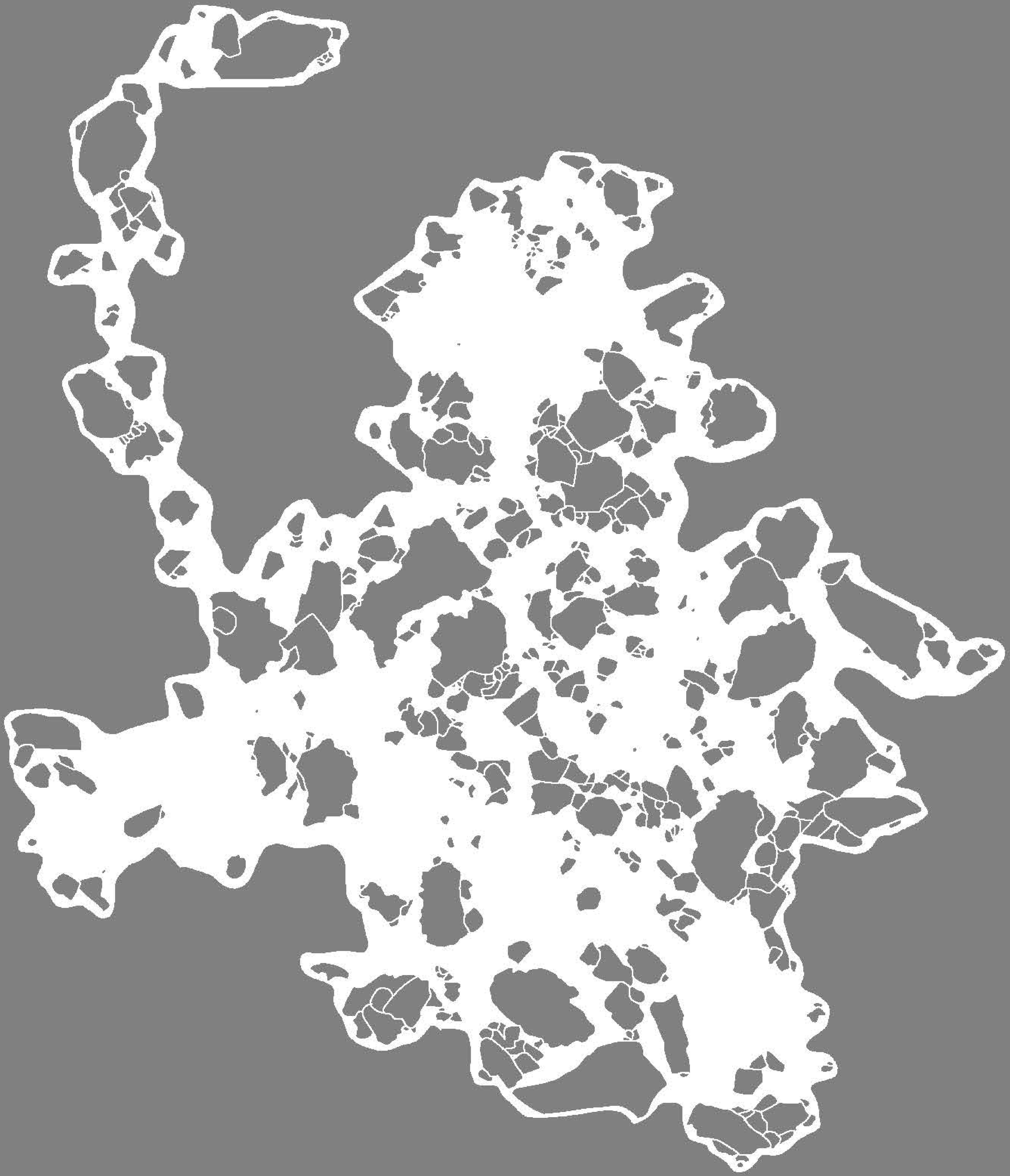

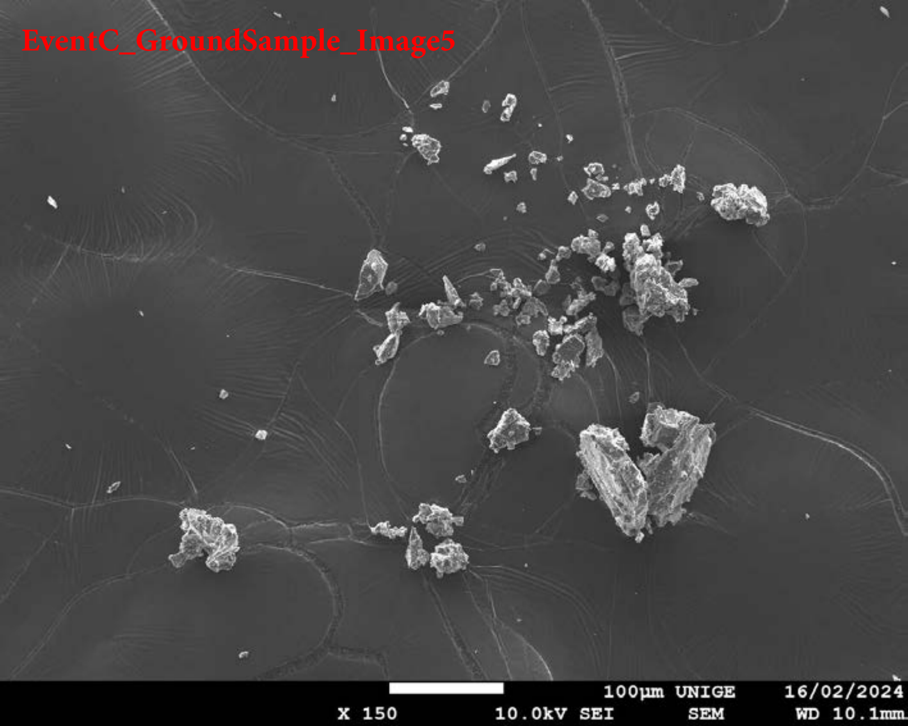
EventC_GroundSample_Image5
100μm UNIGE
16/02/2024
X 150
10.0kV SEI
SEM
WD 10.1mm

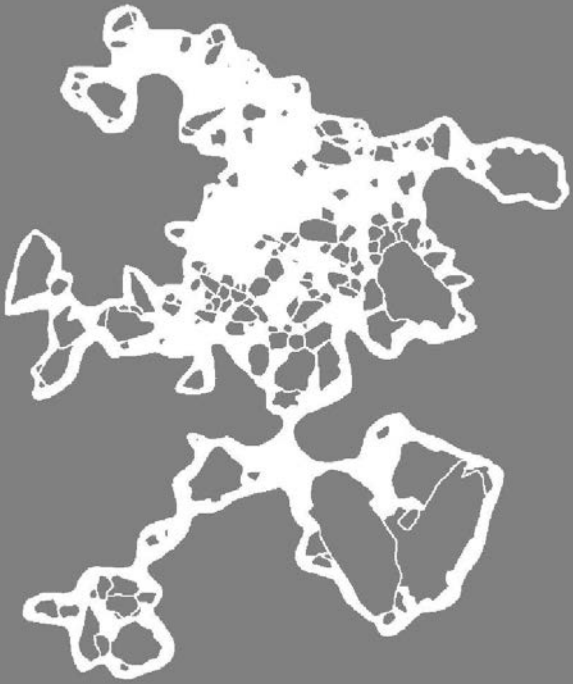

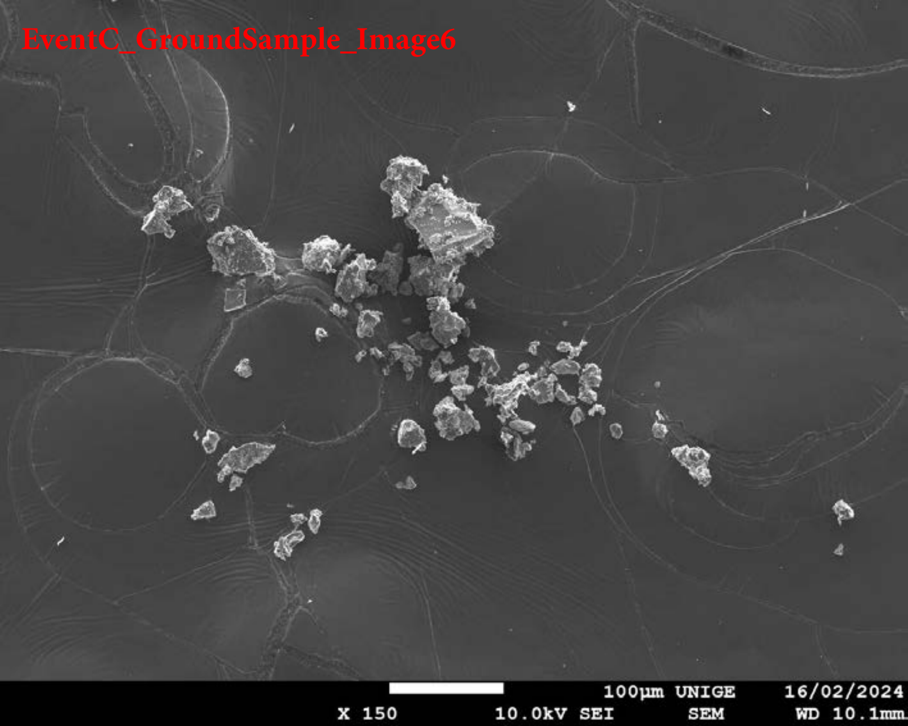
EventC_GroundSample_Image6
100µm UNIGE
16/02/2024
X 150
10.0kV SEI
SEM
WD 10.1mm

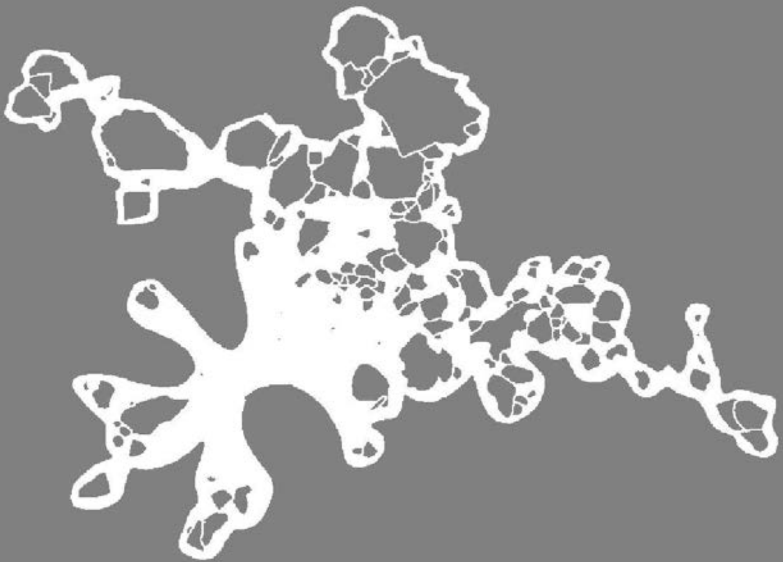

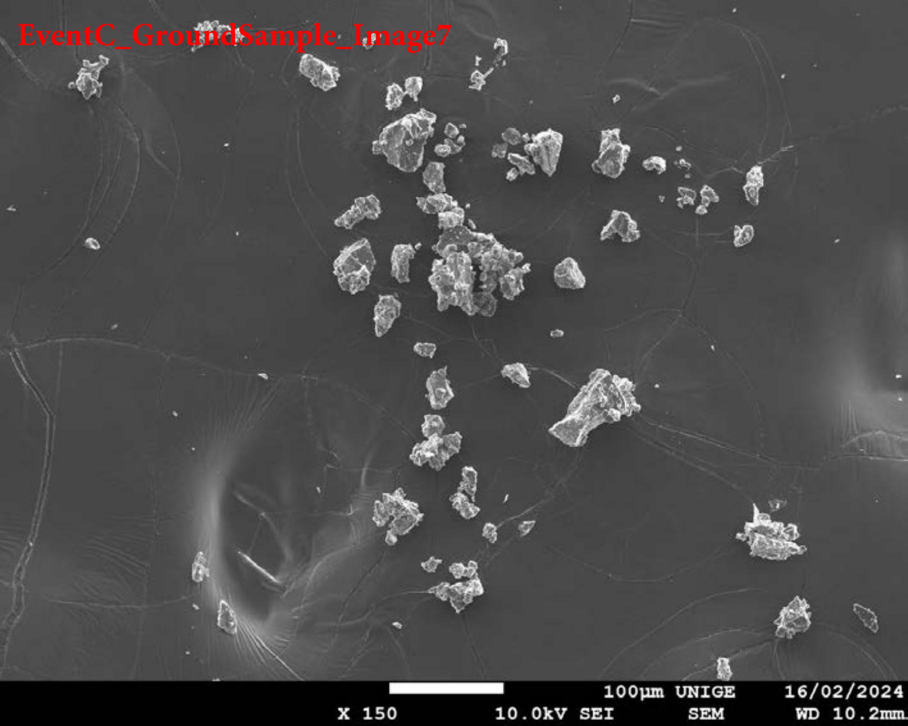
EventC_GroundSample_Image7
100µm UNIGE
16/02/2024
X 150
10.0kV SEI
SEM
WD 10.2mm

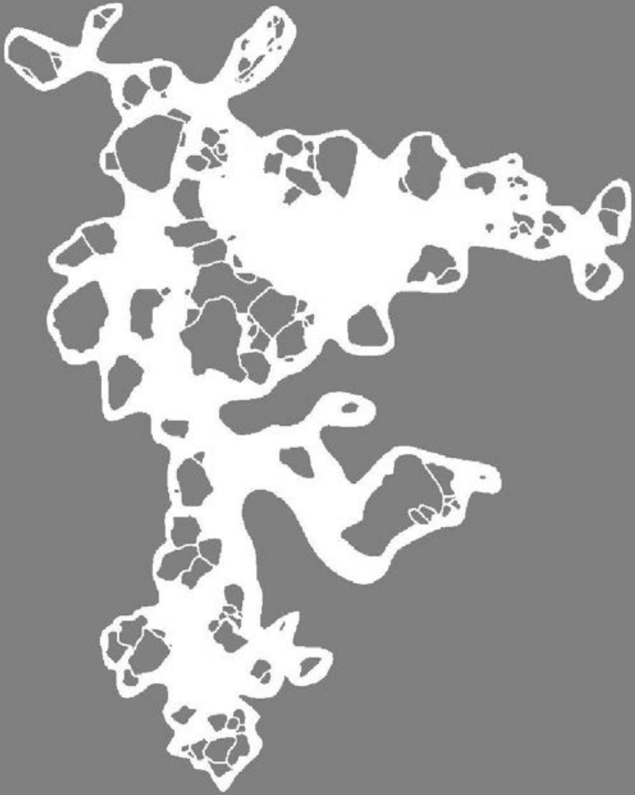

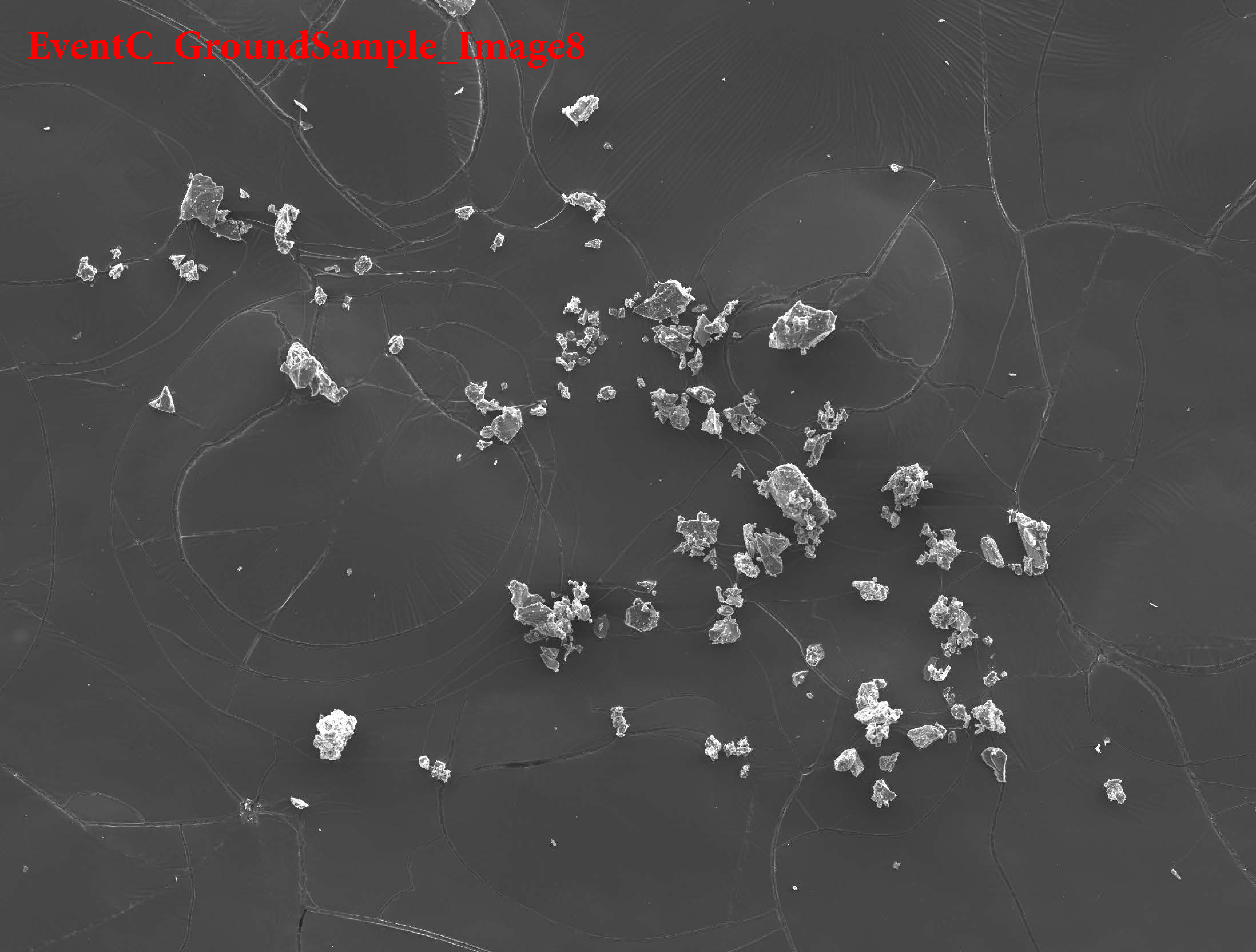
EventC_GroundSample_Image8

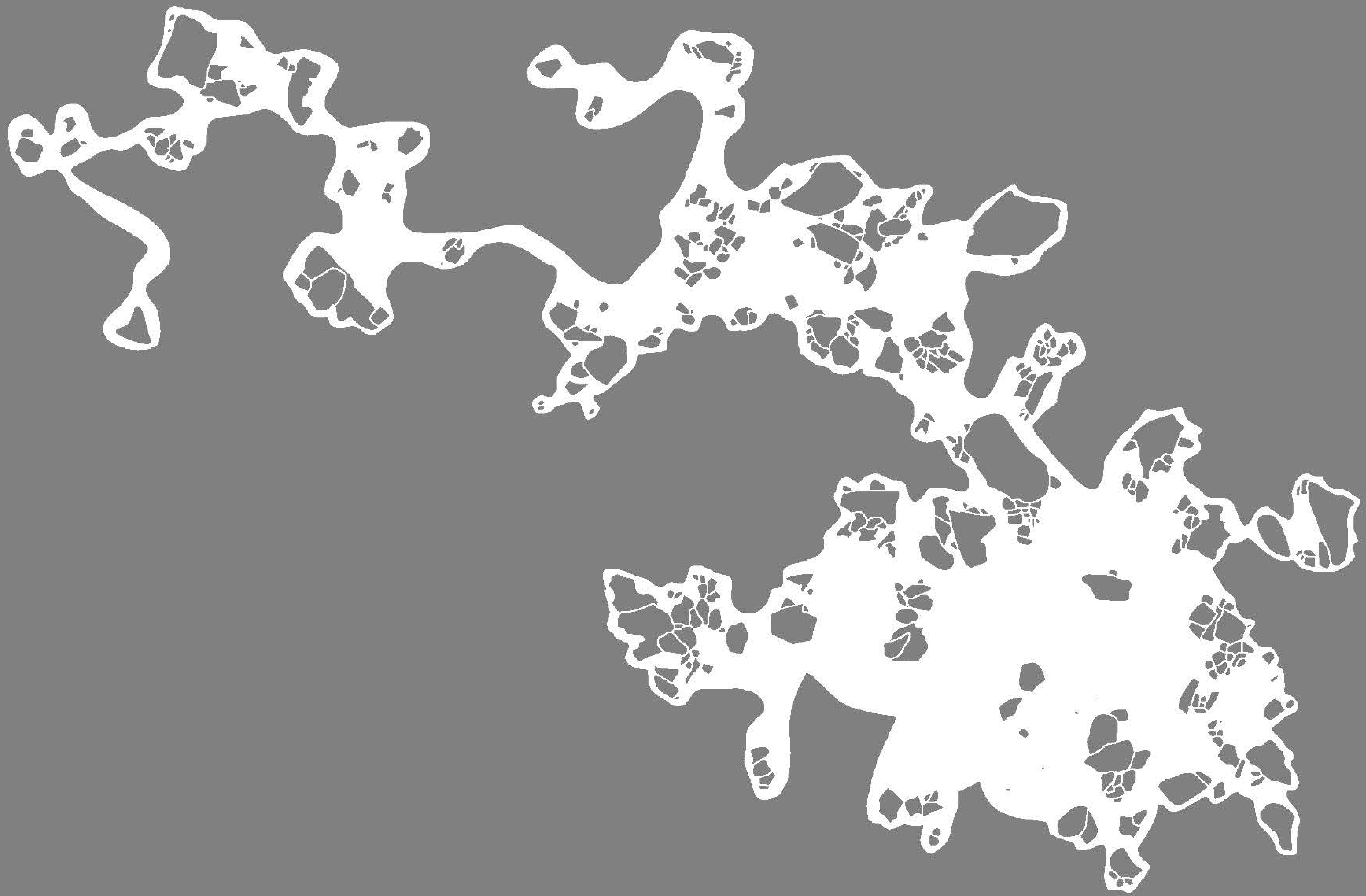

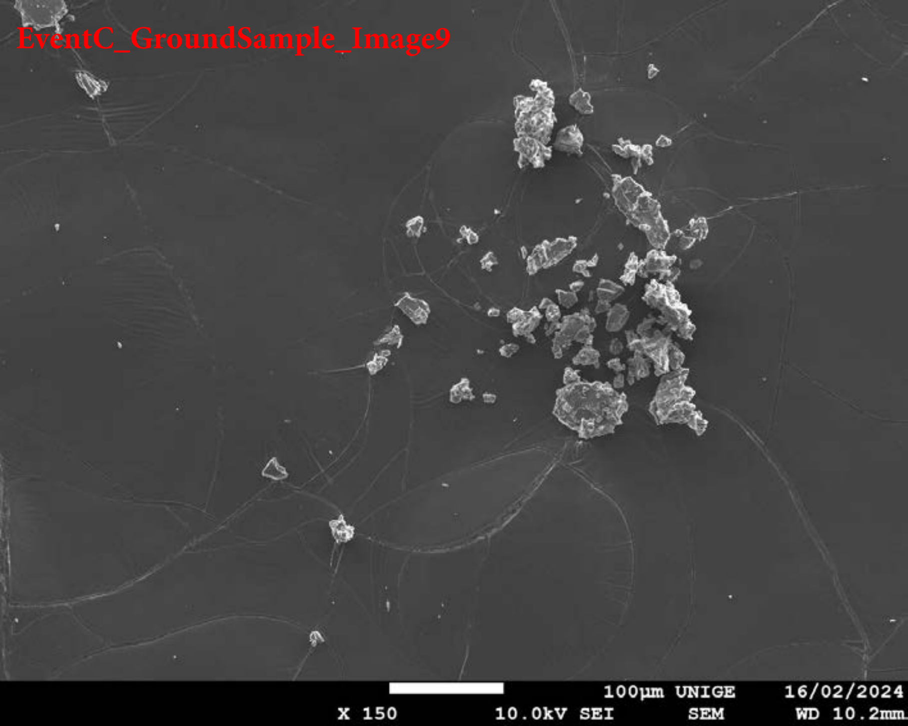
EventC_GroundSample_Image9
100µm UNIGE
16/02/2024
X 150
10.0kV SEI
SEM
WD 10.2mm

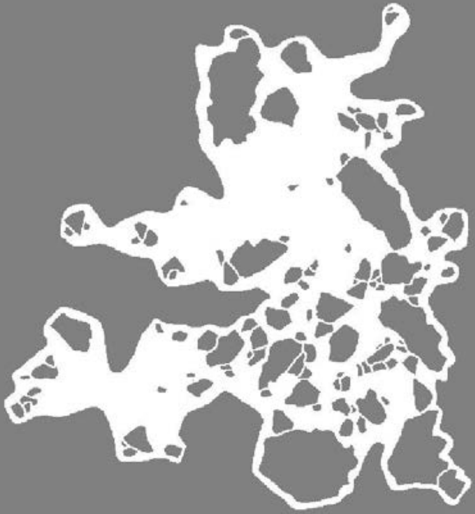

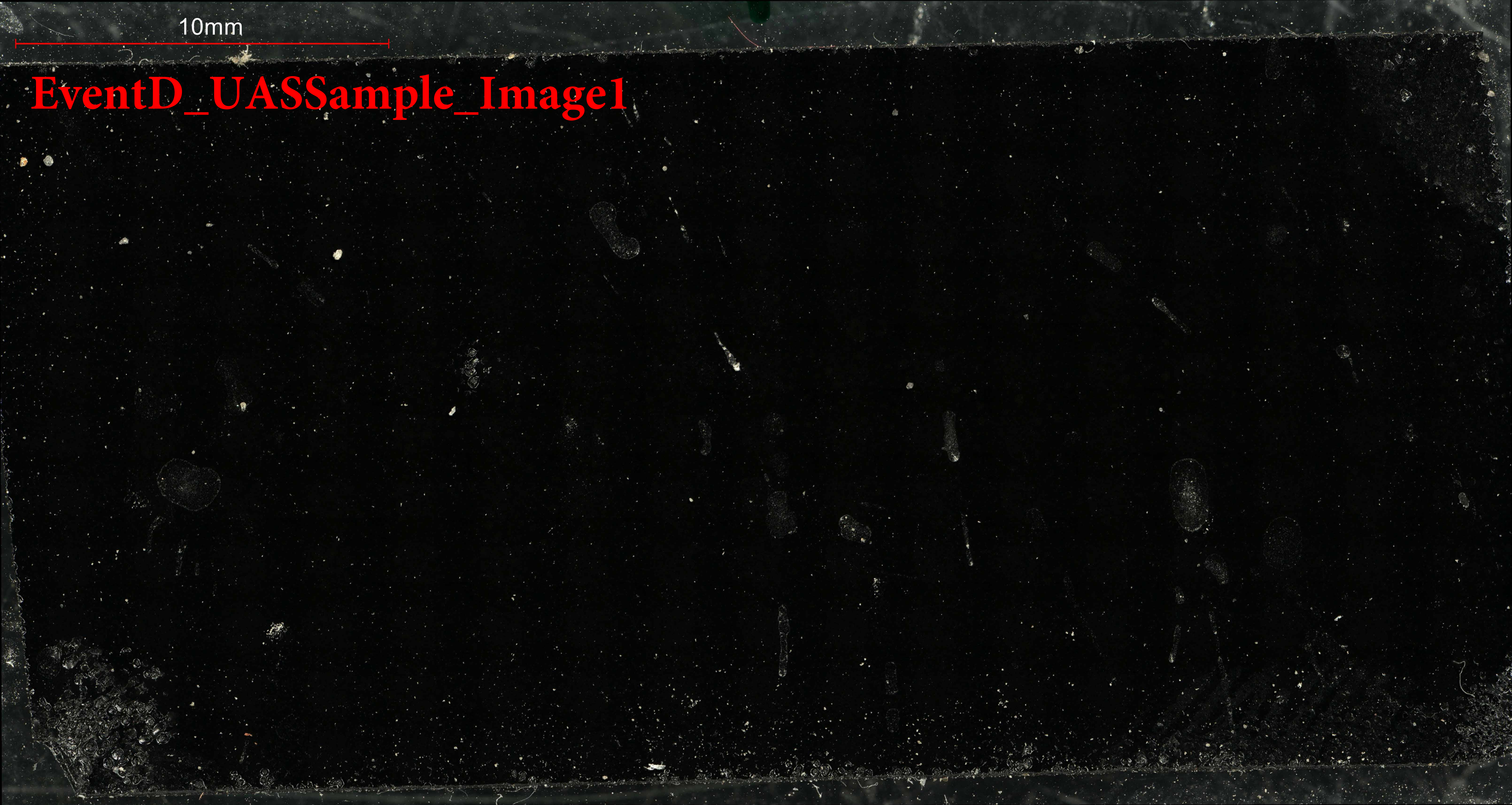
10mm
EventD_UASSample_Image1

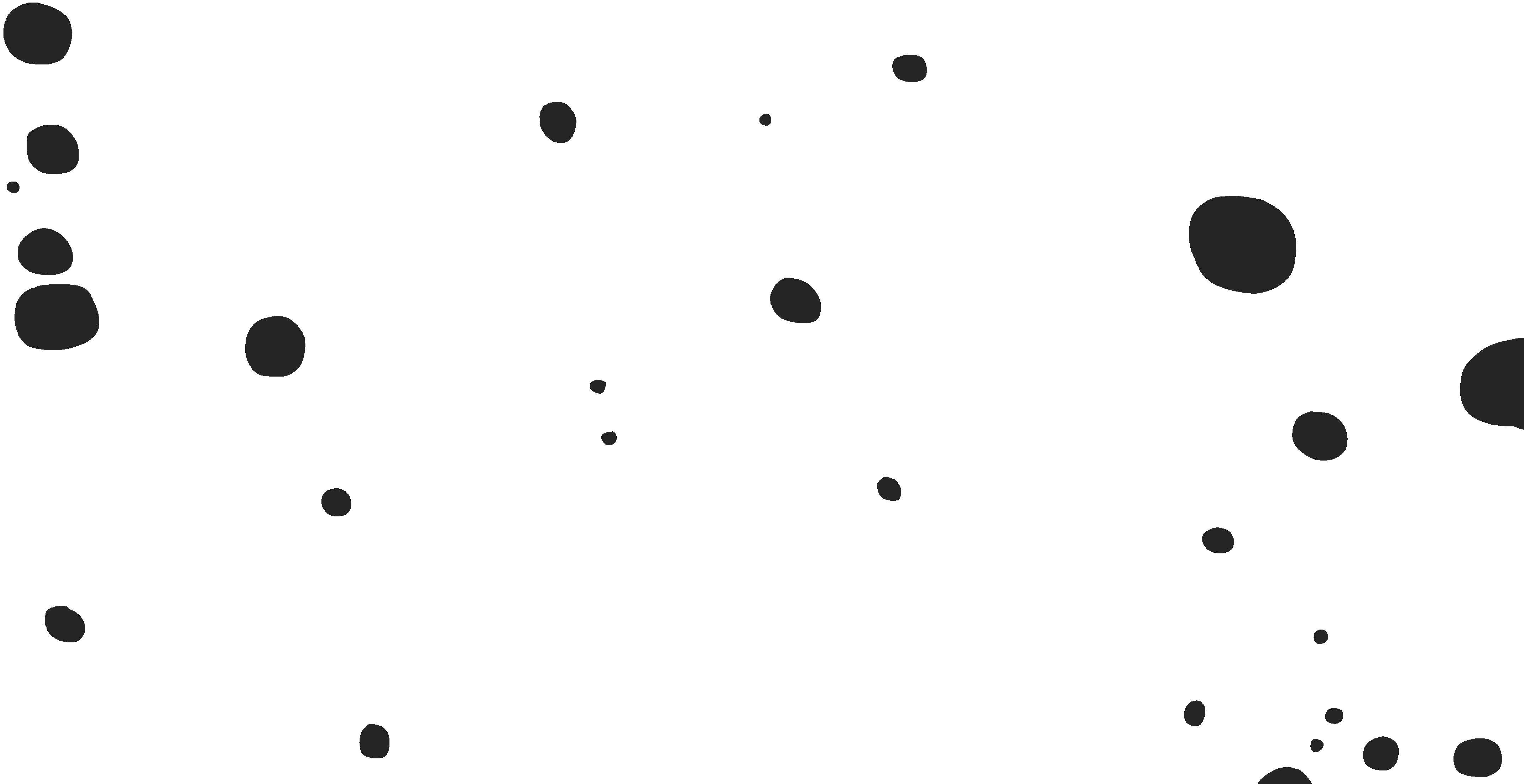

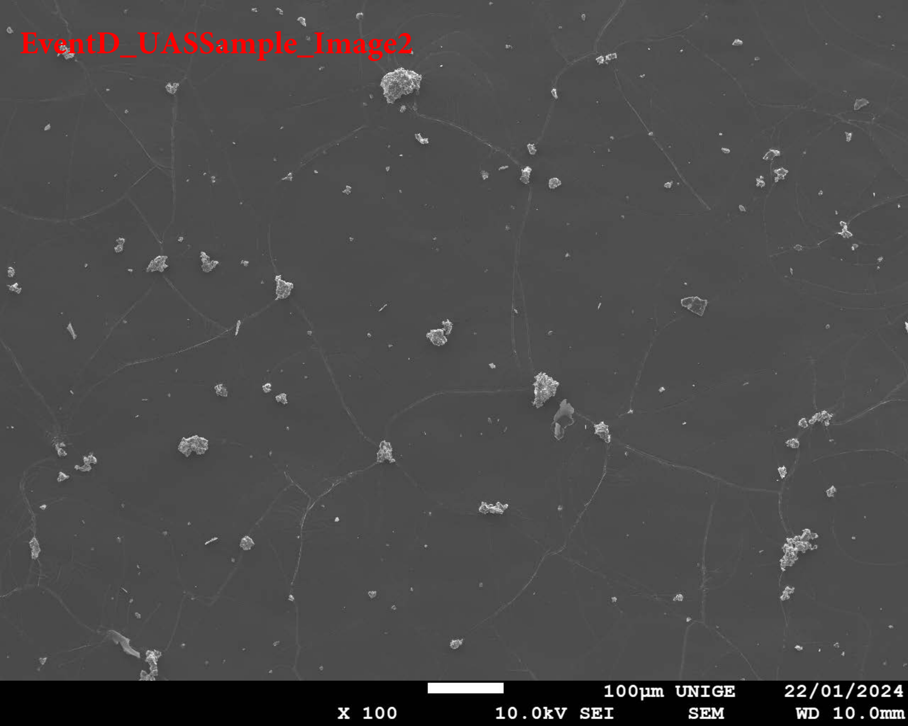
EventD_UASSample_Image2
100μm UNIGE
22/01/2024
X 100
10.0kV SEI
SEM
WD 10.0mm

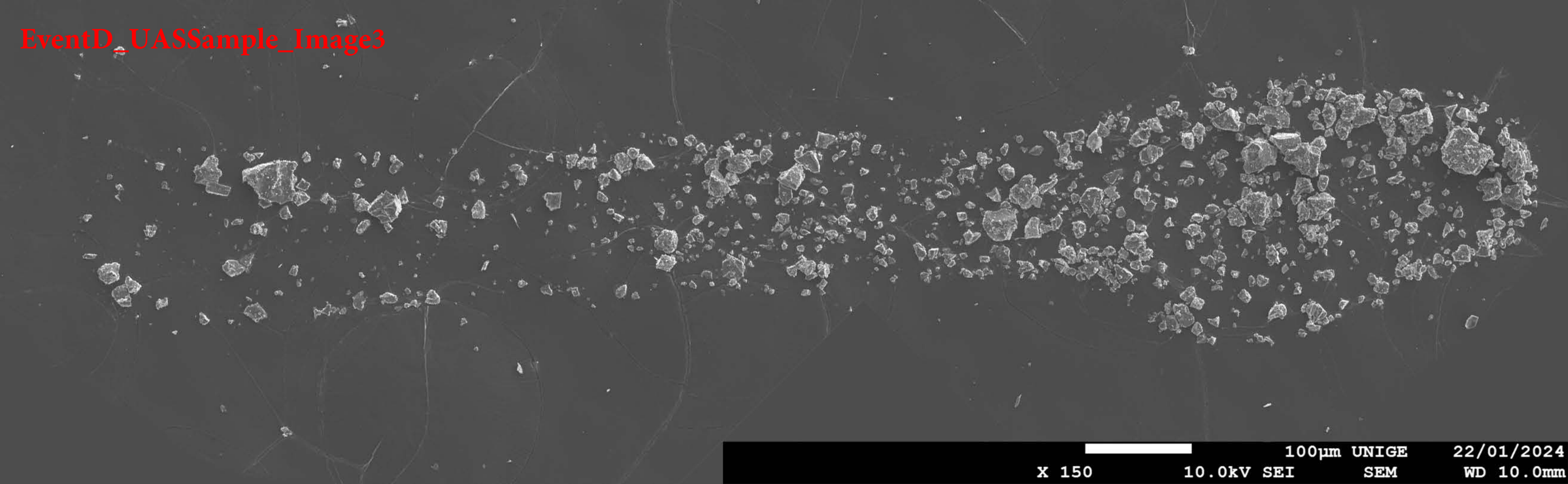
EventD_UASSample_Image3
100µm UNIGE
22/01/2024
X 150
10.0kV SEI
SEM
WD 10.0mm

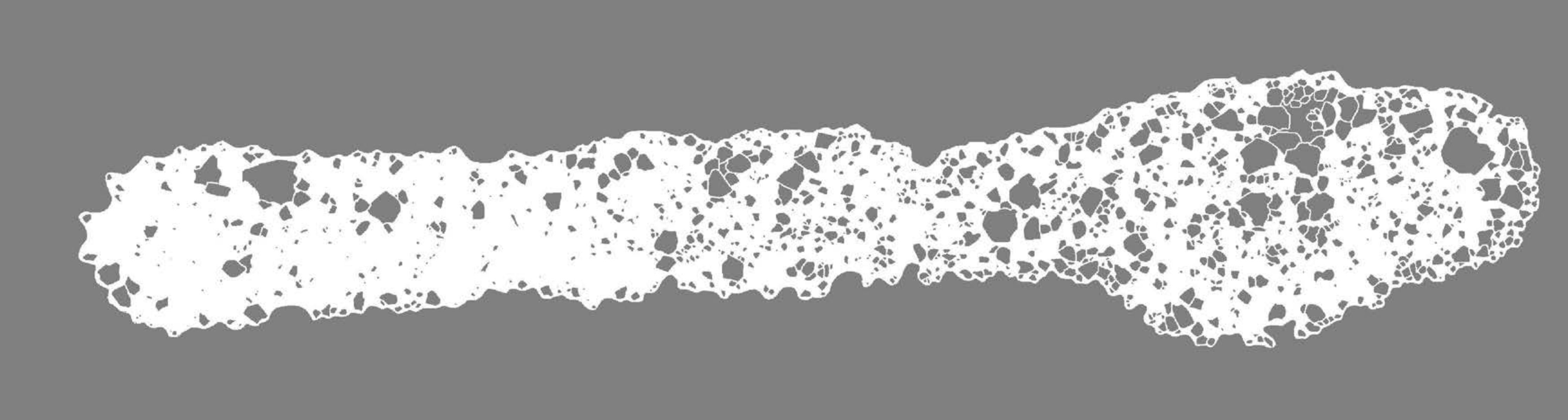

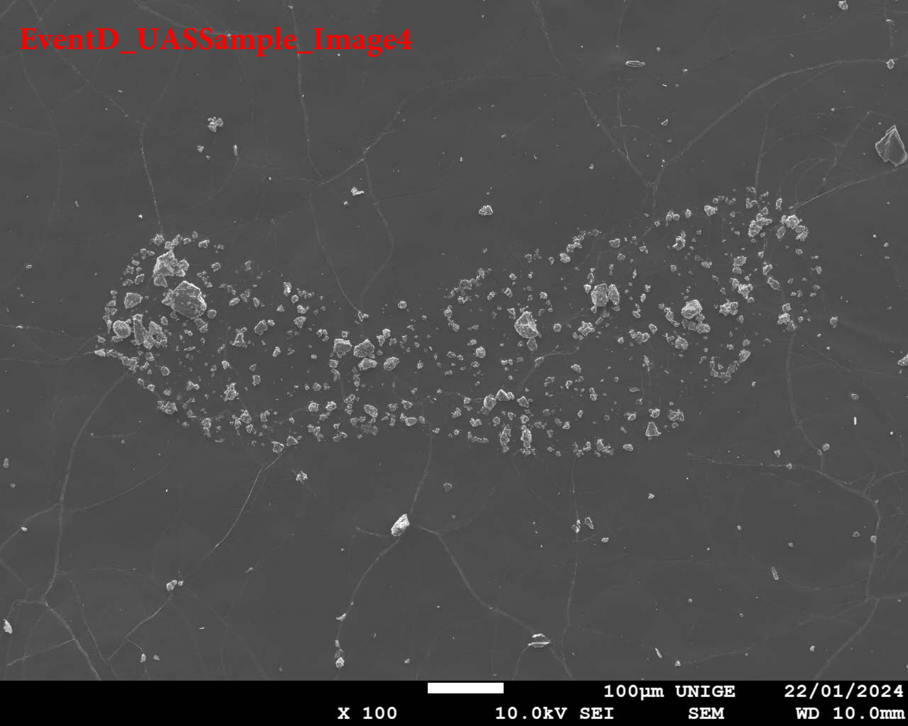
EventD_UASSample_Image4
100µm UNIGE
22/01/2024
X 100
10.0kV SEI
SEM
WD 10.0mm

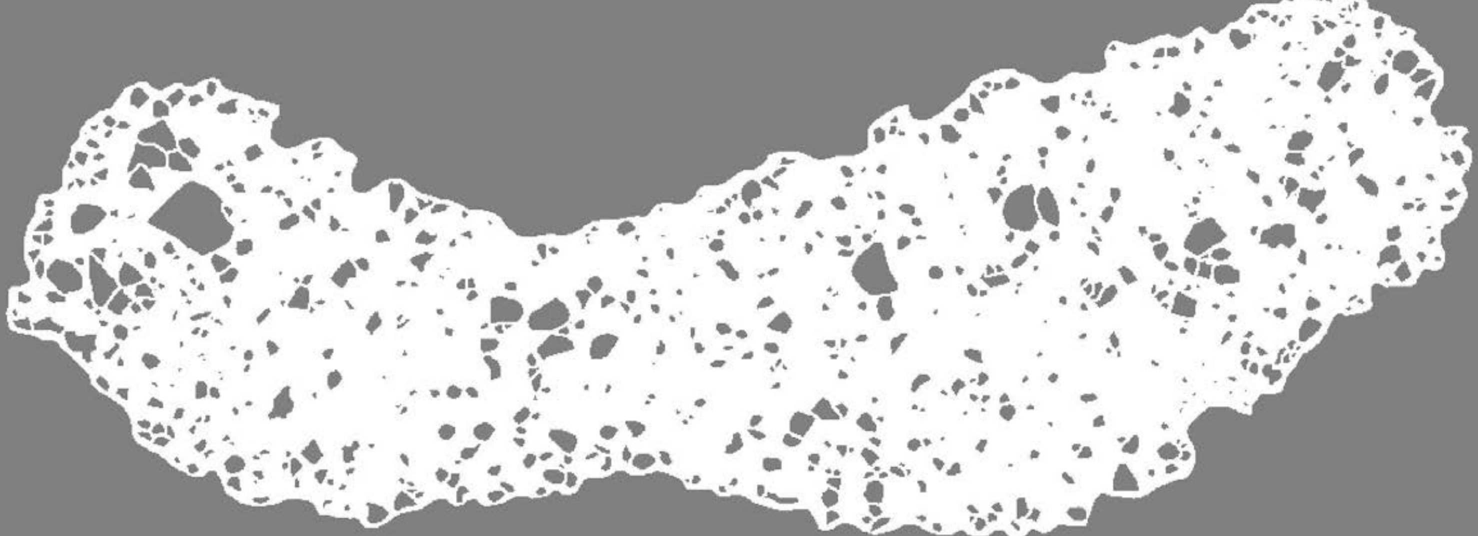

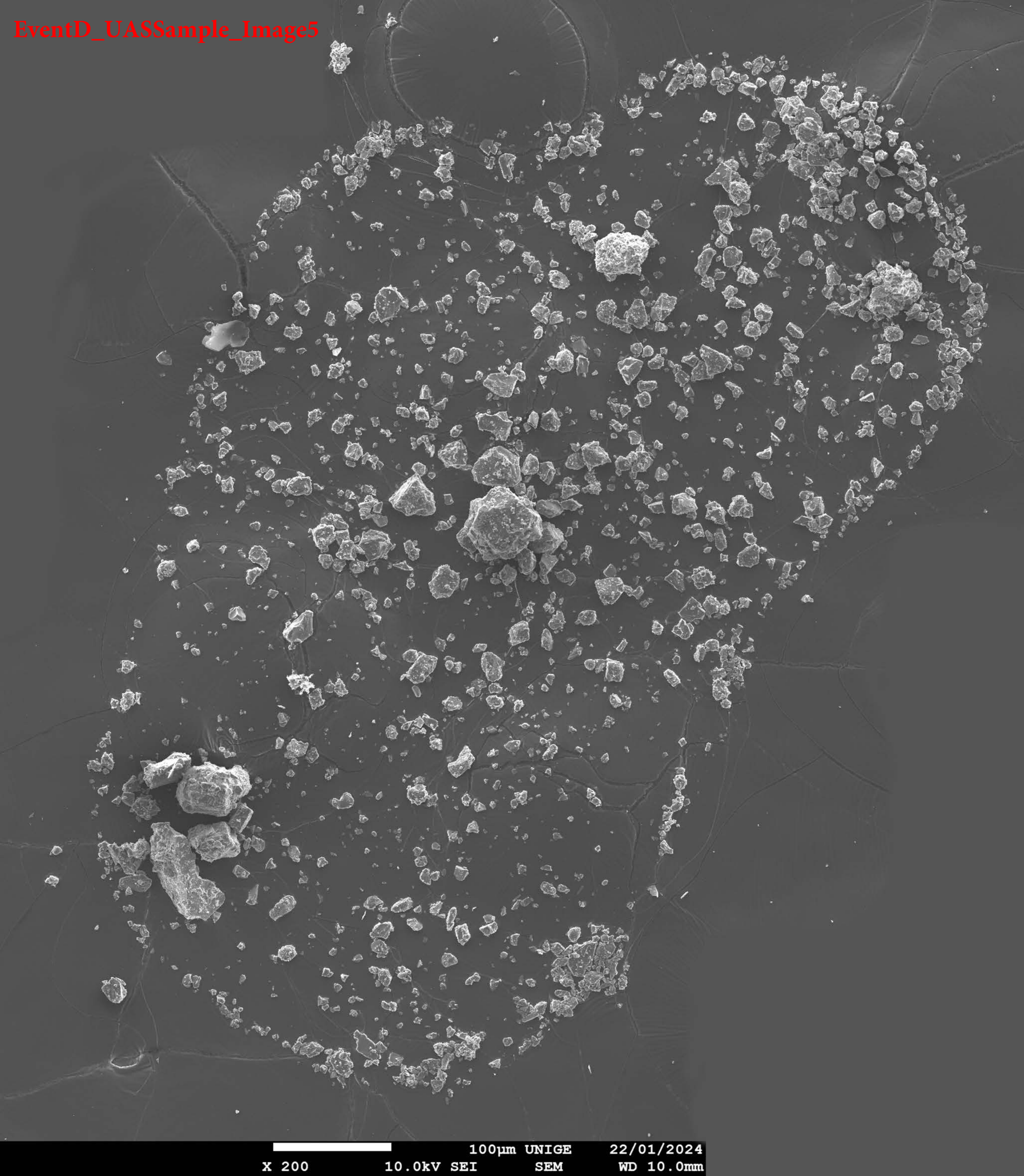
EventD_UASSample_Image5
100µm UNIGE 22/01/2024
X 200 10.0kV SEI SEM WD 10.0mm

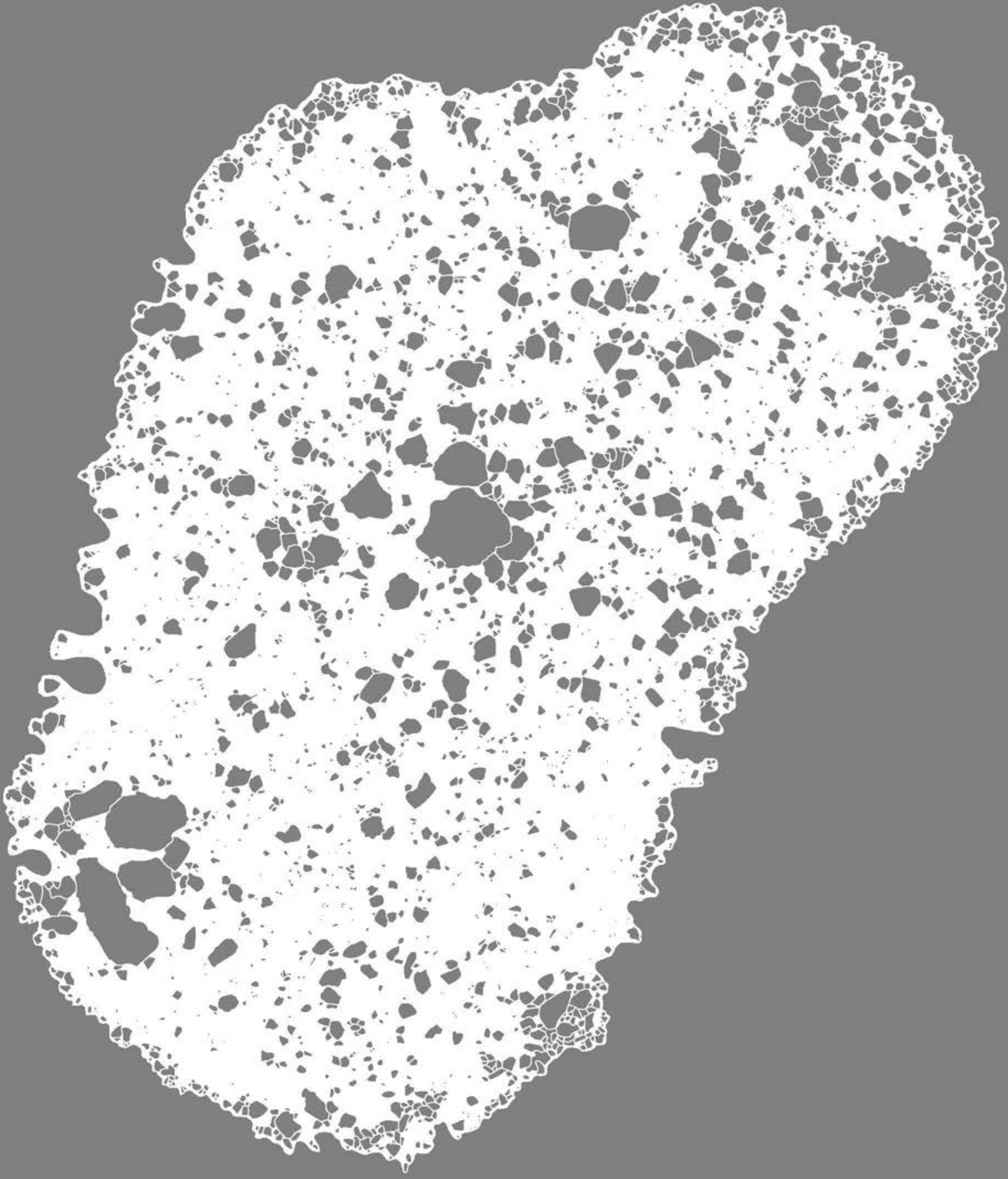

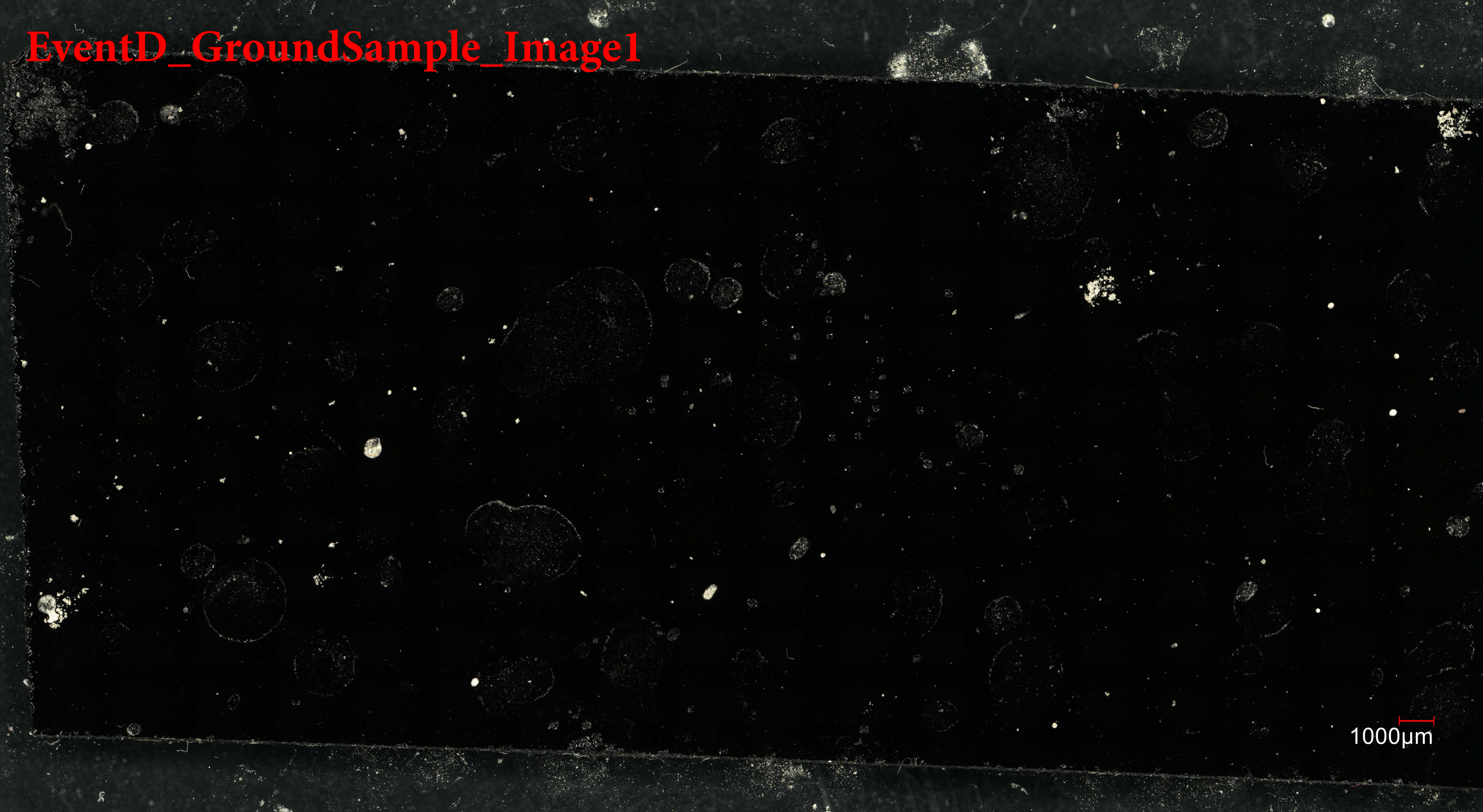
EventD_GroundSample_Image1
1000μm

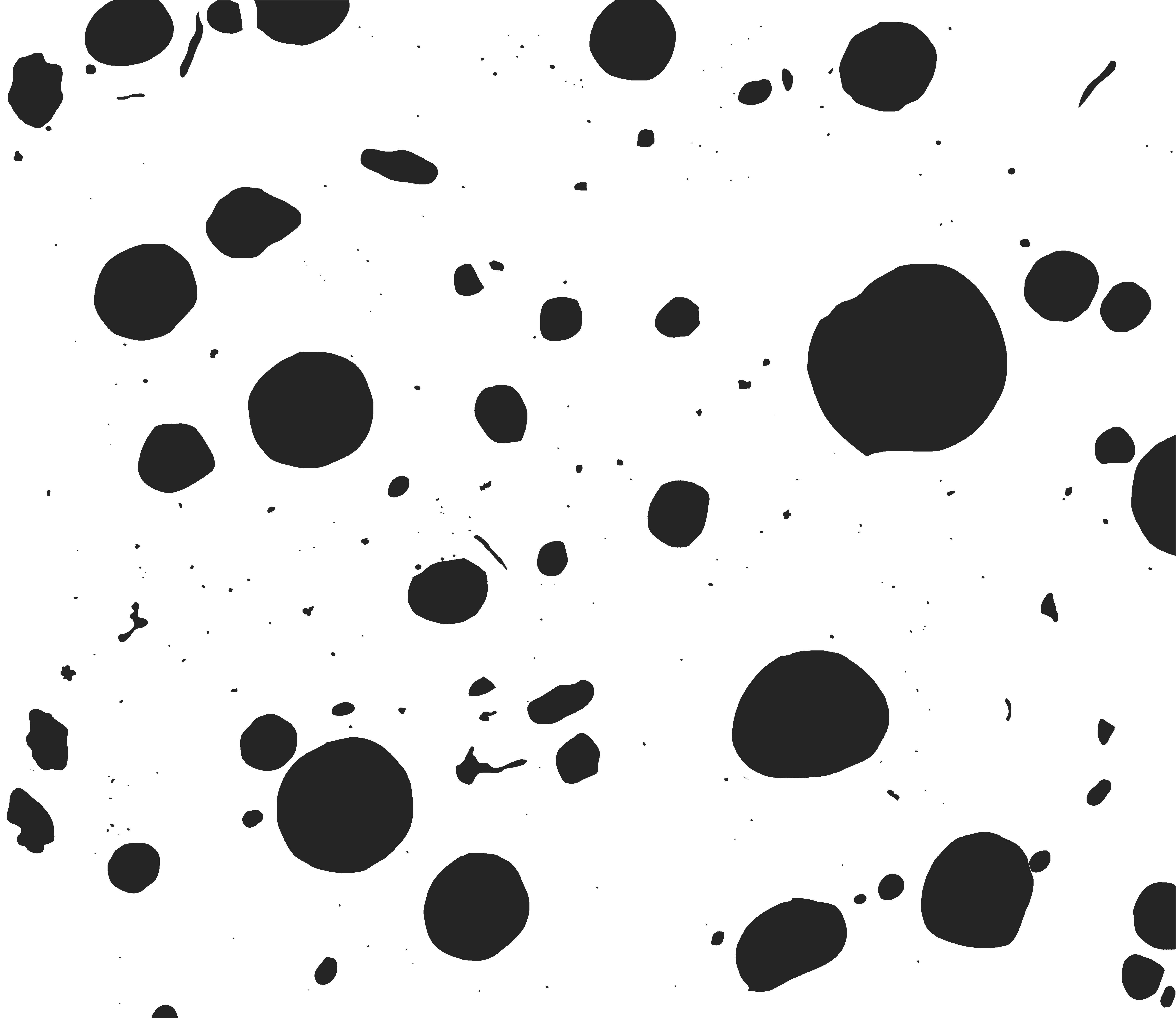

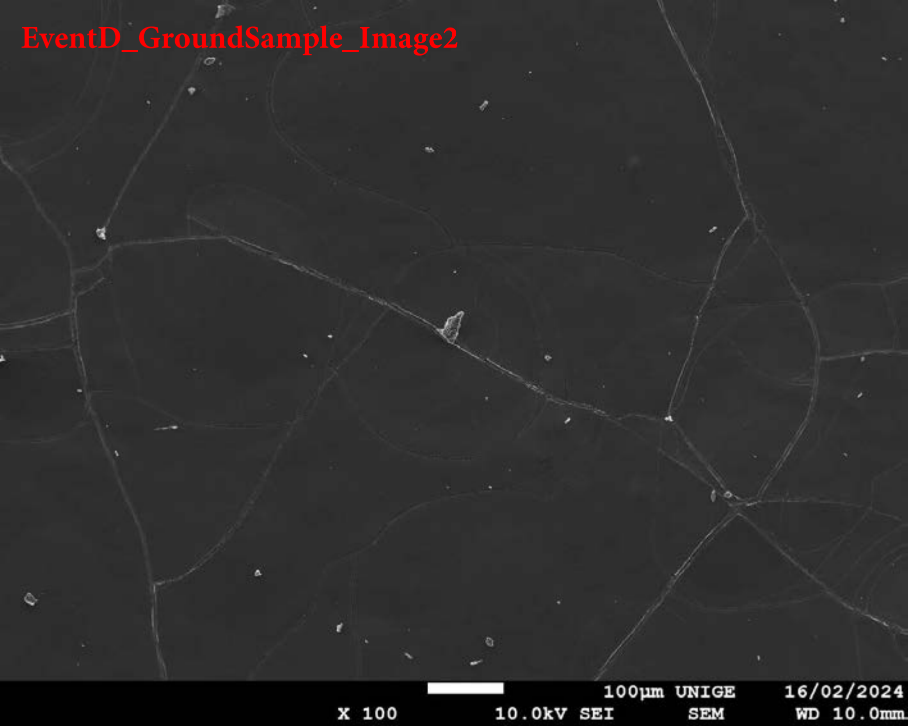
EventD_GroundSample_Image2
100µm UNIGE
16/02/2024
X 100
10.0kV SEI
SEM
WD 10.0mm

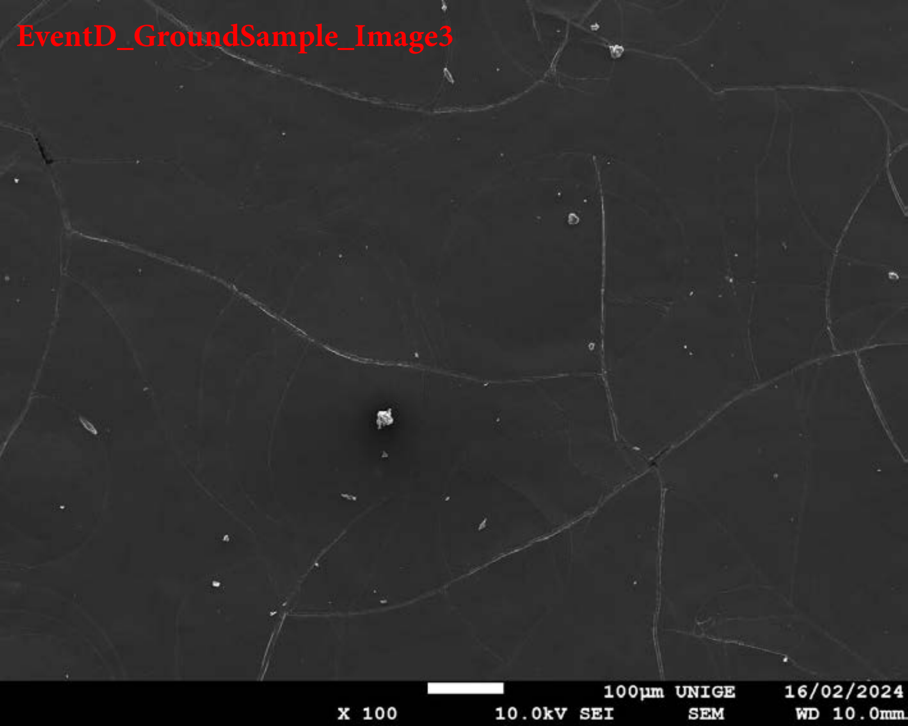
EventD_GroundSample_Image3
100µm UNIGE
16/02/2024
X 100
10.0kV SEI
SEM
WD 10.0mm

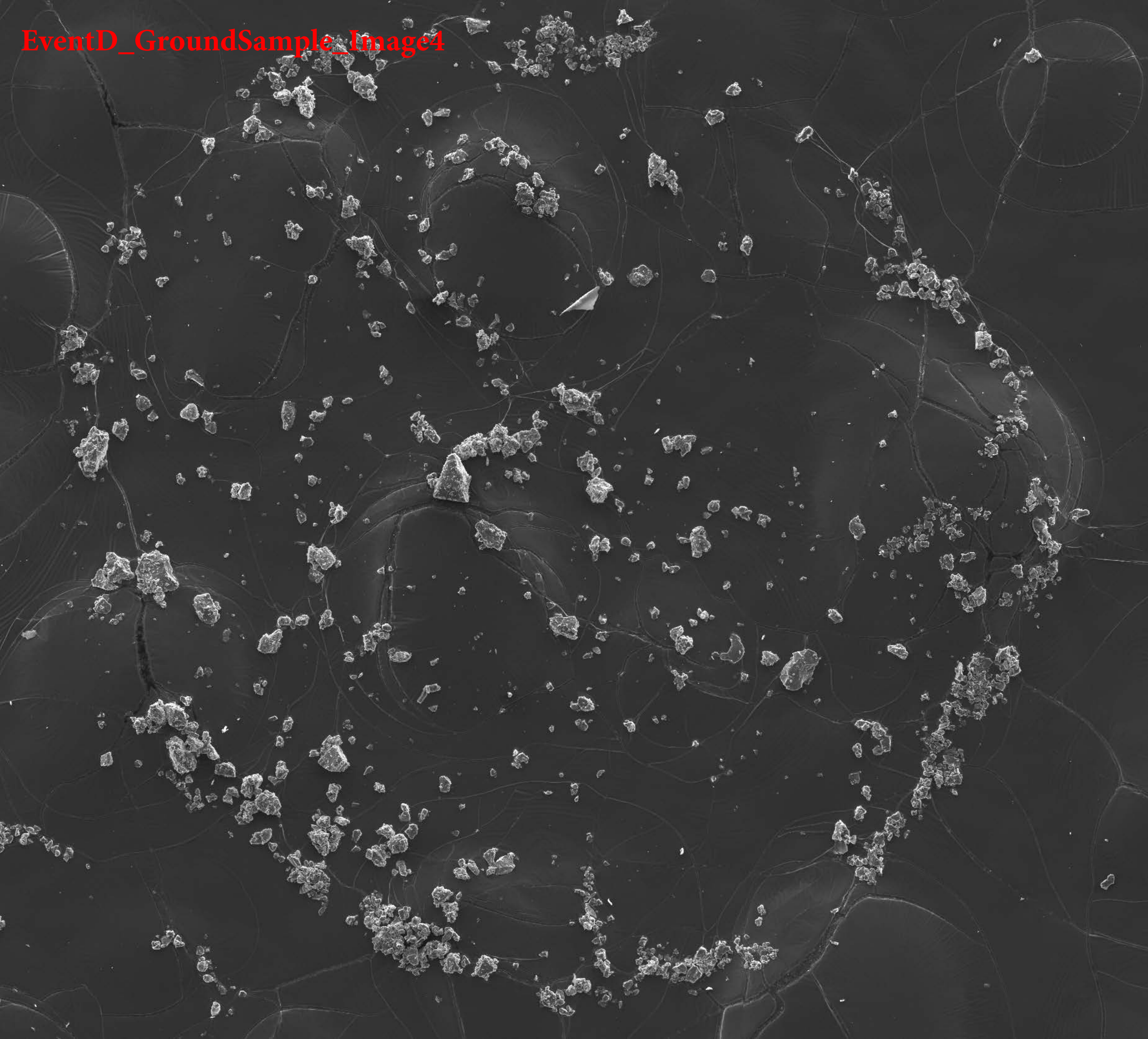
EventD_GroundSample_Image4

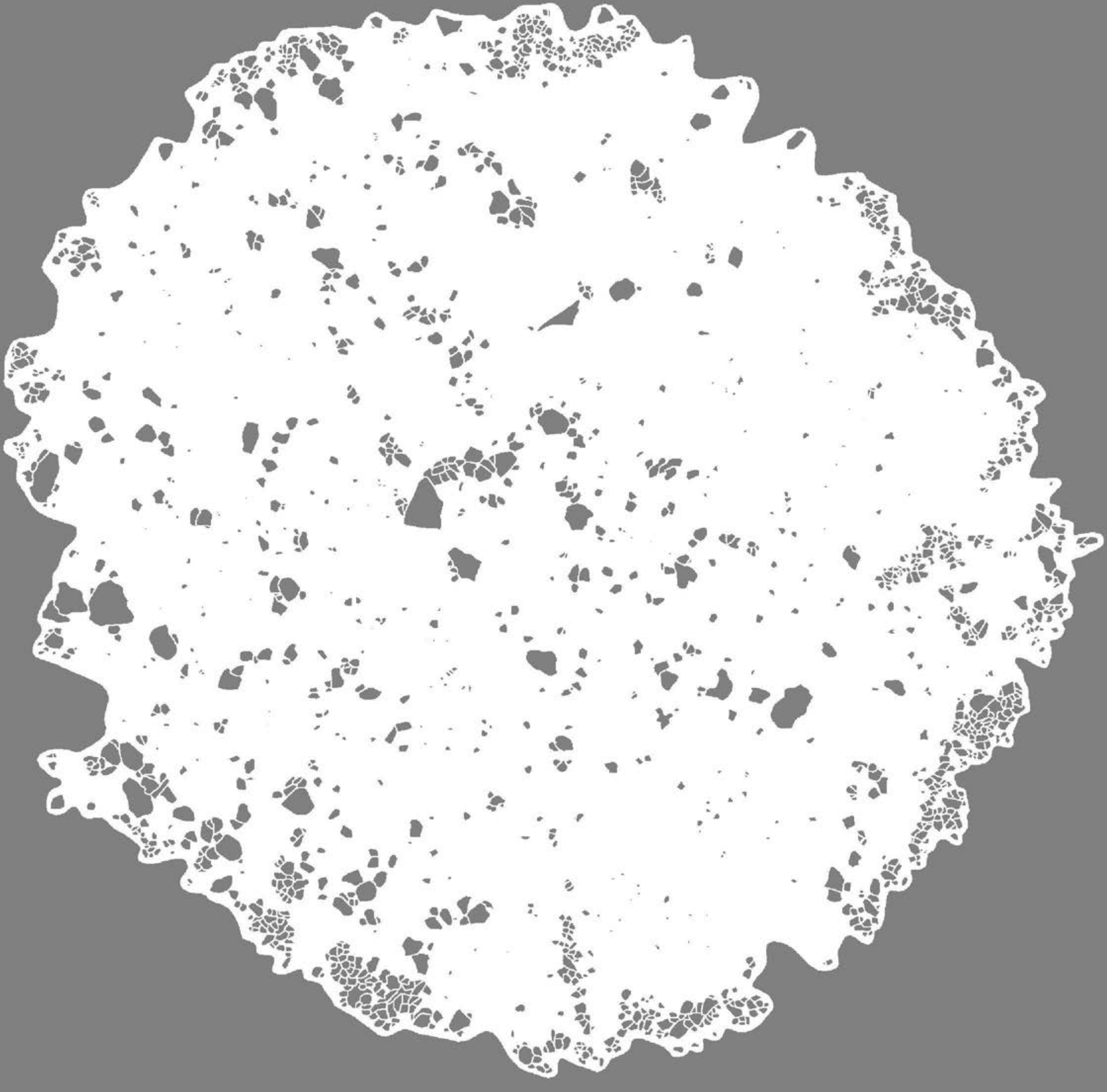

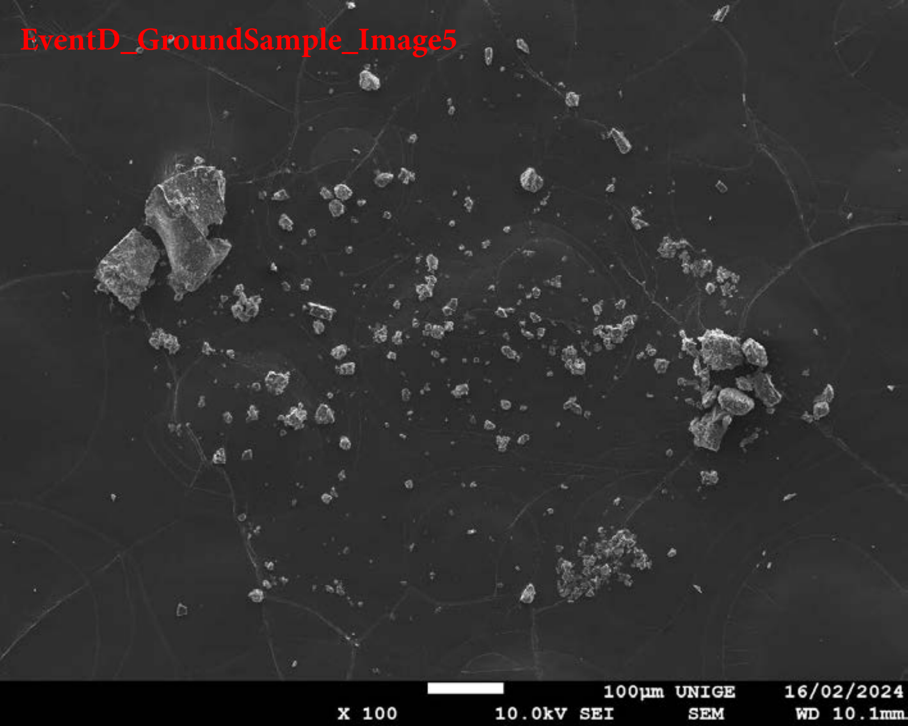
EventD_GroundSample_Image5
100µm UNIGE
16/02/2024
X 100
10.0kV SEI
SEM
WD 10.1mm

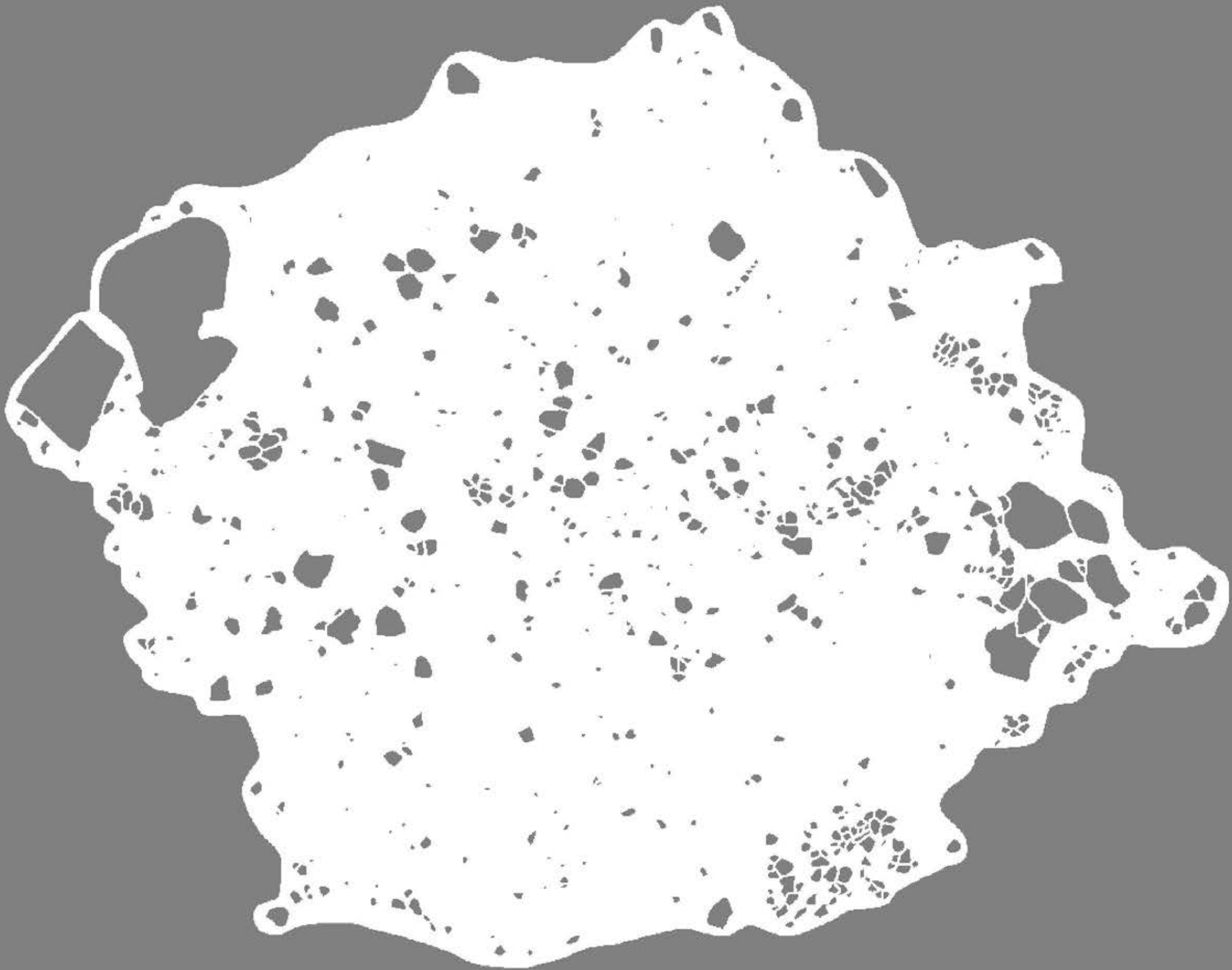

EventD_GroundSample_Image6

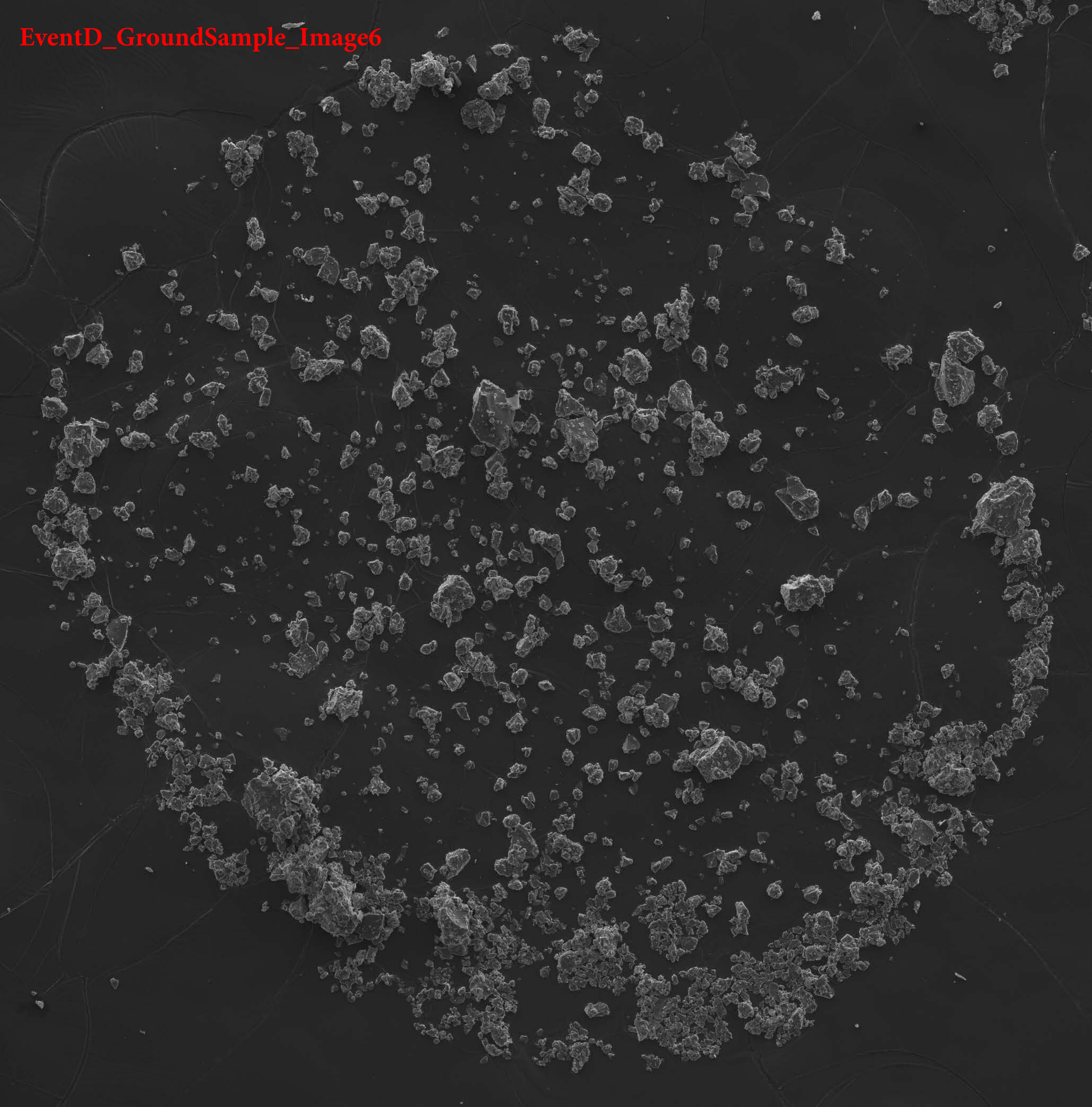

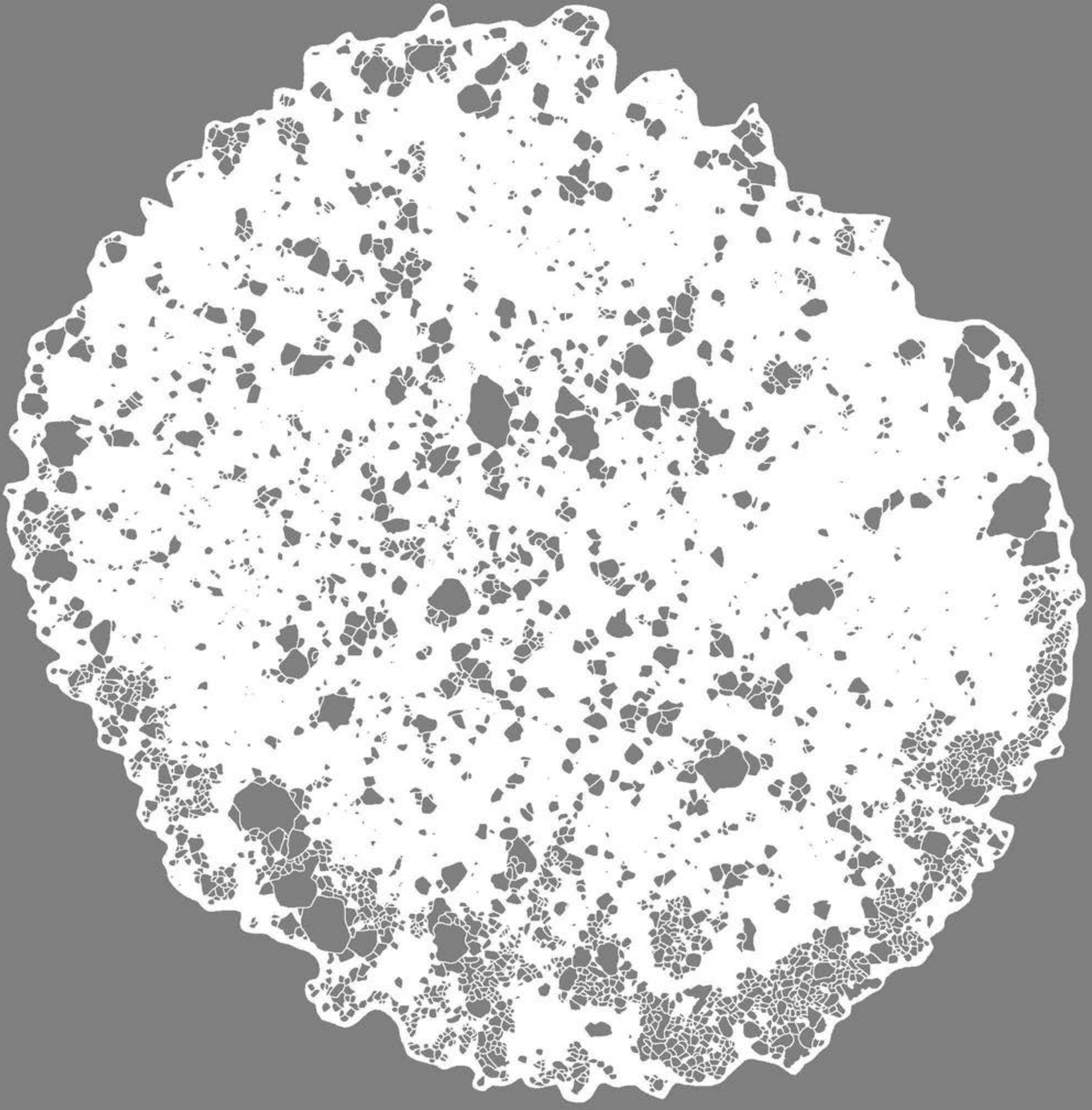

Supplement: Supplementary file 5 — Supplementary Information 5: Images of the collected samples and their segmented versions (Events A, B, C, and D). [file 41598_2026_45460_MOESM5_ESM.pdf]
